# The Influence of Secondary Interactions on the [N−I−N]^+^ Halogen Bond

**DOI:** 10.1002/chem.202102575

**Published:** 2021-09-06

**Authors:** Sofia Lindblad, Flóra Boróka Németh, Tamás Földes, Daniel von der Heiden, Herh G. Vang, Zakarias L. Driscoll, Emily R. Gonnering, Imre Pápai, Nathan Bowling, Mate Erdelyi

**Affiliations:** ^1^ Department of Chemistry – BMC Uppsala University 751 23 Uppsala Sweden; ^2^ Institute of Organic Chemistry Research Center for Natural Sciences Budapest Hungary; ^3^ Department of Chemistry University of Wisconsin-Stevens Point 2001 Fourth Avenue, Stevens Point Wisconsin 54481 USA; ^4^ Department of Chemistry University J. Selyeho 94505 Komárno Slovakia

**Keywords:** density functional calculations, halogen bond, iodonium ion, NMR spectroscopy

## Abstract

[Bis(pyridine)iodine(I)]^+^ complexes offer controlled access to halonium ions under mild conditions. The reactivity of such stabilized halonium ions is primarily determined by their three‐center, four‐electron [N−I−N]^+^ halogen bond. We studied the importance of chelation, strain, steric hindrance and electrostatic interaction for the structure and reactivity of halogen bonded halonium ions by acquiring their ^15^N NMR coordination shifts and measuring their iodenium release rates, and interpreted the data with the support of DFT computations. A bidentate ligand stabilizes the [N−I−N]^+^ halogen bond, decreasing the halenium transfer rate. Strain weakens the bond and accordingly increases the release rate. Remote modifications in the backbone do not influence the stability as long as the effect is entirely steric. Incorporating an electron‐rich moiety close by the [N−I−N]^+^ motif increases the iodenium release rate. The analysis of the iodine(I) transfer mechanism highlights the impact of secondary interactions, and may provide a handle on the induction of stereoselectivity in electrophilic halogenations.

## Graphical abstract

**Halogen bond‐stabilized** halonium ions are mild halenium transfer agents. The dependence of their reactivity on chelation, strain, steric hindrance and electrostatic interaction has been evaluated using NMR, DFT and kinetics experiments. The mechanistic analysis of the transfer is expected to provide a handle in the induction of stereoselectivity in electrophilic halogenations.

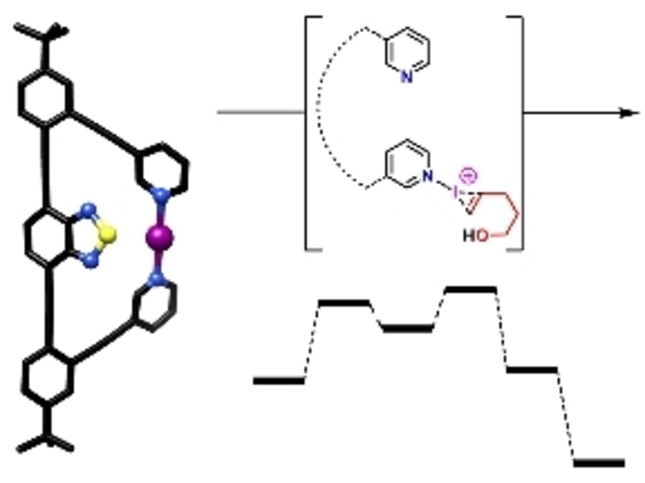

## Introduction

The halogen bond is the weak, attractive and directional interaction of halogens with Lewis bases.^[1]^ It has been studied as early as the 1860s,^[2]^ however, it has first received greater attention upon Odd Hassel's Nobel prize‐awarded crystallographic studies a century later,^[3]^ and has only recently become widely acknowledged.^[4]^ During the past decade it has found applications in a variety of fields including crystal engineering,^[5]^ material sciences,^[6]^ organic synthesis,^[7]^ and drug design,^[8]^ and has emerged as a complementary molecular tool to the hydrogen bond. It lately developed into one of the quickest growing areas of chemical research.

Halogen bonds stabilize reactive halenium ions.^[9]^ In their halogen bond complexes, the empty and accordingly highly electrophilic p‐orbital of a halogen(I) simultaneously receives electron density from two Lewis bases,^[10]^ and forms a remarkably strong three‐center, four‐electron [N−X−N]^+^ bond.^[11]^ For instance, the three‐center bond of the triiodide ion is of 180 kJ/mol strength.^[12]^ Three‐center, four‐electron halogen bond complexes receive increasing attention,[^9a^, ^10^, ^13^] not least because they are applicable as mild halonium transfer and oxidation agents in synthesis,[^7a^, ^7b^, ^7c^, ^7d^, ^7e^, ^7f^, ^7g^, ^7h^, ^7j^, ^7k^, ^7l^, ^7m^, ^7n^] as novel supramolecular synthons in the assembly of complex architectures,^[14]^ and as a key element in halogen bonded frameworks.^[15]^ Fundamental investigations primarily included symmetry determination, where the effect of varying the halogen,[^13a^, ^13d^] the solvent,^[10]^ the counter ion,^[13e]^ and the electron density of the Lewis bases[^13b^, ^13c^, ^14d^] have been assessed. The [N−X−N]^+^ bond was shown to prefer a static, symmetric geometry, which can be desymmetrized by using Lewis bases of different electron density.[^9b^, ^13c^, ^16^] The symmetric alteration of the electron density influences the halonium release rate, with the electron‐poor systems reacting quicker than the electron‐rich,^[13a]^ but not their symmetry or geometry.^[13b]^

**Figure 1:**
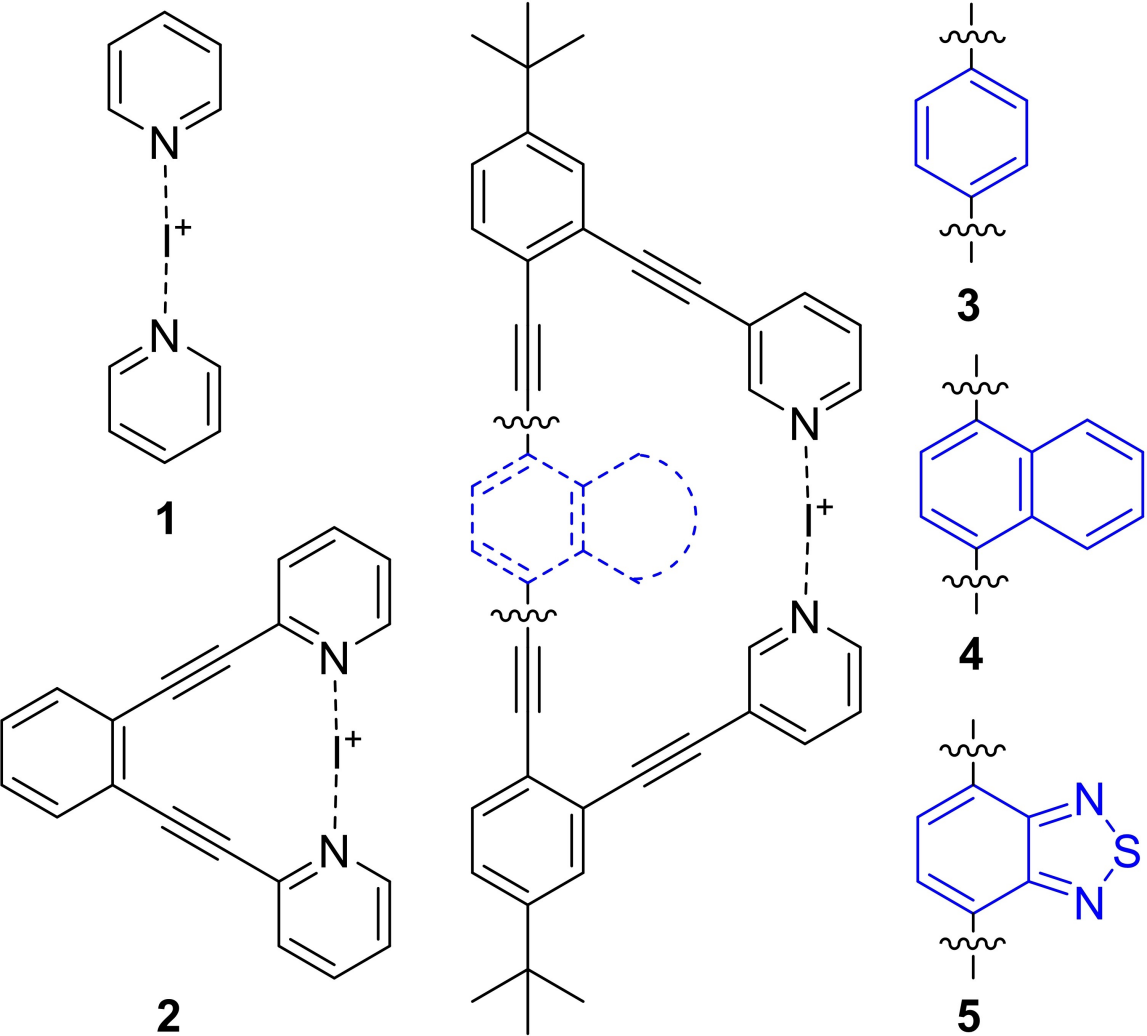
[Bis(pyridine)iodine(I)]^+^ complexes studied for the assessment of the impact of strain, steric hindrance and charge repulsion on the properties of the [N−I−N]^+^ three‐center bond.

Herein we present a systematic investigation of the influence of strain, steric and electronic effects on the three‐centered halogen bond using the model compounds **1**–**5**, shown in Figure 1. [Bis(pyridine)halogen(I)]^+^ (**1**) complexes are non‐chelating, and accordingly, the formation of their [N−X−N]^+^ complexes is associated with a larger entropic penalty. While they are stable in dry solutions, they easily dissociate and in the presence of other Lewis bases, they are involved in rapid chemical exchange processes, complicating the interpretation of their solution spectroscopic data.^[16a]^ [(1,2‐Bis(pyridine‐2‐ylethynyl)benzene)halogen(I)]^+^ (**2**) complexes have been developed for an accurate characterization of the three‐center, four‐electron halogen bond complexes in solution as these are entropically favorable, and thus their spectroscopic data is not influenced by exchange processes. The 1,2‐diethynylbenzene backbone^[17]^ enforces co‐planarity of the pyridine Lewis bases and a slightly longer (4.57 Å) than optimal (4.56 Å) inter‐nitrogen distance, marginally weakening the halogen bond.^[13a]^ Compounds **3**–**5** were designed to allow optimal inter‐nitrogen distance while posing different degrees of steric and electronic effects, thereby allowing the evaluation of whether these influence the reactivity of the halogen bonded halonium ion. We report our findings from the systematic NMR spectroscopic, reaction kinetics, and computational investigation of the influence of strain, steric and electronic effects on the [N−I−N]^+^ bond geometry and strength. An improved understanding of the influence of steric and electronic effects on the three‐center halogen bond is expected to enable the rational modulation of the reactivity of bis(pyridine)halogen(I)‐type halonium transfer synthetic agents, and facilitate their applications for instance in the design of complex supramolecular systems,[^14c^, ^14f^, ^14g^, ^18^] and in halogen‐bonded organic frameworks.^[15]^

## Results and Discussion

**Synthesis**

**Scheme 1:**
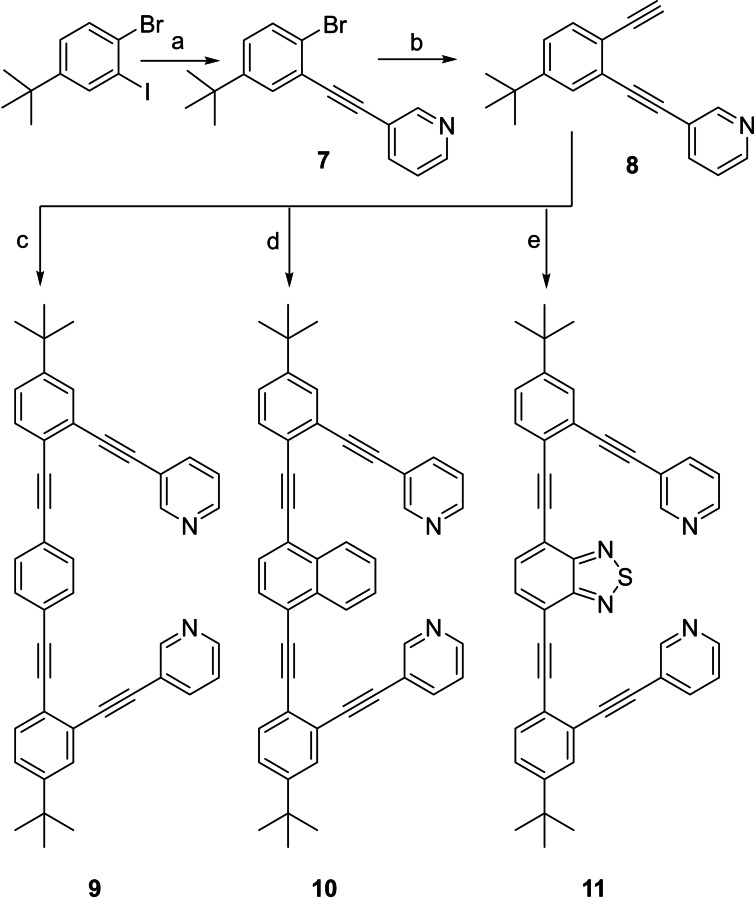
The synthetic routes to ligands **9**–**10**. Reagents and conditions: a) 3‐ethynylpyridine, Pd(PPh_3_)_4_, CuI, NEt_3_, dry THF, 50 °C, 18 h, Ar (g); b) 1. trimethylsilylacetylene, Pd(PPh_3_)_4_, CuI, NEt_3_, THF, 100 °C, 24 h, Ar (g), 2. TBAF, THF, −89 °C, 2 h; c) 1,4‐diiodobenzene, Pd(PPh_3_)_4_, CuI, NEt_3_, THF, 75 °C, overnight, Ar (g); d) 1,4‐dibromonaphthalene, Pd(PPh_3_)_4_, CuI, NEt_3_, DMF, 95 °C, 20 h, Ar (g); e) 4,7‐dibromo‐benzo[c][1,2,5]thiadiazole, Pd(PPh_3_)_4_, CuI, NEt_3_, DMF, 95 °C, 72 h, Ar (g).

Ligand **9** was prepared following a published procedure,^[19]^ while ligands **10** and **11** were achieved through modification of this route (Scheme 1). Hence, 1‐bromo‐2‐iodo‐4‐*tert*‐butyl‐benzene was converted to **7** through Sonogashira coupling with 3‐ ethynylpyridine. Subsequent Sonogashira coupling with trimethylsilylacetylene, followed by TMS‐deprotection with TBAF, yielded **8**, and a third Sonogashira coupling gave **9**, **10** and **11**, respectively, upon varying the aryl halide. 1,2‐Bis(pyridine‐2‐ylethynyl)benzene (**6**) was synthesized as reported.^[13a]^ Complex **1** ^[7c]^ is commercially available. The iodine(I) complexes **1**–**5** were synthesized following a previously established procedure.[^13a^, ^13e^] Hence, a ligand was mixed with AgBF_4_ in CH_2_Cl_2_, under dry conditions, to form the corresponding silver(I) complex. Upon addition of I_2,_ AgI precipitated and the iodine(I) complex was formed. Following filtration, the complex (**1**–**5**) is precipitated upon addition of dry *n*‐hexane.

**Structural analysis**

**Figure 2:**
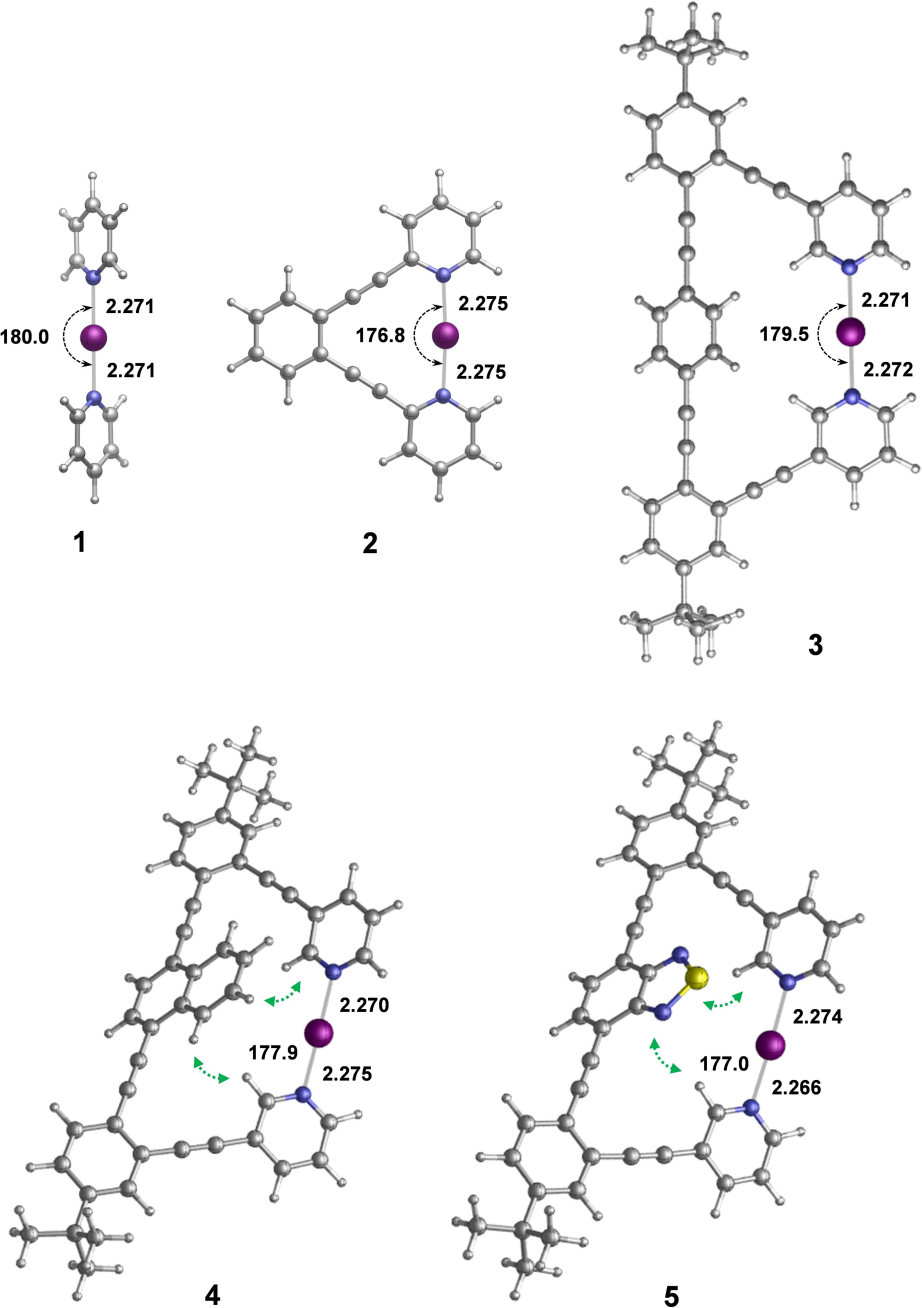
Structures of complexes **1**–**5** as obtained from DFT calculations. Computed N−I bond lengths are in Å, N−I−N bond angles in degrees. Stabilizing aryl‐aryl interactions are highlighted by green arrows. As weakly coordinating counterions have previously been shown not to influence the geometry of [N−I−N]^+^ complexes,^**[13e]**^ these were omitted in the computational models.

**Figure 3:**
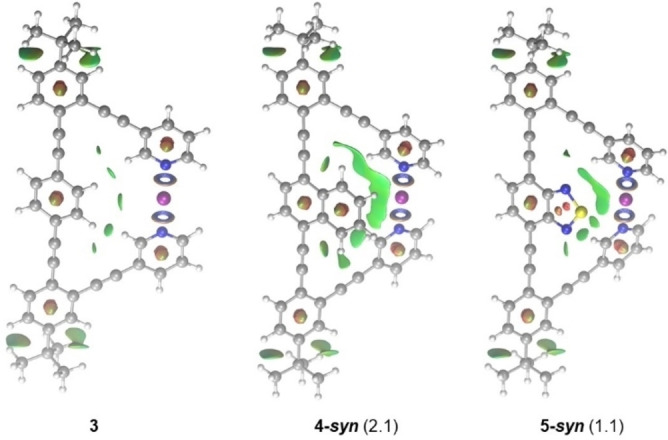
The non‐covalent interaction (NCI) plots of **3**, and of the *syn*‐geometries of **4** and **5**. The applied cutoff for reduced density gradient is set to s=0.4 au. Green isosurfaces represent weak stabilizing non‐covalent interactions. The three‐center, four‐electron halogen bond is illustrated by blue rings. Free energy differences between the *syn* and *anti* forms of **4** and **5** are given in parenthesis (in kcal/mol).

DFT calculations were carried out for structural analysis using the hybrid meta‐GGA M06‐2X functional and the SMD18 solvation model. For geometry optimizations and vibrational analysis, the Def2SVP basis set was employed, but single‐point energy calculations were performed with the more extended Def2TZVPP basis set (for computational details, see the Supporting Information). The DFT‐predicted ground state structures of complexes **1**–**5** are shown in Figure 2 (for computational details, see the Supporting Information). In good agreement with previous studies,[^9b^, ^13b^] the computed N−I bond distances show only a marginal variation along the series, and the ∼180° N−I−N bond angle is in line with that observed by X‐ray for **1** ^[20]^ and **2**.^[11]^ The linear arrangement and short N−I bond distance are typical for three‐center, four‐electron halogen bonds.^[9b]^ For complexes **3**–**5**, the N−I−N bond angle slightly deviates from 180°. In contrast to complex **2**,^[13a]^ the optimized structures of **3**–**5** deviate from planarity. The distortion of the conjugated aromatic system is already notable for complex **3**, but becomes even more significant for **4** and **5** (Figure 2), where the central bicyclic aromatic groups interact with the adjacent pyridines. Computations predict these *syn* conformers to be 1–2 kcal/mol favored over those with the naphthalene and benzo[*c*][1,2,5]thiadiazole rings tilted away from the [N−I−N]^+^ bond (*anti* arrangements), revealing these intramolecular aryl‐aryl contacts to be stabilizing (Figure S87, Supporting Information). This stabilization is found to be slightly higher for complex **4**, which is further corroborated by the non‐covalent interaction analysis of **3**–**5**, shown in Figure 3.

**Nitrogen coordination shifts**

The comparable ^15^N coordination shifts (Δ*δ* ^15^N_coord_=*δ* ^15^N_complex_−*δ* ^15^N_ligand_, Table 1)[^13b^, ^13e^, ^21^] of **1** and **3**–**5** compared with the slightly smaller Δδ^15^N_coord_ of **2** suggests that **3**–**5** adapt non‐strained geometries. This interpretation is further corroborated by their comparable N−I−N bond length and angle (Figure 2), and by the Δ*δ* ^15^N_coord_ of analogous systems.^[13b]^ We wish to emphasize that **3**–**5** are the first model systems that allow the investigation of the [N−I−N]^+^ three‐center halogen bond without the influence of strain or chemical exchange[^9b^, ^16a^] that complicate the interpretation of solution spectroscopic data.

**Table 1:** The ^15^N NMR Chemical Shift (*δ* ^15^N_complex_) and Coordination Shift (Δ*δ* ^15^N_coord_) of 1–5 and of the Corresponding Free Ligands (*δ* ^15^N_ligand_).

Structure	*δ* ^15^N_complex_	*δ* ^15^N_ligand_	Δ*δ* ^15^N_coord_
**1**	−175.1^[a]^	−67.0^[a]^	−108.1
**2**	−163.6	−64.5^[b]^	−99.1
**3**	−173.6	−65.5	−108.1
**4**	−173.8	−65.8	−108.0
**5**	−173.8	−65.7	−108.1

[a] Previously reported by us in ref. [13 e], and [b] ref. [13 a].

Based on their Δ*δ* ^15^N_coord_, **3**–**5** have comparable electron density at their [N−I−N]^+^ bond as **1**.

**Halogen release rates**

**Figure 4:**
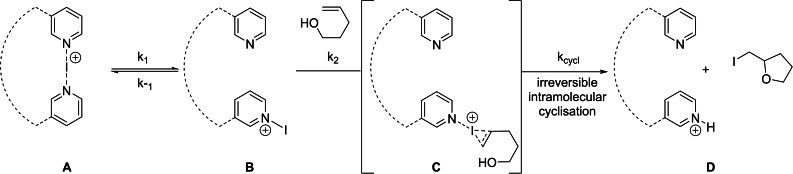
The rate of halocyclization of 4‐penten‐1‐ol in the presence of **1**–**5** was used to characterize their relative halenium release rate. A general, simplified mechanism, established in previous studies,[^7j^, ^22a^, ^22c^, ^22d^] for the initial steps of the reaction is shown.

**Figure 5:**
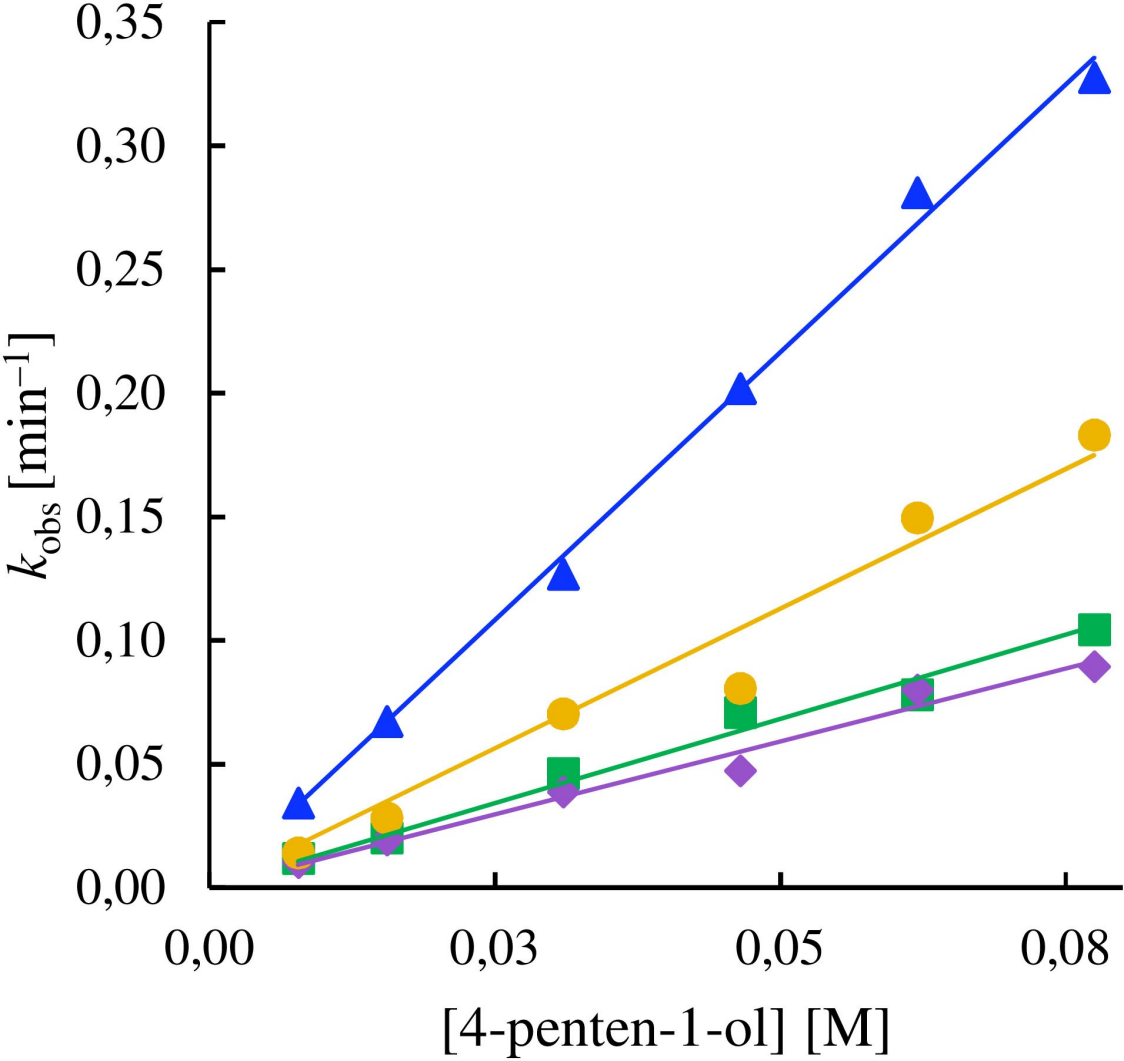
The second‐order‐rate constants (*k*_2_, M^−1^s^−1^) of **1**–**5** in iodocyclization with 4‐penten‐1‐ol in dry dicloroethane was obtained by fitting the observed pseudo‐first order rate constant (*k*_obs_) of each complex to the 4‐penten‐1‐ol concentration. The slopes of the corresponding graphs provided the *k*_2_ values 77.6 (R^2^=0.94) for **1**, 4.3 (blue) for **2**, 1.4 (green) for **3**, 1.2 (violet) for **4**, and 2.3 (yellow) for **5**. The plot for **1** is not shown here for clarity and is given in the Supporting Information (Figure S85).

To indirectly characterize halogen bond strength, similar to the previous investigation of related systems,^[13b]^ we measured the iodenium^[7c]^ release rates of **1**–**5** using the halocyclization of 4‐penten‐1‐ol in anhydrous 1,2‐dichloroethane as model reaction (Figure 4). The mechanism of this reaction has been examined experimentally.[^7j^, ^22^] The decrease of the UV signal of **1**–**5** throughout the reaction was followed, measuring pseudo‐first‐order rate constants for each complex in the presence of a large excess of olefin. To obtain the reaction rate constant (*k*_obs_), the data were fitted to the standard exponential model $A_{t}=A_{\infty }+\left(A_{0}-A_{\infty }\right)e^{-k_{obs}t}$ . Subsequently, the second‐order rate constants (*k*_2_) were obtained by determining the dependence of *k*_obs_ on the concentration of 4‐penten‐1‐ol (Figure 5). The latter describes the overall rate of the processes taking place ahead of the irreversible intramolecular cyclisation of the halonium‐substrate complex. We have shown that *k*_2_ of this reaction is modulated by the electronic effect of the pyridine substituents.^[13b]^ As the σ_m_=0.14 and ∋_p_=0.16 Hammett constants of alkynes are comparable,^[23]^ the backbones of **2**–**5** are assumed to not be responsible for the differences observed in their *k*_2_ (Figure 5). The highest iodenium release rate (*k*_2_=77.6 M^−1^s^−1^) was observed for **1**, which is non‐chelated and is known to dissociate comparably easily,^[16a]^ whereas **2**–**5** showed an order of magnitude slower reactions. Based on the Hammett parameters (**1**, σ_m_(‐H)=0.00; **2**–**5**, σ_m_(−CCH)=0.14 and σ_p_(−CCH)=0.16),^[23]^ **1** would be expected to have a slower reaction rate as compared to **2**–**5**. Thus, the observed lower iodination rate (*k*_2_) of **2**–**5** is due to chelation, and not to electronic effects. Chelation increases the stability of the [N−I−N]^+^ complex by lowering the entropic driving force of the halenium transfer process and the effective concentration of the mono‐coordinated halogen(I) species that halogen bonds to and subsequently reacts with 4‐penten‐1‐ol. Hence, the ligand dissociation has a significant influence on the reaction rate. Among the chelating ligands, **2** showed the quickest halenium release rate (*k*_2_=4.3 M^−1^s^−1^) that is most likely due to its non‐optimal geometry resulting in substantial strain (Figure 2), also detectable on its Δ*δ* ^15^N_coord_ (Table 1). The comparable *k*_2_ of **3** and **4** (*k*_2_=1.4 and 1.2 M^−1^s^−1^) suggests that the naphthalene ring of **4** does not considerably influence the [N−I−N]^+^ halogen bond despite its bulkiness. The twice as high halenium release rate of **5** (*k*_2_=2.3 M^−1^s^−1^) as compared to **3** and **4** suggests that the benzo[*c*][1,2,5]thiadiazole ring of **5** may have a through‐space interaction with the bis(pyridine)iodine(I) moiety. This may be due to the direct involvement of the benzo[*c*][1,2,5]thiadiazole in the halenium transfer process or by aryl‐aryl interaction with the pyridines (Figure 2), lowering the energy of the transition state. There may be an attractive Coulomb interaction between the partially positively charged iodonium ion and the electron‐rich sulfur of the benzo[*c*][1,2,5]thiadiazole, influencing the halenium release rate. Alternatively, the repulsion between the nonbonding electron pairs of the sulfur and the filled p‐orbitals of the iodine(I), whose charge is transferred into the pyridines to a large extent,[^9b^, ^13d^] may increase the halenium release rate. The latter is in line with the filled p‐orbitals of an iodine that participates in a halogen bond being able to act as Lewis base and hence as hydrogen bond acceptor^[24]^ or electron donor to silver(I).^[18a]^ Electrostatic repulsion between the bis(pyridine)iodine(I) moiety and the polarized central aromatic systems would provide repulsion in an analogous manner. The latter is supported by the electrostatic potential maps (EPSs) computed for **3**–**5** (Figure S88, Supporting Information), which indicate that the positive charge of iodine(I) is distributed over the [bis(pyridine)iodine(I)] moiety, and the central aromatic rings are indeed polarized as well. Even if it is not possible to differentiate between the above possibilities, the fact that the naphthalene of **4** does not influence *k*_2_ suggests that the effect of the benzo[*c*][1,2,5]thiadiazole of **5** is unlikely to be entirely steric. To gain further insight, we performed computational coordinate scans mapping the potential energy of **3**–**5** as a function of the central aromatic ring system orientation. Whereas the central benzene ring of **3** showed free rotation, the naphthalene and benzo[*c*][1,2,5]thiadiazole rings of **4** and **5**, respectively, were hindered by the [N−I−N]^+^ bond (Figures S89–91, Supporting Information).

**Computed stabilities**

DFT calculations were carried out to gain further insight into the above reactivities. The relative stabilities of iodine(I) complexes can be quantified by the thermodynamics of the hypothetical isodesmic reactions shown in Table 2. Although the phenyl(ethynyl)‐substituted pyridine units of the chelating ligands are slightly less basic than the unsubstituted pyridine (see computed proton affinities in Table 2), the chelate complexes **2**–**5** are predicted to be more stable than **1** (Δ*G*_iso_>0). This is clearly due to the entropic loss associated with the formation of the [bis(pyridine)iodine(I)] complex **1**. The strained nature of complex **2** is corroborated by the calculated equilibria, as this complex is found to be thermodynamically less favored than **3**–**5**. The latter systems are non‐strained and have comparable stabilities, indicating that the backbone modification has only a minor impact on the ground state halogen bond energies of iodine(I) chelate complexes. Overall, the computed Δ*G*_iso_ data are in qualitative agreement with the experimentally observed *k*_2_ trend **1**>**2**>**3**–**5**.

**Table 2:** Relative stabilities of complexes **1**–**5**.

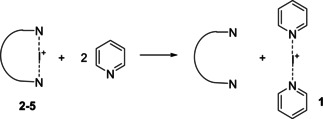		
Complex	ΔG_iso_ [kcal/mol]^[a]^	Proton affinity [kcal/mol]^[b]^
**1**	0.0	−278.1
**2**	3.9	−275.1
**3**	4.7	−275.3
**4**	4.6	−275.3
**5**	4.6	−275.3

[a] Relative stabilities are computed with respect to the [bis(pyridine)iodine(I)] complex **1** as Δ*G*_iso_ values of isodesmic reactions. [b] Proton affinities of pyridine and (phenyl)ethynyl‐substituted pyridines corresponding to chelating ligands (for details, see the Supporting Information).

**The mechanism of halocyclization**

**Figure 6:**
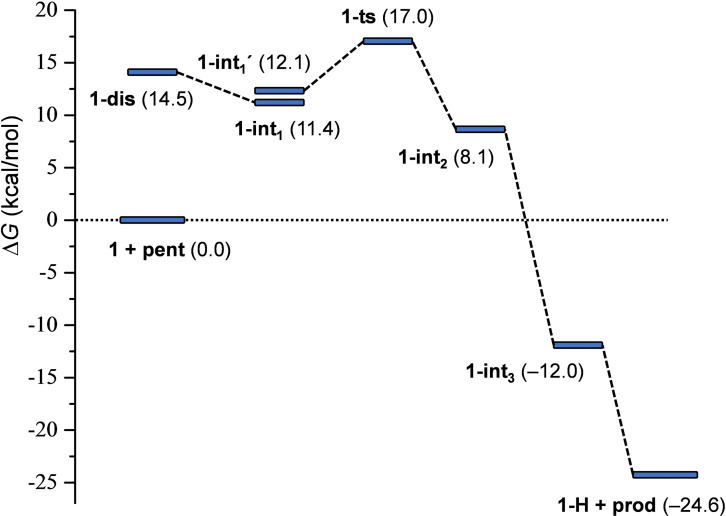
Free energy profile computed for the reaction of complex **1** with 4‐penten‐1‐ol (**pent**). Relative stabilities are shown in parentheses (in kcal/mol; with respect to the energy of 1+**pent**).

**Figure 7:**
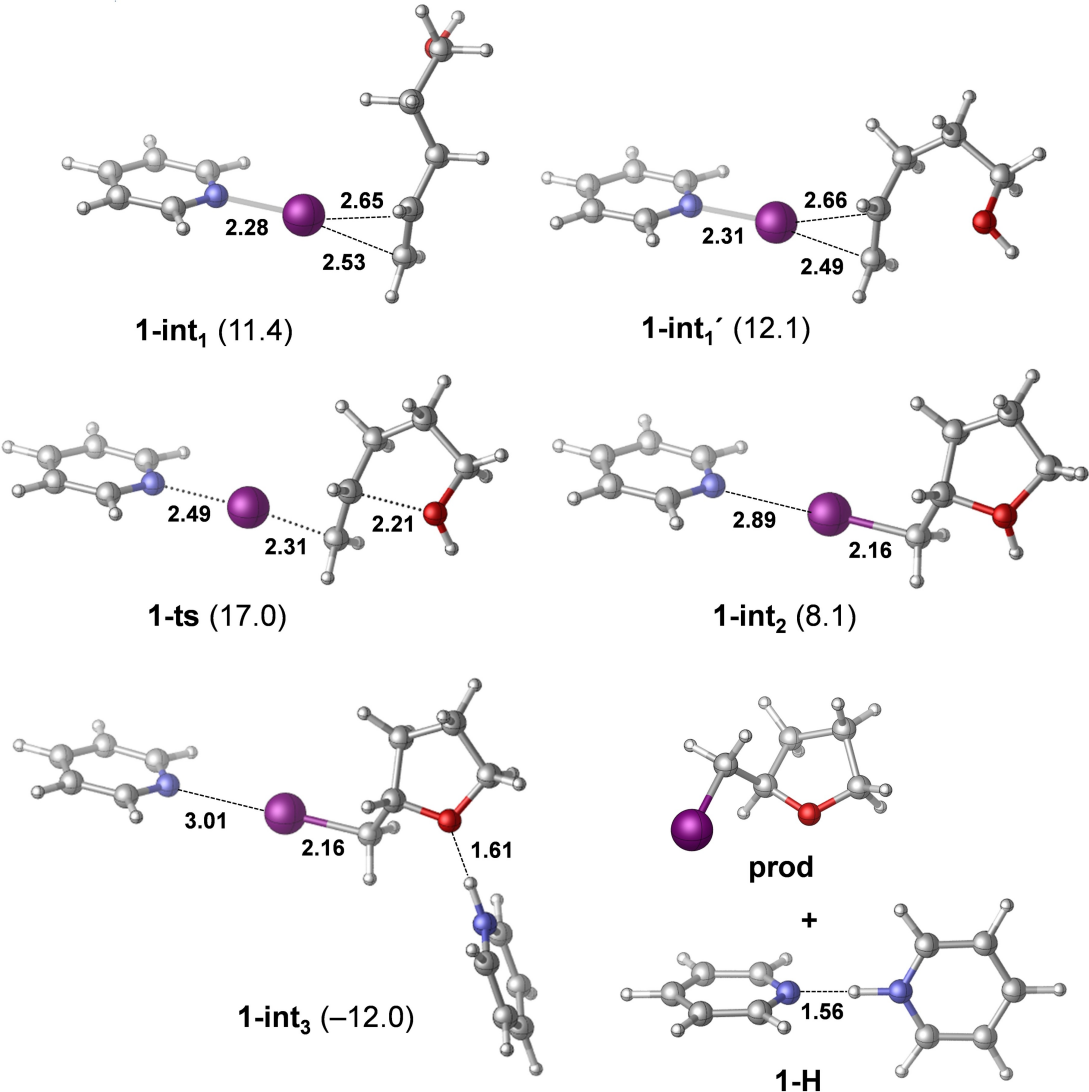
Optimized structures of species identified computationally for the reaction of complex **1** with 4‐penten‐1‐ol. Relative stabilities are shown in parentheses in kcal/mol, with respect to **1**+4‐penten‐1‐ol. Selected bond distances are given in Å.

**Figure 8:**
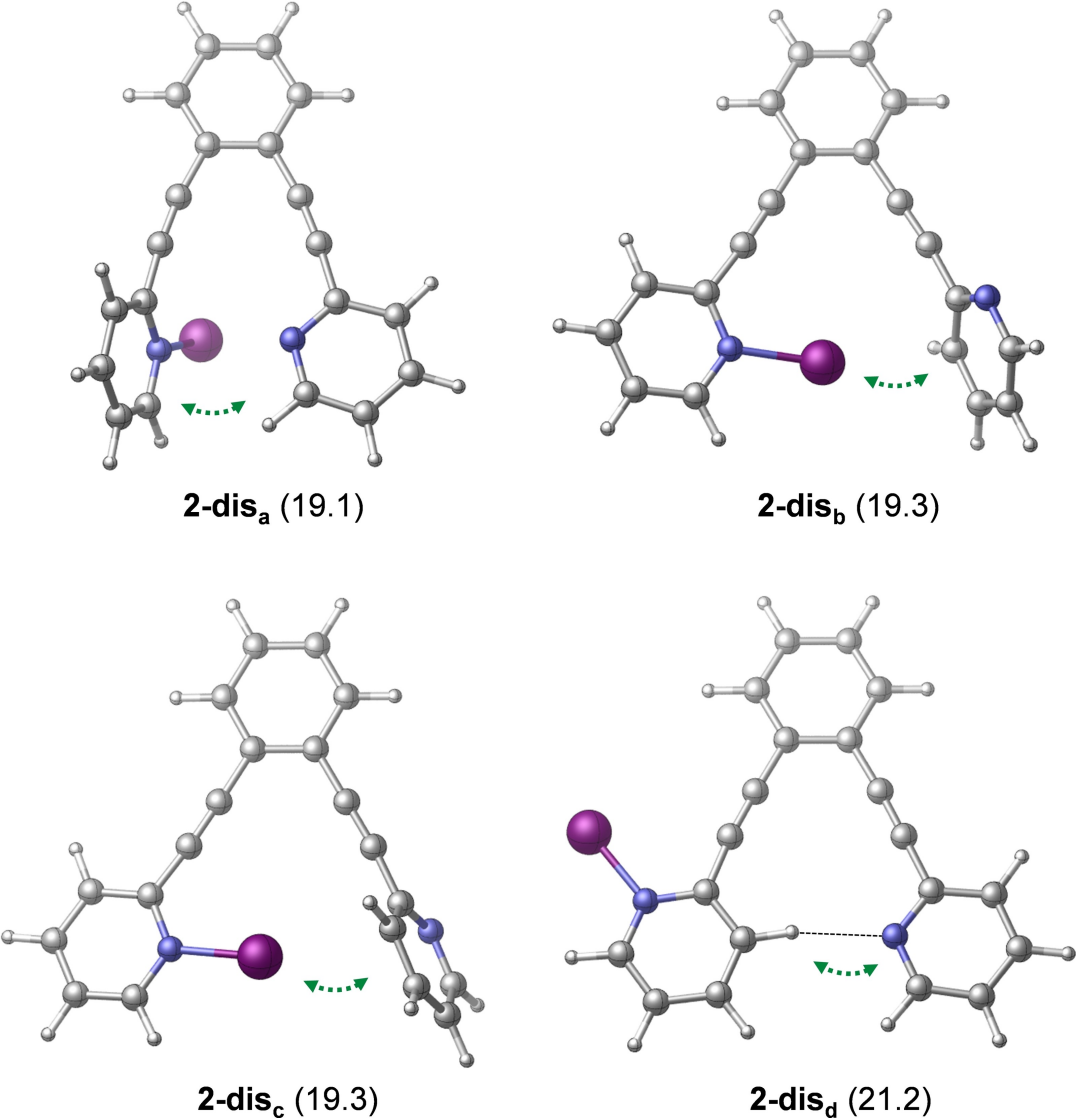
Dissociated forms of complex **2**. Relative stabilities are shown in parentheses (in kcal/mol; with respect to the symmetric chelated form). Selected bond distances are given in Å. Green dotted arrows indicate stabilizing non‐covalent interactions.

**Figure 9:**
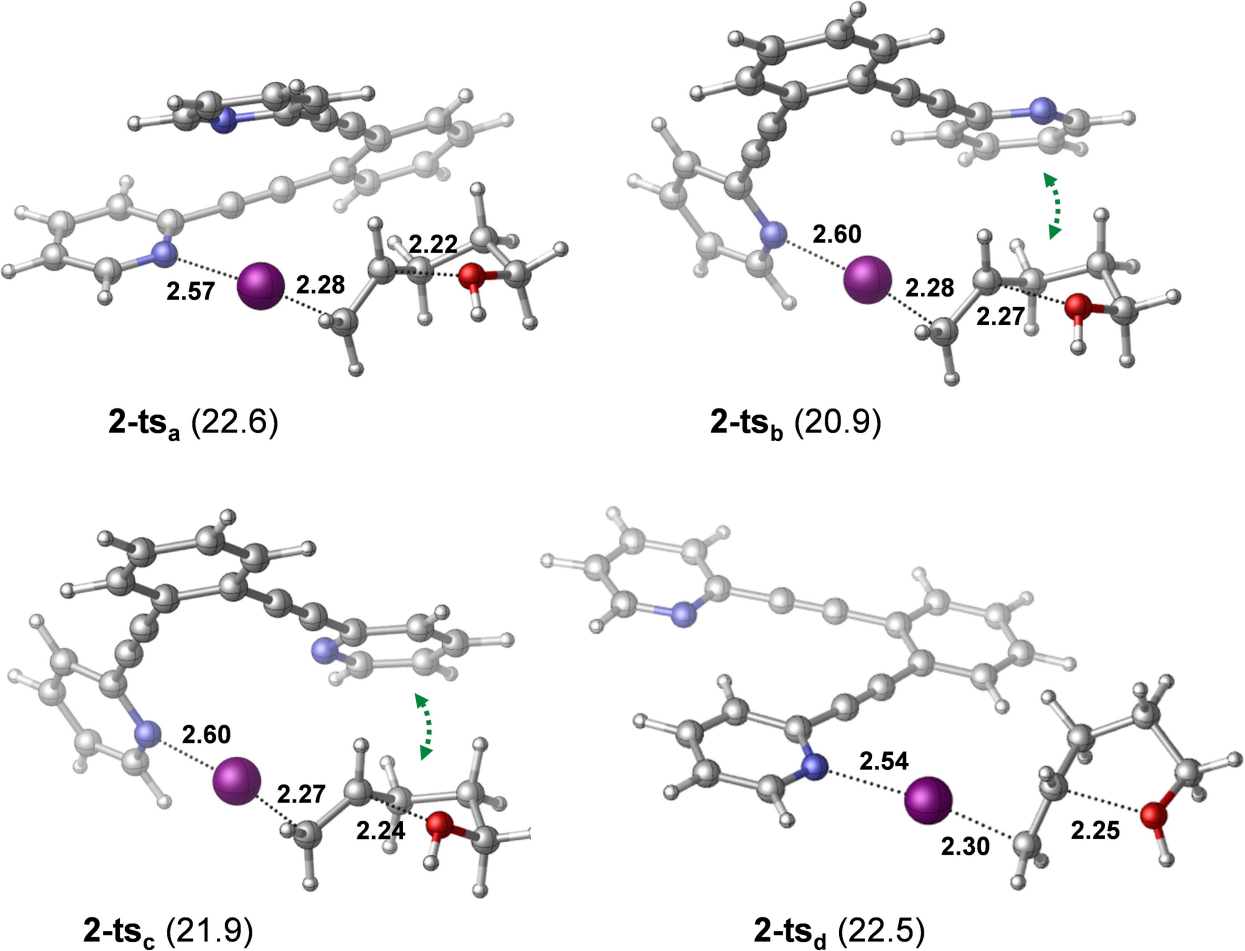
Transition states computed on various pathways in the reaction **2**+**pent**. Relative stabilities are shown in parentheses (in kcal/mol; with respect to reactant state). Selected bond distances are given in Å. Stabilizing non‐covalent interactions between the substrate and the displaced pyridine are highlighted by green dotted arrows.

**Figure 10:**
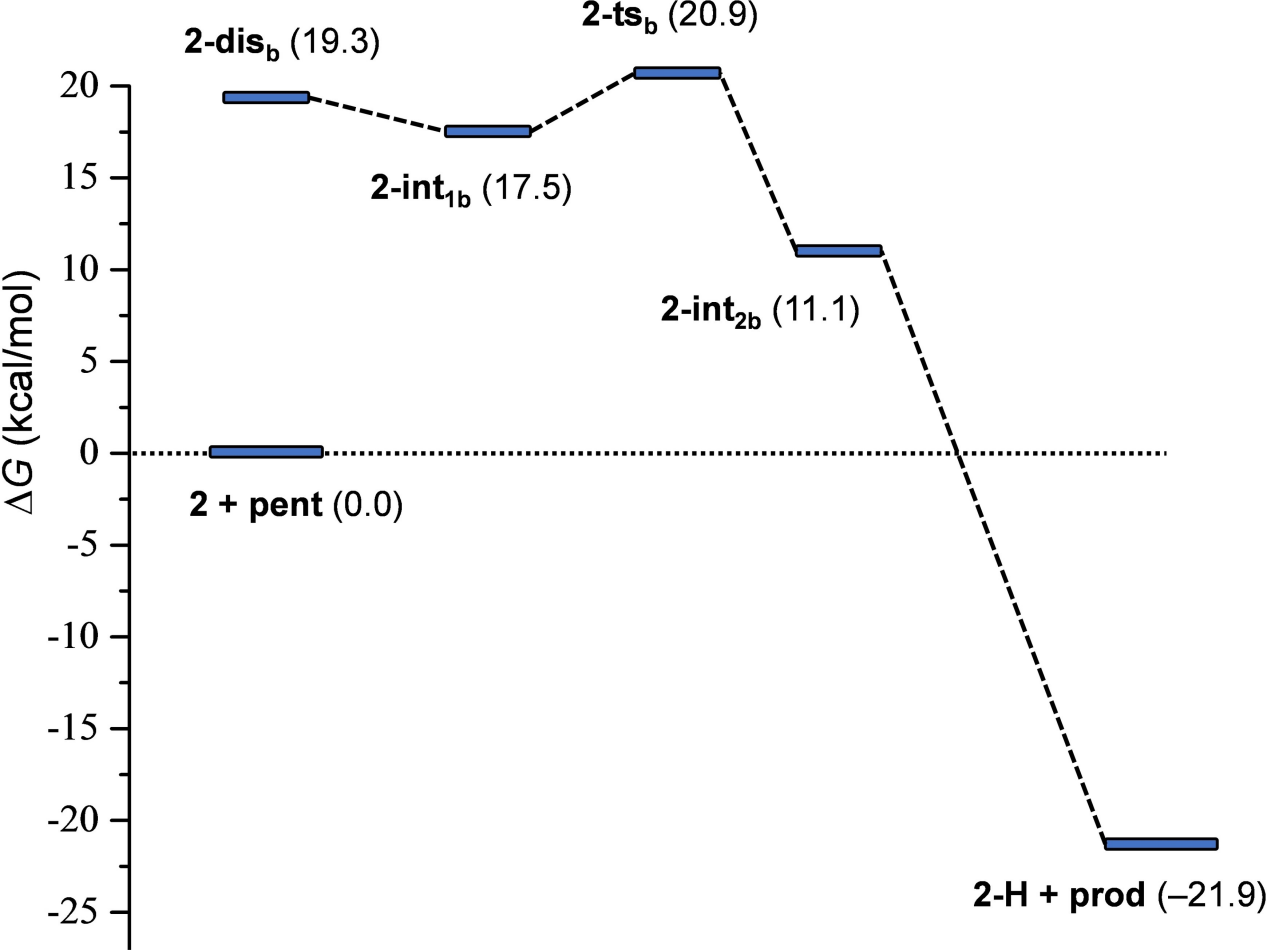
Free energy profile computed for the reaction of complex **2** with 4‐penten‐1‐ol. Relative stabilities are shown in parentheses (in kcal/mol; with respect to **2**+**pent**).

Next, we investigated the mechanism of iodocyclization upon the reaction of **1** with 4‐penten‐1‐ol (**pent**). The free energy profile corresponding to the most feasible reaction pathway is shown in Figure 6, and the optimized structures of the key states are illustrated in Figure 7. The reaction is initiated by the displacement of one of the pyridines from complex **1**. The dissociated state **1‐dis** (i. e., Py‐I^+^+Py) is predicted to be 14.4 kcal/mol less stable with respect to the reactants. Coordination of 4‐penten‐1‐ol via its double bond is thermodynamically favorable. The most stable form of this intermediate (**1‐int_1_**) lies at 11.4 kcal/mol on the free energy scale, and the conformer with the OH group preorganized for ring closure (**1‐int_1_′**) is only slightly less favored. The cyclization of 4‐penten‐1‐ol and the iodine(I) transfer takes place in a concerted manner via transition state **1‐ts**, which is predicted to be at 17.0 kcal/mol, yielding intermediate **1‐int_2_**. The deprotonation of this species by a pyridine is barrierless and highly exergonic. The dissociation of the iodoether molecule from **1‐int_3_** and the formation of the hydrogen‐bonded complex **1‐H** provides additional stabilization for the product. This reaction pathway is in line with the mechanism that has been previously proposed from kinetic measurements (Figure 4). The reaction pathway involving the association of the displaced pyridine with 4‐penten‐1‐ol via an O−H⋅⋅⋅N hydrogen‐bond prior to ring closure was found to be kinetically less favored. The transition state of this hydrogen bond assisted iodocyclization could be identified computationally to be at 18.5 kcal/mol, and is thus slightly less favoured than **1‐ts** (for details, see the Supporting Information). Our computations reveal that the 4‐penten‐1‐ol iodocyclization induced by **2** has a similar mechanism. The dissociated state of **2** corresponds to various energetically close‐lying asymmetric singly coordinated forms, which are displayed in Figure 8. The relative stability of these conformers range between 19.1 and 21.2 kcal/mol, and thus these are significantly less stable than the symmetric ground state complex. They all involve various intramolecular pyridine‐pyridine and iodine(I)‐pyridine contacts. 4‐Penten‐1‐ol iodocyclization pathways associated with all four dissociated forms of **2** (Figure 8) were explored and here they are denoted as routes *a*, *b*, *c* and *d*. We located an array of transition states along these pathways lying within a fairly narrow (2 kcal/mol) energy range (see Table 3 and Figure 9). The most favoured reaction pathways are associated with the **2‐dis_b_** and **2‐dis_c_** forms of the dissociated state, wherein the coordination of 4‐penten‐1‐ol is sterically hindered. Coordination to these complexes requires structural rearrangement; however, the cost of this structural change is counterbalanced, and even exceeded, by the stabilization arising from noncovalent substrate‐pyridine interactions. In complexes **2‐dis_a_** and **2‐dis_d_**, the iodine(I) is readily accessible, but the related intermediates and transition states do not benefit from stabilizing intermolecular contacts. The overall free energy profile of the most favored reaction pathway, shown in Figure 10, and also all other pathways, show close resemblance to that of reaction **1** with 4‐penten‐1‐ol (Figures 6 and 7). The iodocyclization transition state is preceded by a complex bearing an iodine(I) coordinated 4‐penten‐1‐ol (intermediate **2‐int_1b_**), and it is followed by a weakly bound cyclic intermediate **2‐int_2b_**, which gets deprotonated in a subsequent step. The deprotonation process has not been examined in detail, yet our computational analysis suggests that it may easily occur intramolecularly, that is, via deprotonation by the displaced pyridine of the ligand (Figure S99, Supporting Information).

**Table 3:** Relative stability of species identified computationally on various pathways in the reaction of complex **2** with 4‐penten‐1‐ol.^[a]^

pathway	2‐dis	2‐int_1_	2‐ts	2‐int_2_
***a***	19.1	18.6	22.6	13.7
***b***	19.3	17.5	20.9	11.1
***c***	19.3	17.9	21.9	13.3
***d***	21.2	19.0	22.5	13.6

[a] Pathways *a*, *b*, *c* and *d* are denoted according to the related dissociated states of complex **2** (**2‐dis_a_**, **2‐dis_b_**, **2‐dis_c_**, and **2‐dis_d_**, see Figure 8). States **2‐int_1_** and **2‐int_2_** refer to intermediates before and after transition states **2‐ts**. Relative stabilities are given in kcal/mol with respect to **2**+4‐penten‐1‐ol.

The free energy data computed for the two reactions (for complexes **1** and **2**; Figures 6 and 10) imply that the iodocyclization transition states represent the rate‐determining states. This is likely for the reaction of **1** with 4‐penten‐1‐ol, because **1‐ts** lies well above the **1‐dis** state. It is less evident for the corresponding reaction of **2**, where the relative stability of the dissociated states is more comparable to those of the iodocyclization transition states, for instance, **2‐dis_b_** lies only 1.6 kcal/mol below **2‐ts_b_**. Considering the uncertainty of the present methodology for free energy predictions, particularly in comparison of states with different molecularity, no firm conclusion can be drawn for the rate‐determining state. Nevertheless, calculations predict significantly higher barrier for the reaction with the chelated complex **2**, which can be clearly associated with the relative stabilities of complexes **1** and **2**. The free energy difference between **1‐ts** and **2‐ts_b_** (3.9 kcal/mol) is virtually identical to that obtained for the relative stabilities of the complexes (Table 2).

**Figure 11:**
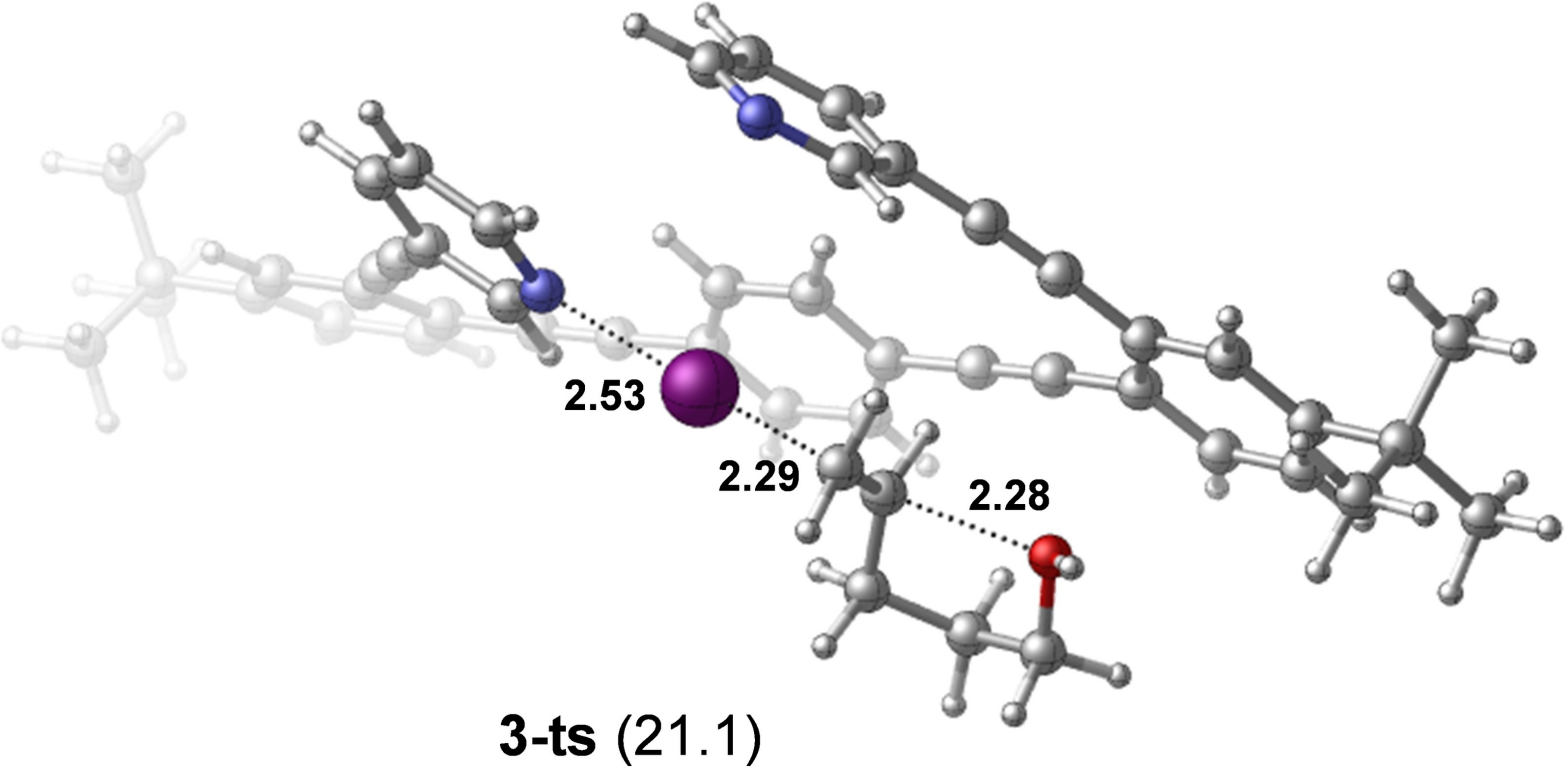
Transition state computed for the reaction of complex **3** with pent. Relative stability is given in parentheses (in kcal/mol; with respect to **3**+**pent**). Selected bond distances are given in Å.

The increased size and the flexibility of **3**–**5** prevented us from extensively exploring the mechanistic details of their iodocyclization; however, our computations indicate similar mechanistic features for their reactions, and similar reactivities to **2**. One representative transition state computed for the iodocyclization using **3** is shown in Figure 11. The transition state **3‐ts** is predicted to be at 21.1 kcal/mol in free energy. The corresponding dissociated state **3‐dis** is computed to be 19.8 kcal/mol less stable with respect to the symmetric chelated form of **3**. Due to the uncertainties of the applied computational approach, one cannot expect to be able to reproduce the differences in the experimental reaction rates of **3**–**5**. However, our computational analysis suggests that the difference in the reactivity of complexes **4** and **5** might be related to the intramolecular aryl‐aryl interactions discussed above. It indicates that the relative stabilities of the transition state of the iodocyclization reactions of **4** and **5** are barely influenced by the orientation of the naphthalene and benzo[*c*][1,2,5]thiadiazole rings (Figure S101, Supporting Information). However, the *syn*‐orientation of compound **4** is more stabilized (2.1 kcal/mol) as compared to that of **5** (1.1 kcal/mol, Figure 3) by intramolecular aryl‐aryl interaction, resulting in slightly higher barrier for the iodocylization reaction with **4**. Whereas this energy difference is minor, it is in line with the experimentally observed **5**>**4** reactivity trend (Figure 5).

## Conclusion

We evaluated the influence of chelation, ligand strain, charge repulsion and steric hindrance on the [N−I−N]^+^ halogen bond. The ^15^N NMR coordination shifts (Table 1) indicate that in contrast to complex **2**, the backbones of **3**–**5** adapt non‐strained geometries. This is of significance as **2** has originally been introduced^[13a]^ to evade ligand scrambling that is unavoidable for the halogen bond complexes of halonium ions with monodentate Lewis bases, such as **1**.^[25]^ Whether symmetric or asymmetric, ligand scrambling cannot be neglected at the interpretation of the solution spectroscopic data without risking the misinterpretation of their bonding situation.^[16]^ Chelation has a stabilizing effect, which is reflected by the lower iodenium^[7c]^ release rates of **2**–**5** as compared to **1** (Figure 5), and is corroborated by the computed stabilities (Table 2). Within a chelated complex, strain is destabilizing and accordingly increases the iodenium release rate, as demonstrated by the higher reactivity of **2** as compared to **3**–**5** (Figure 4). Steric crowding in itself does not influence halogen bond stability, which is reflected by the comparable halenium release rates of **3** versus **4** (Figure 4). The iodenium release rates of complexes **3**–**5** suggest that electronic effects, here modelled by the thiadiazole ‐ iodonium interaction of **5**, may have an influence on halonium ion reactivity, stronger than steric crowding, which in turn are comparable in **4** (naphthalene) and **5** (benzo[*c*][1,2,5]thiadiazole). Computations on the DFT level suggest that the mechanism of iodenium transfer in halocyclization with 4‐penten‐1‐ol is analogous for the chelated (**2**‐**5**) and non‐chelated (**1**) bis(pyridine)iodine(I) reagents. It involves the initial dissociation of the [N−I−N]^+^ complex (N^+^‐I+N), coordination of the alkene to the σ‐hole of the transient *N*‐pyridinium ion, followed by a concerted halocyclisation and iodenium transfer that constitutes the transition state of the process. The subsequent dissociation and proton transfer steps are barrierless. The improved understanding of this mechanism is expected to support, for instance, the development of robust and general methods for asymmetric halogenation of olefins, which is a long‐sought goal of synthetic organic chemistry.^[26]^ Moreover, understanding the influence of strain, steric and electronic effects along with that of chelation on the three‐center halogen bond of halonium ions will also enable the development of more complex supramolecular assemblies[^14a^, ^14b^, ^14c^, ^14f^] and halogen‐bonded organic frameworks (XOFs).^[15]^ It may in addition provide helpful knowledge for the development of structurally closely related organometallic reagents^[27]^ and systems held together by other types of three‐center, four‐electron bonds, such as tetrel, pnictogen and chalcogen bonds.^[11]^ The bis(pyridine)iodine(I) complex has lately been proposed to be involved in a spectacular cation‐cation interaction,[^18a^, ^28^] for which the influence of steric and electronic effects, strain, crystal packing forces and hydrophobic stacking are yet by far not well understood. The presented study of novel model systems that neither suffer from ligand scrambling or strain ought to support their further studies.

## Conflict of interest

The authors declare no conflict of interest.

## Supporting information

As a service to our authors and readers, this journal provides supporting information supplied by the authors. Such materials are peer reviewed and may be re‐organized for online delivery, but are not copy‐edited or typeset. Technical support issues arising from supporting information (other than missing files) should be addressed to the authors.

Supporting Information
