# Supplementary material for: The Influence of Secondary Interactions on the [N−I−N]+ Halogen Bond
Source: Chemistry. 2021 Sep 6;27(55):13748–56. doi: 10.1002/chem.202102575 (PMC8518683; doi:10.1002/chem.202102575)
Supplement: Supplementary file 1 — Supporting Information [file CHEM-27-13748-s001.pdf]

Chemistry–A European Journal

Supporting Information

The Influence of Secondary Interactions on the $[N{-}I{-}N]^+$ Halogen Bond

Sofia Lindblad, Flóra Boróka Németh, Tamás Földes, Daniel von der Heiden, Herh G. Vang, Zakarias L. Driscoll, Emily R. Gonnering, Imre Pápai,* Nathan Bowling,* and Mate Erdelyi*

TABLE OF CONTENTS

1. SYNTHESIS S2

2. STRUCTURES S5

3. NMR SPECTRA S6

4. KINETICS S28

4.1. General information S28

4.2. Kinetic data S28

5. COMPUTATIONS S44

5.1. Computational approach S44

5.2. Structural analysis of complexes **1-5** S45

5.3. Molecular electrostatic potentials of **3-5** S47

5.4. Potential energy scans for aryl rotations in **3-5** S47

5.5. Relative stabilities of complexes **1-5** S48

5.6. Iodocyclization of pentenol with complex **1** S49

5.7. Iodocyclization of pentenol with complex **2** S50

5.8. Iodocyclization transition states for complexes **3-5** S52

5.9. Total energy data S53

5.10. Cartesian coordinates S54

6. REFERENCES S100

The original UV kinetics data is available, free of charge at Zenodo at DOI: 10.5281/zenodo.5108510

1. SYNTHESIS

General methods. CH_2Cl_2 was dried by distillation over CaH_2, and *n*-hexane by distillation over Na, benzophenone and tetraglyme. For NMR, deuterated solvents were dried by adding 3 Å molecular sieves to freshly opened bottles. All dry solvents were stored over 3 Å molecular sieves in a glovebox. Pyridine was redistilled prior to use. All other chemicals were used without further purification. For all synthesis performed in a glovebox, glassware had been dried at 150 °C in an oven, or *in vacuo*, at least overnight. NMR spectra were recorded on a Bruker Avance Neo 500 MHz spectrometer equipped with a TXO cryogenic probe, or an Agilent MR-400 equipped with an OneNMR probe. Chemical shifts are reported on the δ scale (ppm), with the residual solvent signal as an internal reference; CD_2Cl_2 (δ_H 5.32, δ_C 53.84), $CDCl_3$ (δ_H 7.26, δ_C 77.16). Nitromethane (δ_N 0.0 ppm) was used as an external standard for ^{15}N. To assign the ^1H NMR resonances chemical shift (δ), multiplicity, coupling constants (*J* Hz) and number of hydrogens were considered. 2D spectra (^1H,^{15}N HMBC, ^1H,^{13}C HSQC, ^1H,^{13}C HMBC, TOCSY, and COSY) also aided correct assignment. Multiplicities are denoted as s (singlet), d (doublet), t (triplet), q (quartet), h (heptet), and m (multiplet). MestReNova 12.0.2. was used to process the NMR spectra.

Synthesis of bis(pyridine)iodine(I) tetrafluoroborate (1).[1-3] In a glovebox, $AgBF_4$ (0.602 g, 3.09 mmol) and pyridine (0.5 mL, 6.18 mmol) was dissolved in dry CH_2Cl_2 (8 mL) by stirring. Dry *n*-hexane (16 mL) was added to cause precipitation. The vial was centrifuged for 10 min at 2000 rpm, after which the supernatant was removed and the solid dried *in vacuo* overnight. The white solid was then dissolved in dry CH_2Cl_2 (8 mL), and I_2 (0.706 g, 2.78 mmol) was added. The mixture was stirred vigorously for 30 min, allowing AgI to precipitate. The vial was then centrifuged for 10 min at 2000 rpm, and the supernatant was transferred to another vial. Dry *n*-hexane (16 mL) was added, causing **1** to precipitate. After centrifuging again for 10 min at 2500 rpm, the supernatant was removed and the remaining precipitate dried *in vacuo* for 30 min to generate **5** as a fluffy, white solid (1.026 g, 2.76 mmol, 99%). ^1H NMR (500 MHz, 25 °C, CD_2Cl_2) δ=8.78 (AA' part of AA'BB'C, 2H, H2/6), 8.22 (C part of AA'BB'C, 1H, H4/4'), 7.63 (BB' part of AA'BB'C, 2H, H3/5); ^{13}C NMR (125 MHz, CD_2Cl_2) δ=146.9 (C-2, C-6), 142.7 (C4), 128.2 (C-3, C-5). HR(ESI)MS calcd for $C_{10}H_{10}IN_2^+$ for *m/z* 288.9989, found 288.9812.

Synthesis of (1,2-bis(pyridin-2-ylethynyl)benzene)iodine(I) tetrafluoroborate (2).[1-3] In a glovebox, $AgBF_4$ (0.071 g, 0.36 mmol) and 6 (0.101 g, 0.36 mmol) was dissolved in dry CH_2Cl_2 (8 mL) by stirring. Dry *n*-hexane (16 mL) was added to cause precipitation of the Ag^+ complex. The vial was centrifuged for 10 min at 2500 rpm, before the supernatant was removed and the precipitate (0.147 g, 0.31 mmol) dried *in vacuo* overnight. Dry CH_2Cl_2 (8 mL) was then added to dissolve the solid, and I_2 (0.093 g, 0.37 mmol) was added. The solution was stirred for 30 min, allowing AgI to precipitate. After centrifuging for 10 min at 2500 rpm, the supernatant was transferred to another vial, and dry *n*-hexane (16 mL) was added to cause precipitation of 2. The vial was centrifuged for another 10 min at 2500 rpm, and the supernatant was removed by syringe. The remaining solid was then dried *in vacuo* for 1 h to generate 2 as a white, fluffy solid (0.140 g, 0.28 mmol, 78 %). ^1H NMR (500 MHz, 25 °C, CD_2Cl_2) δ=8.85 (dd, *J* = 5.7, 1.5 Hz, 2H, H2), 8.17 (ddd, *J* = 7.8, 7.7, and 1.5 Hz, 2H, H4), 7.90 (dd, *J* = 7.8, 1.4 Hz, 2H, H5), 7.78 (AA' part of AA'BB', 2H, H10), 7.59 (BB' part of AA'BB', 2H, H11), 7.49 (ddd, *J* = 7.7, 5.7, 1.4 Hz, 2H, H3); ^{13}C NMR (126 MHz, CD_2Cl_2) δ=151.26 (C2), 143.04 (C6), 142.72 (C4), 134.86 (C10), 131.45 (C11), 130.90 (C5), 127.03 (C3), 124.63 (C9), 99.08 (C8), 91.09 (C7); ^{15}N NMR (51 MHz, 25 °C, CD_2Cl_2) δ −163.6 (N1). HRMS calcd for $(C_{20}H_{12}IN_2)^+$ m/z 407.0040 [M-BF_4^-]$^+$; found 407.0067.

Synthesis of (1,4-bis((4-(*tert*-butyl)-2-(pyridin-3-ylethynyl)phenyl)ethynyl)benzene)iodine(I) tetrafluoroborate (3). In a glovebox, $AgBF_4$ (0.016 g, 0.08 mmol) and **9** (0.050 g, 0.08 mmol) was dissolved in dry CH_2Cl_2 (8 mL), causing formation of a white precipitate. Dry *n*-hexane (16 mL), was added to ensure full precipitation. The mixture was centrifuged three sequential times; for 10 min at 2000 rpm, for 20 min at 2500 rpm, and for 30 min at 2500 rpm. Afterwards, most of the precipitation had sedimented to the bottom. The supernatant was removed, and the white solid Ag(I) complex (0.052 g, 0.07 mmol) was dried *in vacuo* overnight. The next day CH_2Cl_2 (8 mL) was added along with I_2 (0.015 g, 0.06 mmol), and the mixture was left stirring vigorously for 1 h. This generated precipitation of AgI as well as **3**. The mixture was centrifuged for 10 min at 2000 rpm, which caused AgI to sediment to the bottom while **3** still flew in the supernatant. The latter was transferred to another vial, and dry *n*-hexane (16 mL) was added, which caused the **3** to precipitate better and start to sediment to the bottom. The vial was centrifuged for 10 min at 2500 rpm, after which the clear, yellow supernatant was removed and the precipitate was dried *in vacuo* for 25 min to generate a mother-of-pearl colored solid (0.038 g, 0.05 mmol, 63 %). ^1H (500 MHz, 25 °C, CD_2Cl_2) δ=8.84 (m, 2H, H6), 8.80 (m, 2H, H2), 8.30 (m, 2H, H4), 7.72 (s, H18/19), 7.69 (m, 2H, H3), 7.68 (m, 2H, H10), 7.59 (m, 1H, H13), 7.51 (m, 2H, H12), 1.36 (s, 18H, H21); ^{13}C NMR (126 MHz, 25 °C, CD_2Cl_2) δ=153.0 (C11), 151.5 (C6), 148.6 (C2), 143.7 (C4), 132.4 (C13), 132.1 (C18/19), 129.8 (C10/), 128.3 (C3), 128.0 (C12), 125.4 (C5), 123.9 (C17), 123.5 (C14), 123.3 (C9), 96.8 (C8), 93.1 (C16), 90.4 (C15), 86.1 (C7), 35.3 (C20), 31.1 (C21); ^{15}N NMR (51 MHz, 25 °C, CD_2Cl_2) δ=−173.6 (N1).

Synthesis of (1,4-bis((4-(*tert*-butyl)-2-(pyridin-3-ylethynyl)phenyl)ethynyl)naphthalene)iodine(I) tetrafluoroborate (4). In a glovebox, $AgBF_4$ (0.016 g, 0.08 mmol) and 10 (0.050 g, 0.08 mmol) was dissolved in dry CH_2Cl_2 (8 mL). I_2 (0.022 g, 0.09 mmol) was added to the clear, blue-yellow solution, which was then stirred for 1 h 5 min to allow AgI to precipitate. The mixture was centrifuged for 10 min at 2500 rpm, and the cloudy supernatant was transferred to another vial (4 precipitated along with the AgI and was only slightly lighter). Dry *n*-hexane (16 mL) was added, causing better precipitation of 4. After centrifuging for 10 min at 2500 rpm, the clear, orange supernatant was removed, and the precipitate dried *in vacuo* for 1 h to generate 4 as a green-yellow solid (0.054 g, 0.06 mmol, 75 %). ^1H NMR (500 MHz, 25 °C, CD_2Cl_2) δ=8.73 (m, 2H, H22), 8.70 (m, 2H, H2), 8.41 (m, 2H, H6), 8.36 (m, 2H, H4), 7.94 (s, 2H, H19) 7.78 (d, *J* = 1.7 Hz, 2H, H10), 7.71 (d, *J* = 8.3, 2H, H13), 7.66 (dd, *J* = 8.0 and 5.6 Hz, 2H, H3), 7.63 (m, 2H, H23), 7.58 (dd, *J* = 8.3 and 1.7 Hz, 2H, H12), 1.40 (s, 18H, H21); ^{13}C NMR (126 MHz, 25 °C, CD_2Cl_2) δ=153.1 (C11), 151.7 (C6), 148.5 (C2), 144.1 (C4), 133.7 (C18), 132.4 (C13), 130.6 (C19), 130.2 (C10), 129.1 (C23), 128.1 (C3/12), 126.8 (C22), 125.1 (C5), 123.50 (C9), 123.49 (C14), 122.1 (C17), 97.0 (C8), 94.9 (C15), 91.3 (C16), 86.0 (C7), 35.3 (C20), 31.2 (C21); ^{15}N NMR (51 MHz, 25 °C, CD_2Cl_2) δ=−173.8 (N1).

Synthesis of (4,7-bis((4-(tert-butyl)-2-(pyridin-3-ylethynyl)phenyl)ethynyl)benzo[c][1,2,5]thiadiazole)iodine(I) tetrafluoroborate (5). In a glovebox, $AgBF_4$ (0.019 g, 0.10 mmol) and 11 (0.051 g, 0.08 mmol) was dissolved in dry CH_2Cl_2 (8 mL). I_2 (0.022 g, 0.09 mmol) was added to the clear, green-yellow solution, which was then stirred for 50 min to allow AgI to precipitate. The mixture was centrifuged for 10 min at 2500 rpm, and the cloudy, green-brown supernatant was transferred to another vial (5 precipitated along with the AgI and

but was much lighter). Dry *n*-hexane (16 mL) was added, causing better precipitation of 5. After centrifuging for 10 min at 2500 rpm, the clear green-orange supernatant was removed, and the precipitate dried *in vacuo* for 1 h to generate 5 as a yellow solid (0.059 g, 0.07 mmol, 88 %). ^{1}H NMR (500 MHz, 25 °C, CD_2Cl_2) δ=8.72 (m, 2H, H2), 8.58 (m, 2H, H6), 8.34 (m, 2H, H4), 8.00 (s, 2H, H19), 7.76 (d, *J* = 2.0, 2H, H10), 7.69 (d, *J* = 8.3 Hz, 2H, H13), 7.66 (dd, *J* = 8.0 and 5.6 Hz, 2H, H3), 7.57 (dd, *J* = 8.3 and 2.0 Hz, 2H, H12), 1.39 (s, 18H, H21); ^{13}C NMR (126 MHz, 25 °C, CD_2Cl_2) δ=155.5 (C18), 153.6 (C11), 151.9 (C6), 148.4 (C2), 144.0 (C4), 133.5 (C19), 132.6 (C13), 130.0 (C10), 128.1 (C3), 128.0 (C12), 125.4 (C5), 123.8 (C9), 123.0 (C14), 117.7 (C17), 96.5 (C8), 96.0 (C15), 89.2 (C16), 86.4 (C7), 35.4 (C20), 31.1 (C21); ^{15}N NMR (51 MHz, 25 °C, CD_2Cl_2) δ=−47.7 (N22), −173.8 (N1).

Synthesis of 1,2-bis(pyridin-2-ylethynyl)benzene (6).[2] $Pd(PPh_3)_2Cl_2$ (0.542 g, 0.77 mmol) and CuI (0.161 g, 0.85 mmol) was added to a microwave vial and kept under Ar (g). Et_3N (12 mL, 86.10 mmol), 1,2-diiodobenzene (1 mL, 7.65 mmol) and DMF (4 mL) was added by syringe, and the mixture was shortly stirred. 2-ethynylpyridine (1.8 mL, 17.82 mmol) was then added, followed by immediate irradiation by microwave for 9 min at 120 °C. The black reaction mixture was filtered through celite, washing with CH_2Cl_2 (~120 mL). EDTA (0.560 g, 1.92 mmol) was added, and the solution stirred for 55 min before it was filtered through celite again, washing with more CH_2Cl_2 (~50 mL). The solution was then washed with H_2O (115 mL), and the resulting aqueous phase was extracted 3 times with CH_2Cl_2 (tot. ~100 mL). The combined organic phases were concentrated *in vacuo* to give a black liquid crude, which was purified two sequential times on silica using column chromatography, eluting with CH_2Cl_2:EtOAc 95:5→80:20 and EtOAc:hexanes 2:3→7:3, respectively. This generated **6** as an orange solid (1.366 g, 4.87 mmol, 64 %). ^{1}H NMR (400 MHz, 25 °C, $CDCl_3$) δ=8.64 (m, 2H, H2), 7.63-7.72 (m, 6H, H5, H4, and H10), 7.37 (BB' part of AA'BB', 2H, H11/), 7.27 (m, 2H, H3). ^{13}C NMR (126 MHz, CD_2Cl_2) δ=150.71 (C2), 143.82 (C6), 136.67 (C4), 132.80 (C10), 129.44 (C11), 128.29 (C5), 125.88 (C3), 123.60 (C9), 93.66 (C7), 87.72 (C8); HRMS calcd for $(C_{20}H_{13}N_2)^+$ *m/z* 281.1073, found 281.1070.

Synthesis of 3-((2-bromo-5-(tert-butyl)phenyl)ethynyl)pyridine (7).[4] 1-Bromo-2-iodo-4-*tert*-butylbenzene[5] (5.0 g, 14.75 mmol) was dissolved in a mixture of dry THF (20 mL) and freshly distilled NEt_3 (20 mL). Argon was bubbled through this mixture for 20 minutes before $Pd(PPh_3)_4$ (0.85 g, 0.74 mmol) and CuI (0.14 g, 0.74 mmol) were added**.** Argon was bubbled through this mixture for an additional five minutes before 3-ethynylpyridine (1.52 g, 14.75 mmol) was added. The tube was sealed under argon and heated at 50 °C for 18 hours. After cooling to room temperature, the mixture was rinsed into a separatory funnel with CH_2Cl_2. The organic mixture was washed with NH_4Cl solution, dried with anhydrous $MgSO_4$, filtered and concentrated. Purification by flash chromatography (10% EtOAc/90% hexane on silica) yielded the product as an oily, yellow solid (3.95 g, 12.6 mmol, 85% yield). ^{1}H (400 MHz, 25 °C, $CDCl_3$) δ=8.82 (s, 1H), 8.57 (d, *J* = 4.8 Hz, 1H), 7.86 (d, *J* = 7.8 Hz, 1H), 7.58 (s, 1H), 7.53 (d, *J* = 8.5 Hz, 1H), 7.26 (m, 2H), 1.32 (s, 9H); ^{13}C (100 MHz, 25 °C, $CDCl_3$) δ=152.3, 150.5, 148.8, 138.5, 132.1, 130.5, 127.5, 124.1, 123.0, 122.4, 120.2, 91.7, 89.6, 34.6, 31.1. HRMS (APCI-QTOF): *m/z* $[M+H]^+$ Calcd for $C_{17}H_{17}{}^{79/81}BrN^+$ 314.0544/316.0524; found: 314.0554/316.0535.

Synthesis of 3-((5-(*tert*-butyl)-2-ethynylphenyl)ethynyl)pyridine (8).[4] Bromoarene **7** (3.95 g, 12.6 mmol) was dissolved in a mixture of triethylamine (20 mL) and THF (20 mL) and added to an oven-dried reaction tube. Argon was bubbled through this mixture for 20 minutes. $Pd(PPh_3)_4$ (0.728 g, 0.63 mmol) and CuI (0.120 g, 0.63 mmol) were added and argon was bubbled through the mixture for another five minutes. After trimethylsilylacetylene (2.83 mL, 20.0 mmol) was added, the tube was sealed under Ar and heated at 100 °C for 24 hours. After cooling to room temperature, the mixture was rinsed into a separatory funnel with with CH_2Cl_2. The organic mixture was washed with NH_4Cl solution, dried with anhydrous Na_2SO_4, filtered and concentrated. A short flash column (silica, 25% ether/75% hexane) was run to remove residual catalysts. Appropriate fractions were concentrated and this TMS-protected alkyne product was dissolved in 100 mL THF. After chilling to −89 °C, TBAF (12 mL 1M in THF, 12.6 mmol) was added dropwise and the mixture was stirred for two hours at this temperature. Ammonium chloride solution was added before the mixture was allowed to warm to room temperature. This aqueous mixture was extracted twice with EtOAc. The combined organic extracts were dried with Na_2SO_4, filtered and concentrated. After purification by flash chromatography (silica, 25% ether/75% hexane) the product was revealed as a light brown solid (2.39 g, 9.22 mmol, 73% yield over two steps). ^{1}H (400 MHz, 25 °C, $CDCl_3$) δ=8.81 (s, 1H), 8.56 (d, *J* = 4.3 Hz, 1H), 7.85 (d, *J* = 7.8 Hz, 1H), 7.57 (s, 1H), 7.49 (d, *J* = 8.2 Hz, 1H), 7.35 (d, *J* = 8.2 Hz, 1H), 7.28 (m, 1H), 3.32 (s, 1H), 1.33 (s, 9H); ^{13}C (100 MHz, °C, $CDCl_3$) δ=152.4, 152.1, 148.7, 138.5, 132.4, 128.9, 125.9, 125.1, 123.0, 121.9, 120.5, 91.6, 89.3, 82.1, 80.6, 34.8, 31.0. HRMS (APCI-QTOF): *m/z* $[M+H]^+$ Calcd for $C_{19}H_{18}N^+$ 260.1439; found 260.1461.

Synthesis of 1,4-bis((4-(*tert*-butyl)-2-(pyridin-3-ylethynyl)phenyl)ethynyl)benzene (9). As described previously,[6] 3-((5-(tert-butyl)-2-ethynylphenyl)ethynyl)pyridine[4] (**8**) (0.050 g, 0.19 mmol) was added to an oven-dried storage tube with $Pd(PPh_3)_4$ (5.1 mg, 0.0044 mmol) and CuI (0.84 mg, 0.0044 mmol). Oxygen rich air was replaced with argon atmosphere using Schlenk techniques. After 1,4-diiodobenzene (0.029 g, 0.088 mmol), NEt_3 (10 mL), and THF (10 mL) were added, the tube was sealed and heated at 75° C overnight. The contents were cooled to room temperature, then rinsed into a separatory funnel with CH_2Cl_2. The organic mixture was washed with NH_4Cl solution, dried with anhydrous Na_2SO_4, filtered and concentrated. The crude residue was purified via flash chromatography (silica gel) starting with a 0.1% CH_3OH/99.9% CH_2Cl_2 mobile phase, with a gradual increase in polarity to 1.5% CH_3OH/98.5% CH_2Cl_2. The product was isolated as an off-white solid (0.034 g, 0.057 mmol, 64% yield). ^{1}H (500 MHz, 25 °C, CD_2Cl_2) δ=8.80 (m, 2H, H6), 8.55 (m, 2H, H2), 7.86 (m, 2H, H4), 7.64 (d, *J* = 2.0 Hz, 2H, H10), 7.54 (s, 2H, H18/19), 7.53 (d, *J* = 8.2 Hz, 2H, H13), 7.43 (dd, *J* = 8.2 and 2.0 Hz, 2H, H12), 7.32 (dd , *J* = 7.9 and 4.9 Hz, 2H, H3), 1.36 (s, 18H, H21); ^{13}C NMR (126 MHz, 25 °C, CD_2Cl_2) δ=152.6 (C6), 152.5 (C11), 149.2 (C2), 138.7 (C4), 132.1 (C13), 132.0 (C18/19), 129.5 (C10), 126.6 (C12), 125.0 (C9), 123.7 (C3), 123.6 (C17), 123.0 (C14), 93.0 (C16), 92.0 (C8), 90.5 (C15), 89.9 (C7), 35.2 (C20), 31.2 (C21); ^{15}N NMR (51 MHz, 25 °C, CD_2Cl_2) δ=−65.5 (N1). HRMS (APCI-QTOF): *m/z* $[M+H]^+$. Calcd for $C_{44}H_{36}N_2H^+$ 593.2957; found: 593.2960

Synthesis of 1,4-bis((4-(*tert*-butyl)-2-(pyridin-3-ylethynyl)phenyl)ethynyl)naphthalene (10). To an oven-dried storage tube was added 3-((5-(tert-butyl)-2-ethynylphenyl)ethynyl)pyridine[4] (**8**) (0.40 g, 1.54 mmol), 1,4-dibromonaphthalene (0.200 g, 0.70 mmol), triethylamine (10 mL), and *N*,*N*-dimethylformamide (1 mL). Argon was bubbled through this mixture for 15 minutes before $Pd(PPh_3)_4$ (40.0 mg, 0.035 mmol) and CuI (7.0 mg, 0.035 mmol) were added. The tube was sealed and heated under argon at 95 °C for 20 hours. After cooling to room temperature, the mixture was rinsed into a separatory funnel with CH_2Cl_2. The organic mixture was washed with NH_4Cl solution, dried with anhydrous Na_2SO_4, filtered and concentrated. The crude product was loaded onto a 40% EtOAc/60% hexane column (silica gel) using a small amount of CH_2Cl_2. The polarity was gradually increased to 50% EtOAc/50% hexane, eventually yielding

the product as an off-white powder (0.325 g, 0.506 mmol, 72% yield). ^{1}H (500 MHz, 25 °C, CD_2Cl_2) δ=8.81 (m, 2H, H6), 8.57 (m, 2H, H2), 8.56 (m, 2H, H22), 7.84 (m, 2H, H4), 7.79 (s, 2H, H19), 7.71 (d, *J* = 2.1 Hz, 2H, H10), 7.66 (d, *J* = 8.2 Hz, 2H, H13), 7.48 (dd, *J* = 8.2 and 2.1 Hz, 2H, H12), 7.29 (m, 2H, H3), 7.22 (m, 2H, H23), 1.38 (s, 18H, H21); ^{13}C NMR (126 MHz, 25 °C, CD_2Cl_2) δ=152.8 (C6), 152.6 (C11), 149.3 (C2), 139.0 (C4), 133.3 (C18), 132.3 (13), 130.3 (C19), 129.8 (C10), 127.9 (C23), 127.0 (C22), 126.7 (C12), 124.9 (C9), 123.5 (C3), 123.1 (C14), 122.0 (C17), 120.6 (C5), 95.2 (C15), 92.3 (C8), 91.4 (C16/), 89.9 (C7), 35.2 (C20), 31.2 (C21); ^{15}N NMR (51 MHz, 25 °C, CD_2Cl_2) δ=−65.8 (N1). HRMS (APCI-QTOF): *m/z* $[M+H]^+$ Calcd for $C_{48}H_{39}N_2^+$ 643.3113; found: 643.3122.

Synthesis of 4,7-bis((4-(*tert*-butyl)-2-(pyridin-3-ylethynyl)phenyl)ethynyl)benzo[c][1,2,5]thiadiazole (11). To an oven-dried storage tube was added 3-((5-(tert-butyl)-2-ethynylphenyl)ethynyl)pyridine[4] (**8**) (0.40 g, 1.54 mmol), 4,7-dibromobenzo[c][1,2,5]thiadiazole (0.206 g, 0.70 mmol), triethylamine (10 mL), and *N*,*N*-dimethylformamide (1 mL). Argon was bubbled through this mixture for 20 minutes before $Pd(PPh_3)_4$ (40.0 mg, 0.035 mmol) and CuI (7.0 mg, 0.035 mmol) were added. The tube was sealed and heated under argon at 95 °C for 3 days. After cooling to room temperature, the mixture was rinsed into a separatory funnel with CH_2Cl_2. The organic mixture was washed with NH_4Cl solution, dried with anhydrous Na_2SO_4, filtered and concentrated. The crude product was loaded onto a 50% EtOAc/50% hexane column (silica gel) using a small amount of CH_2Cl_2. The purified product was isolated as a fluorescent yellow solid (254 mg, 0.39 mmol, 55% yield). ^{1}H NMR (500 MHz, 25 °C, CD_2Cl_2) δ=8.87 (m, 2H, H6), 8.56 (m, 2H, H2), 7.93 (m, 2H, H4), 7.83 (s, 2H, H19), 7.69 (d, *J* = 2.1 Hz, 2H, H10), 7.65 (d, *J* = 8.2 Hz, 2H, H13), 7.47 (dd, *J* = 8.2 and 2.1 Hz, 2H, H12), 7.31 (dd, *J* = 7.7 and 5.1 Hz, 2H, H3), 1.37 (s, 18H, H21); ^{13}C NMR (126 MHz, 25 °C, CD_2Cl_2) δ=154.8 (C18), 153.1 (C11), 152.9 (C6), 149.3 (C2), 139.0 (C4), 133.0 (C19), 132.6 (C13), 129.7 (C10), 126.7 (C12), 125.3 (C9), 123.4 (C3), 122.5 (C14), 120.7 (C5), 117.6 (C17), 96.4 (C15), 91.8 (C8), 90.3 (C7), 89.3 (C16), 35.3 (C20), 31.2 (C21); ^{15}N NMR (51 MHz, 25°C, CD_2Cl_2) δ=−45.9 (N22), −65.7 (N1). HRMS (APCI-QTOF): *m/z* $[M+H]^+$ calcd for $C_{44}H_{35}N_4S^+$ 651.2582; found: 651.2590.

2. STRUCTURES

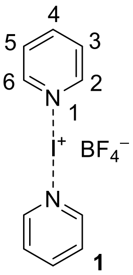

1

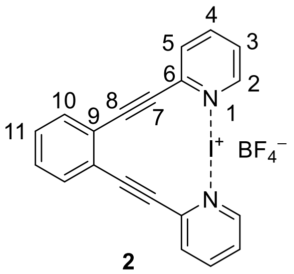

2

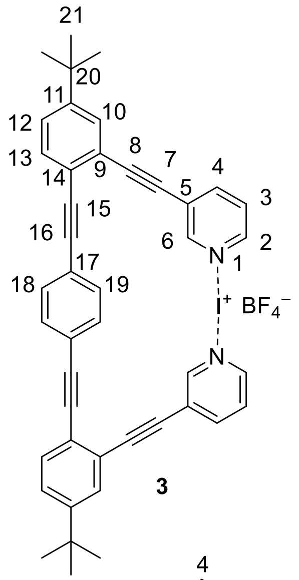

3

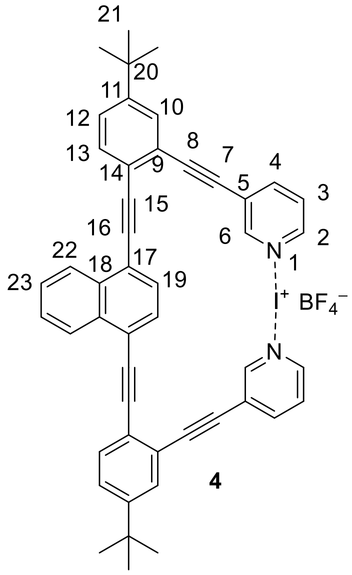

4

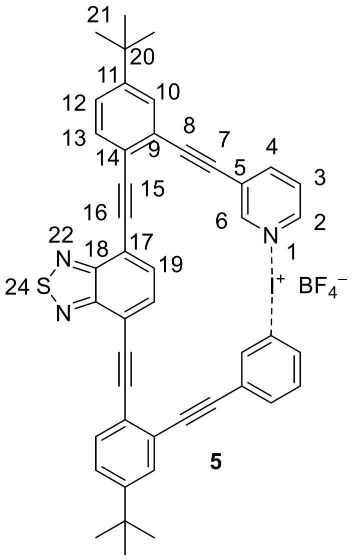

5

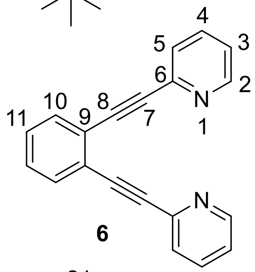

6

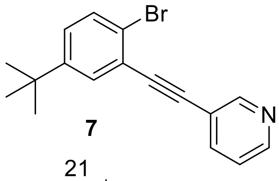

7

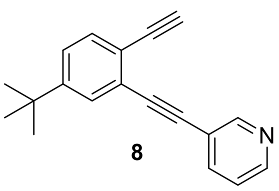

8

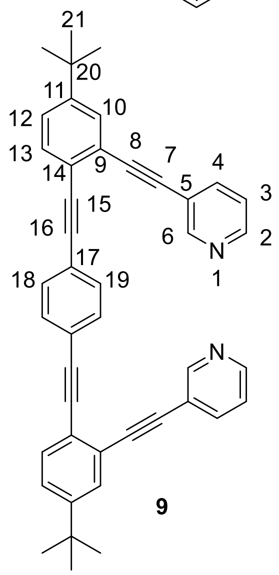

9

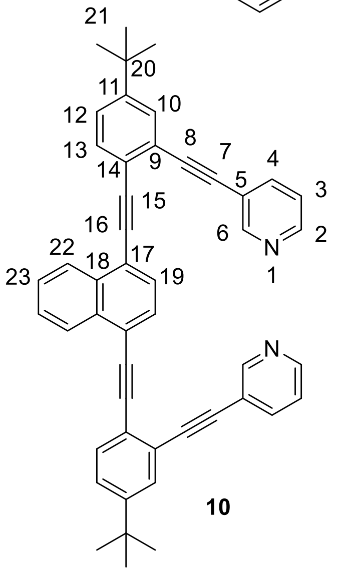

10

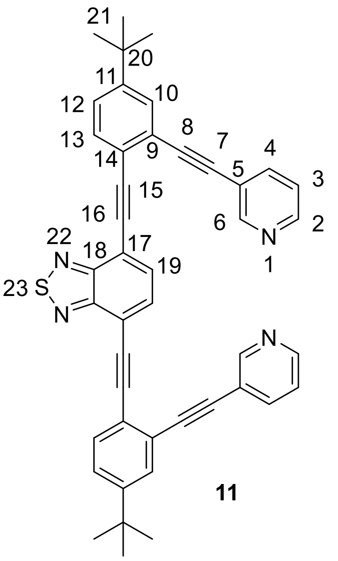

11

3. NMR SPECTRA

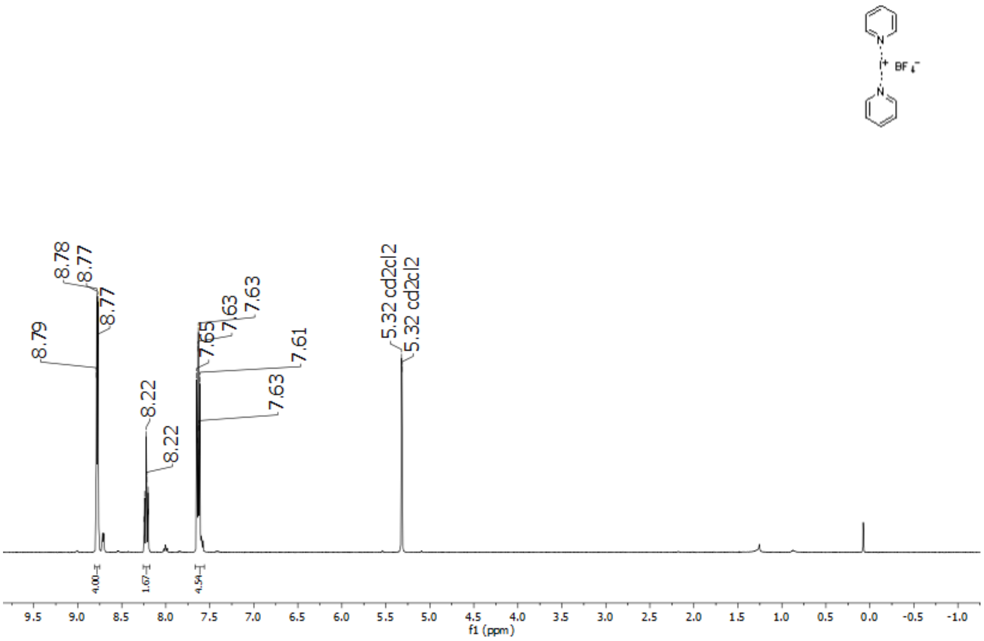

Figure S1. ^{1}H NMR spectrum of **1** (400 MHz, CD_2Cl_2, 25 °C).

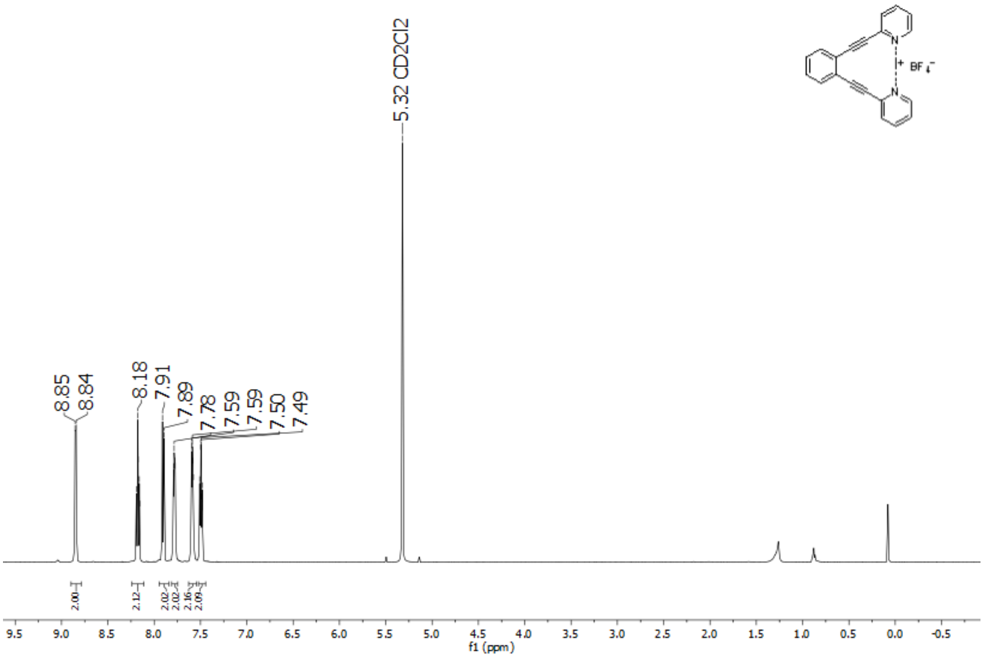

Figure S2. ^{1}H NMR spectrum of **2** (500 MHz, CD_2Cl_2, 25 °C).

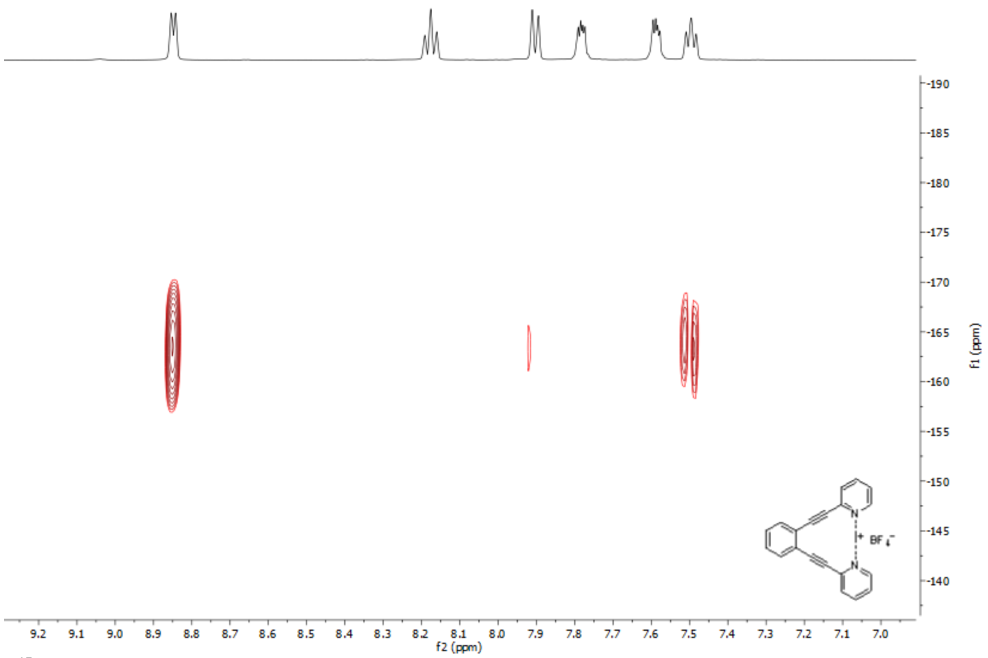

Figure S3. $^{1}H,^{15}N$ HMBC spectrum of **2** (500 and 51 MHz, CD_2Cl_2, 25 °C).

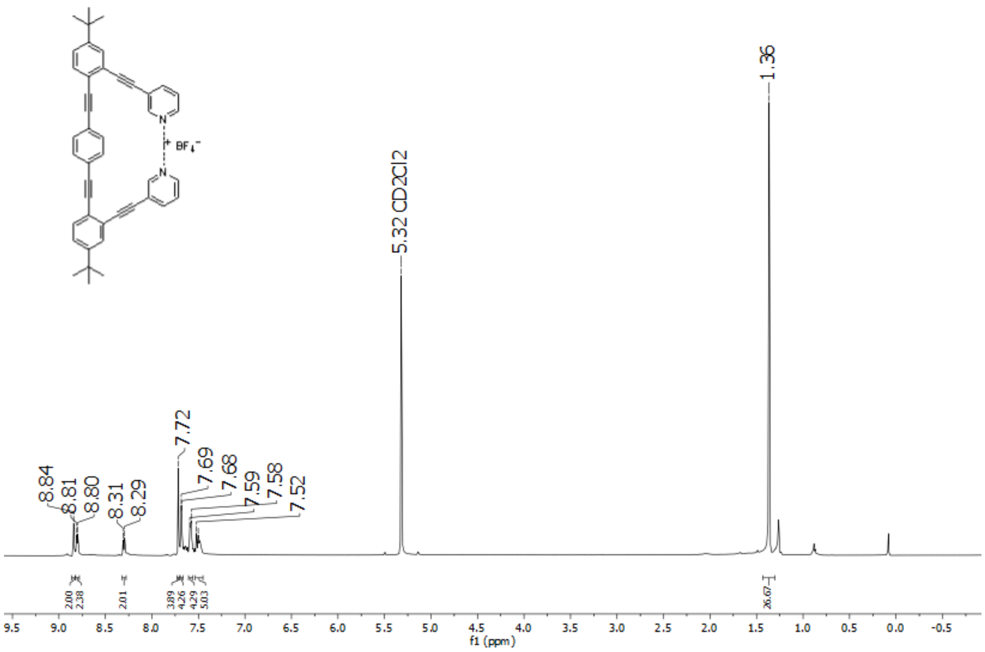

Figure S4. ^{1}H NMR spectrum of **3** (500 MHz, CD_2Cl_2, 25 °C).

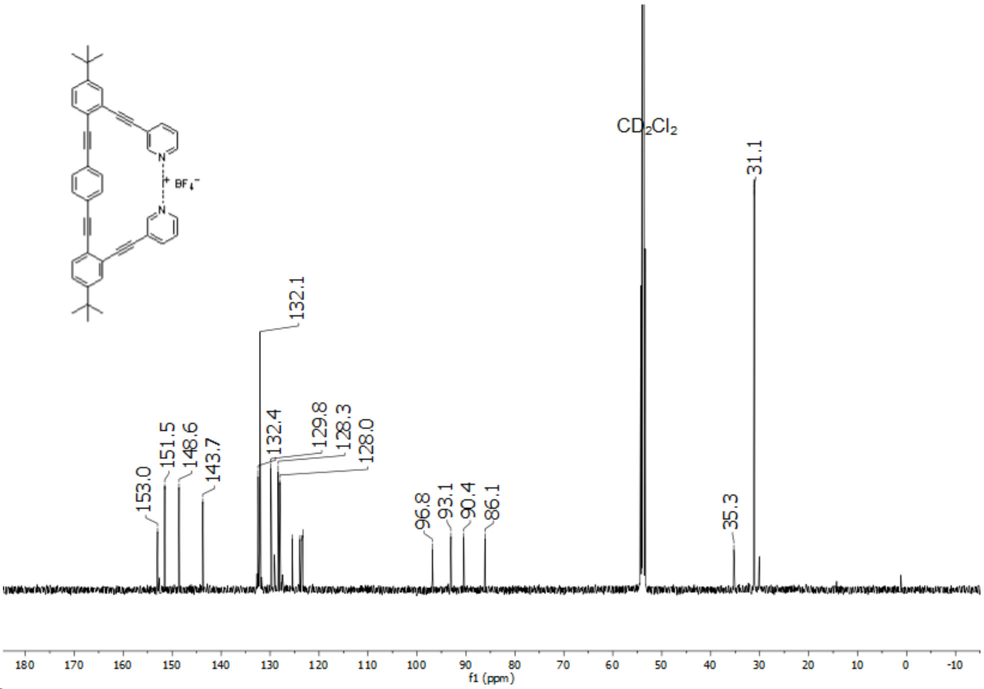

Figure S5. ^{13}C NMR spectrum of **3** (126 MHz, CD_2Cl_2, 25 °C).

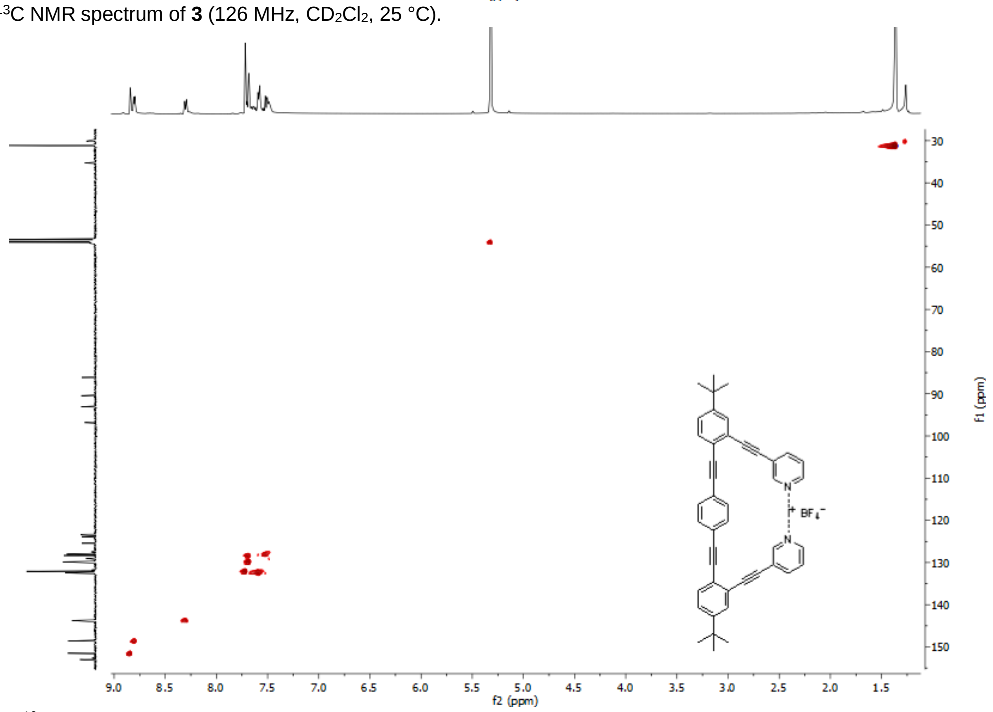

Figure S6. ^{1}H,^{13}C HSQC spectrum of **3** (500 and 126 MHz, CD_2Cl_2, 25 °C).

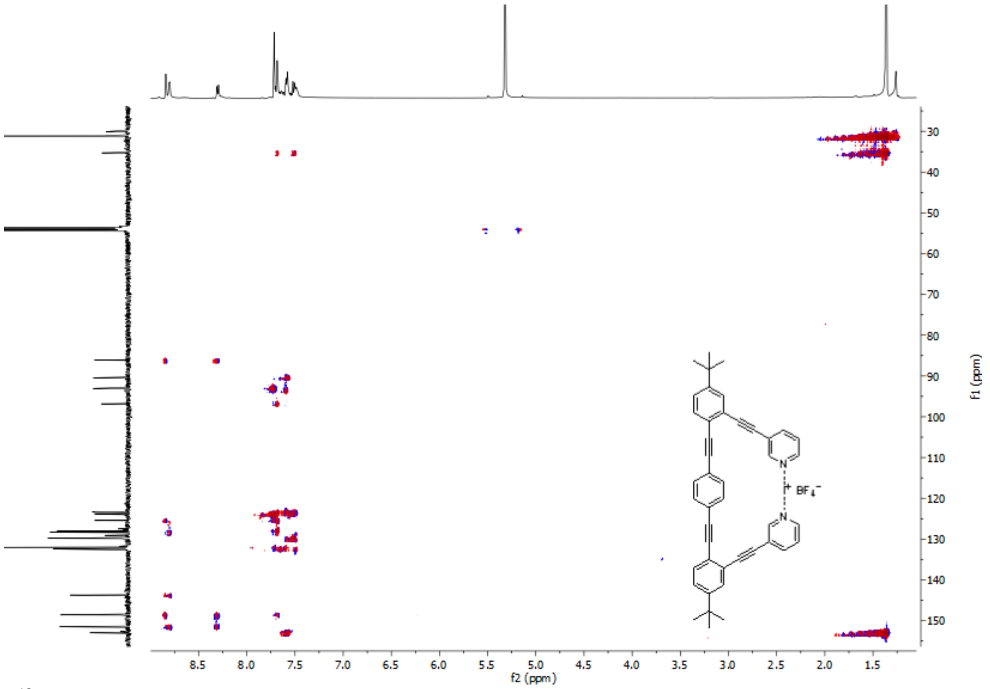

Figure S7. $^{1}H,^{13}C$ HMBC spectrum of **3** (500 and 126 MHz, CD_2Cl_2, 25 °C).

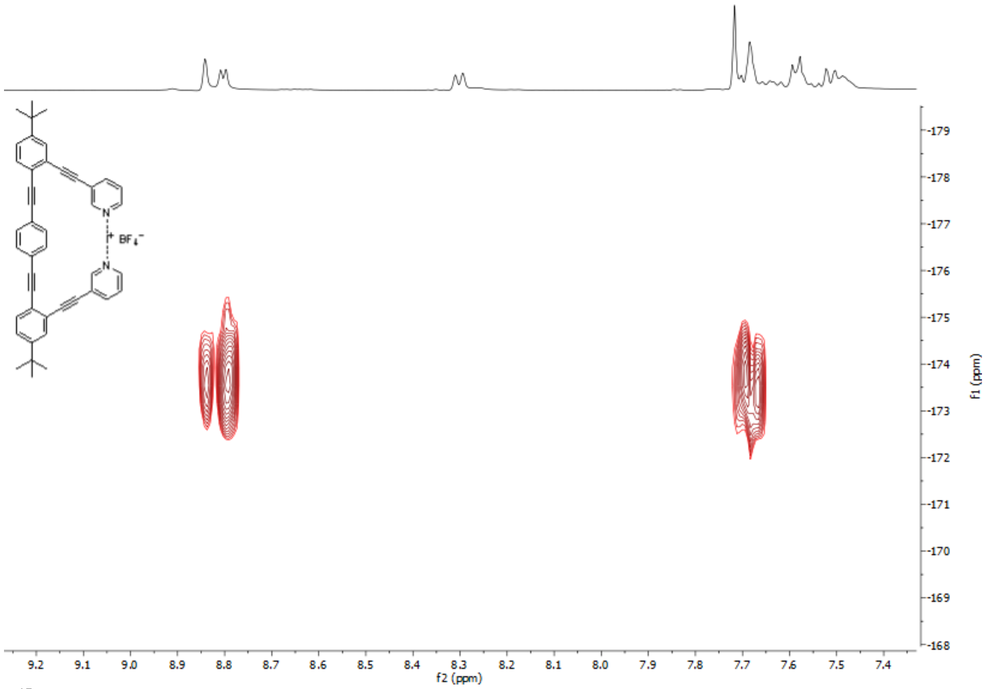

Figure S8. $^{1}H,^{15}N$ HMBC spectrum of **3** (500 and 51 MHz, CD_2Cl_2, 25 °C).

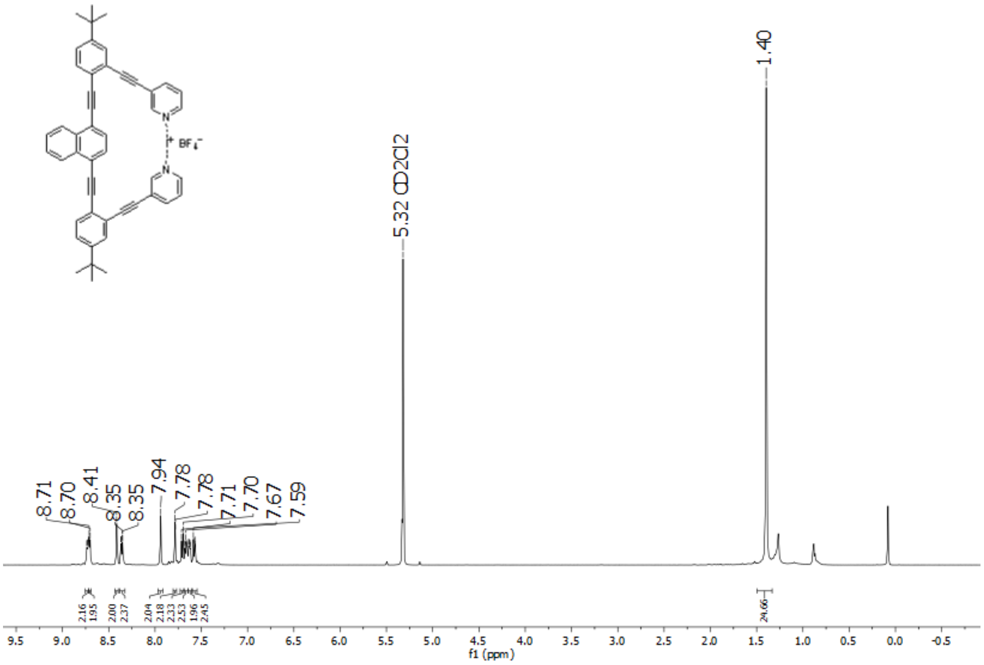

Figure S9. ^{1}H NMR spectrum of **4** (500 MHz, CD_2Cl_2, 25 °C).

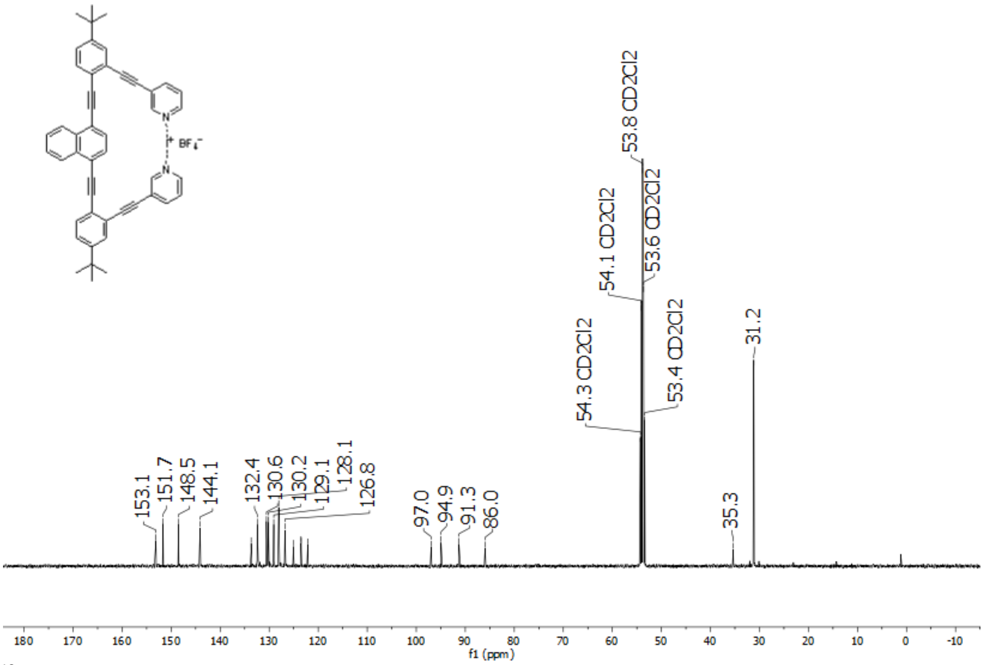

Figure S10. ^{13}C NMR spectrum of **4** (126 MHz, CD_2Cl_2, 25 °C).

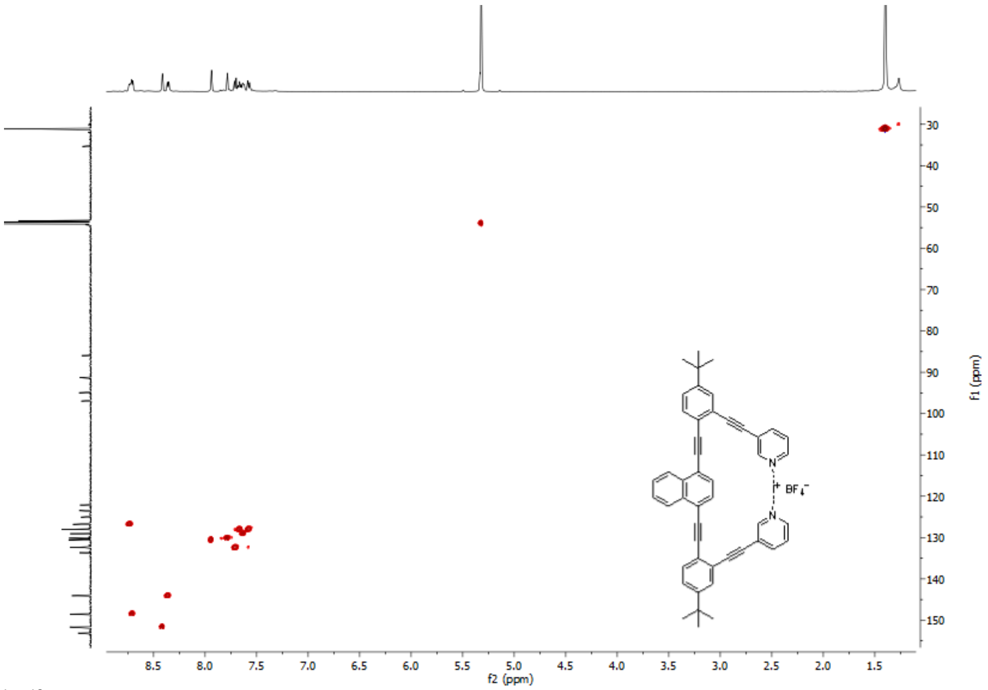

Figure S11. $^{1}H,^{13}C$ HSQC spectrum of **4** (500 and 126 MHz, CD_2Cl_2, 25 °C).

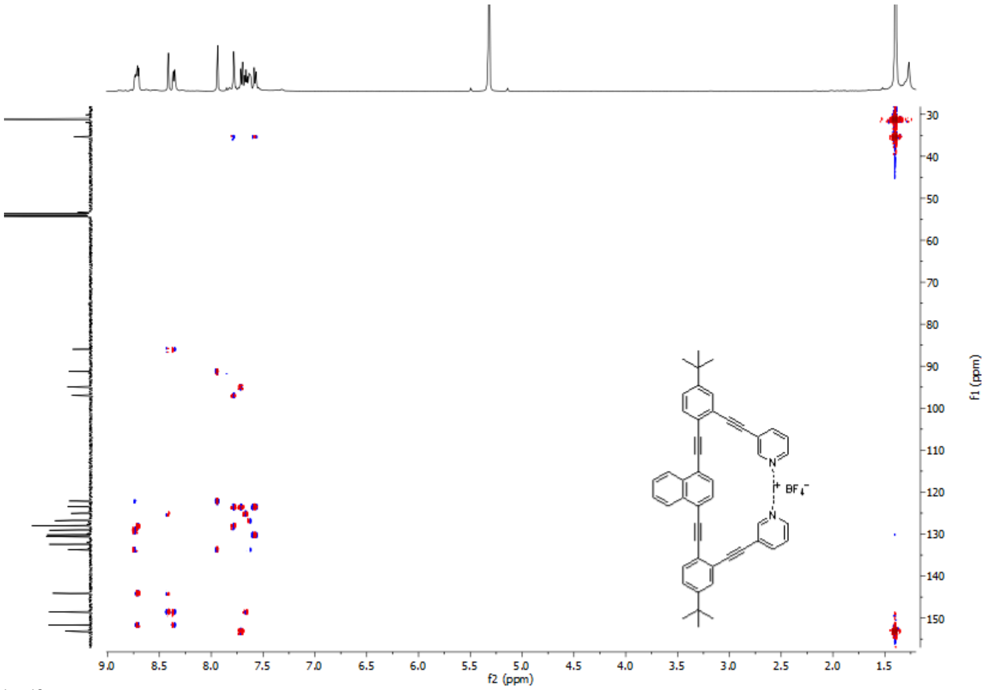

Figure S12. $^{1}H,^{13}C$ HMBC spectrum of **4** (500 and 126 MHz, CD_2Cl_2, 25 °C).

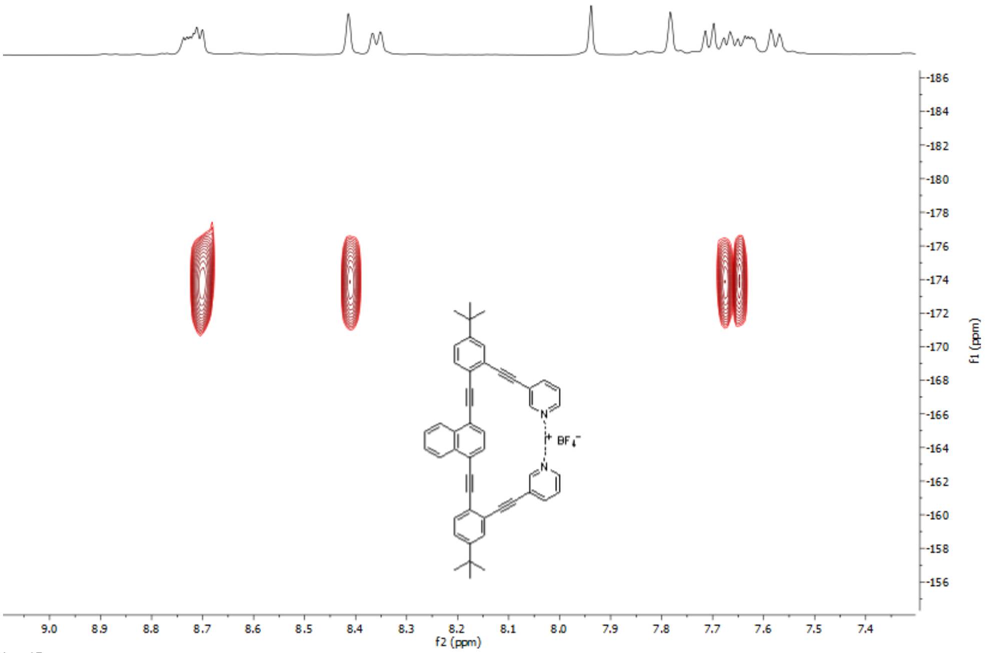

Figure S13. $^{1}H,^{15}N$ HMBC spectrum of **4** (500 and 51 MHz, CD_2Cl_2, 25 °C).

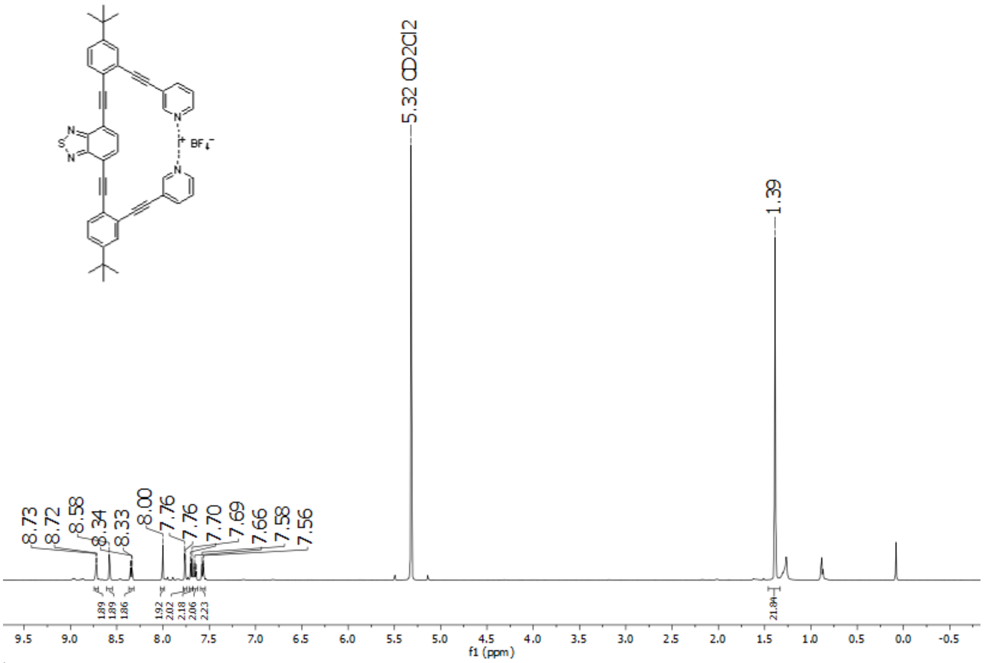

Figure S14. ^{1}H NMR spectrum of **5** (500 MHz, CD_2Cl_2, 25 °C).

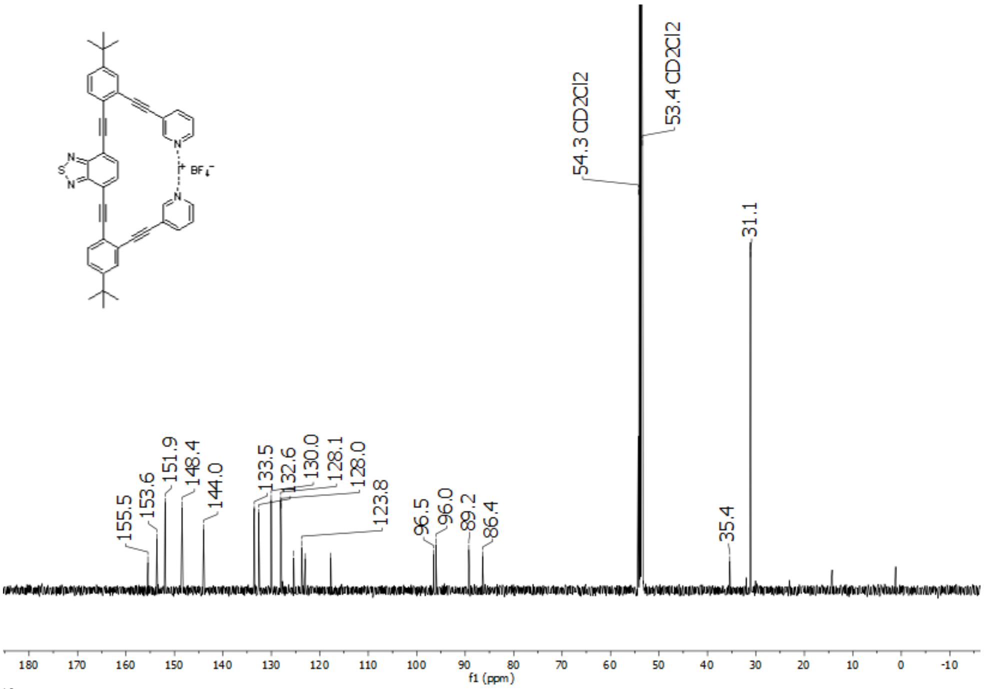

Figure S15. ^{13}C NMR spectrum of **5** (126 MHz, CD_2Cl_2, 25 °C).

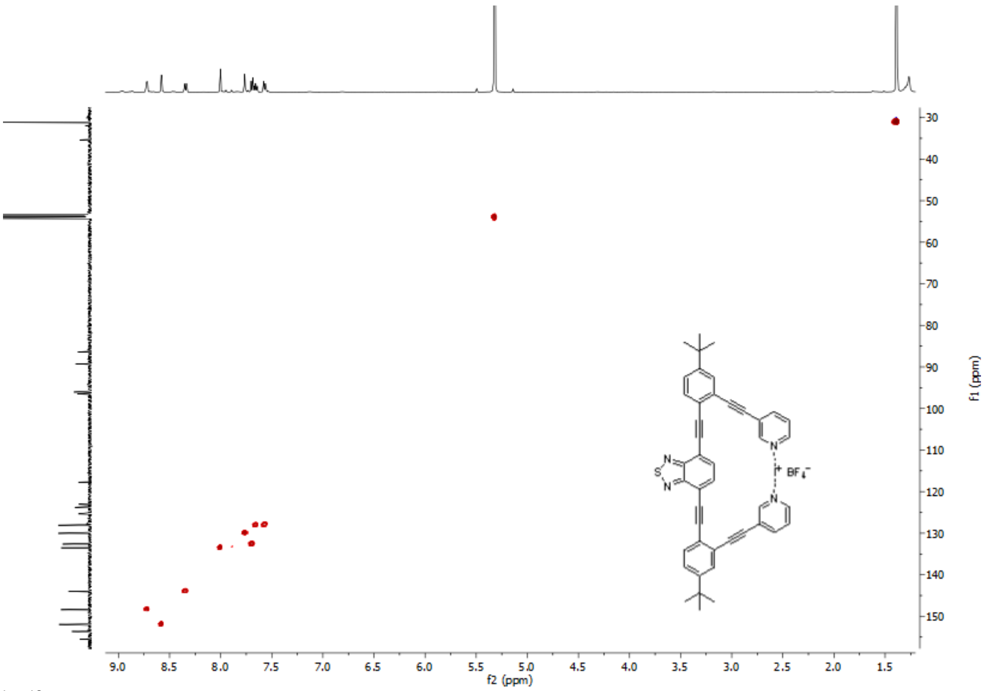

Figure S16. $^{1}H/^{13}C$ HSQC spectrum of **5** (500 and 126 MHz, CD_2Cl_2, 25 °C).

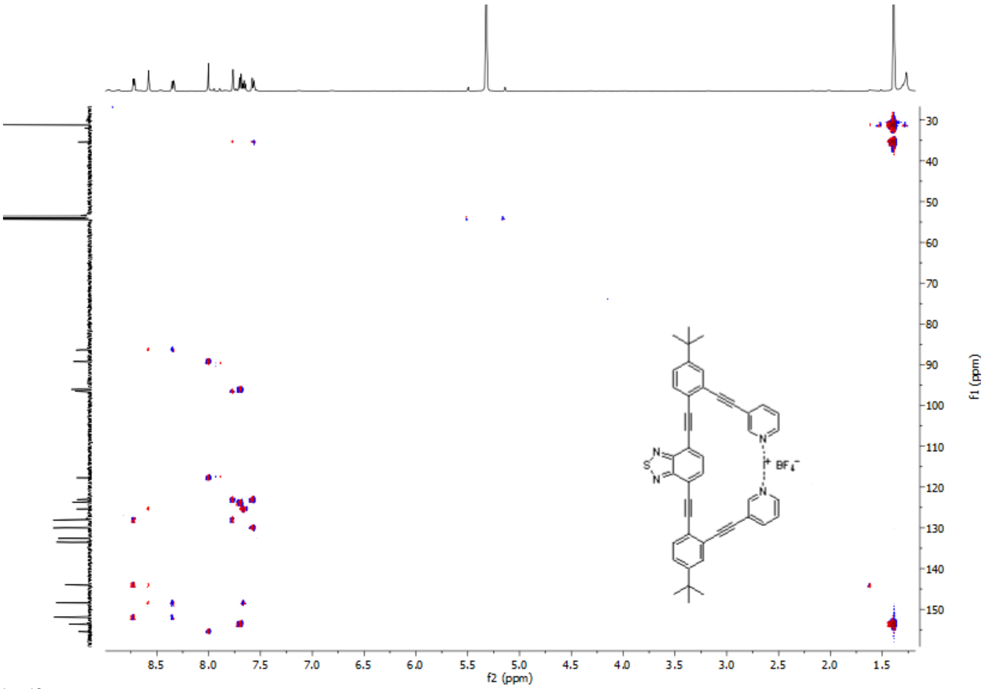

Figure S17. $^{1}H,^{13}C$ HMBC spectrum of **5** (500 and 126 MHz, CD_2Cl_2, 25 °C).

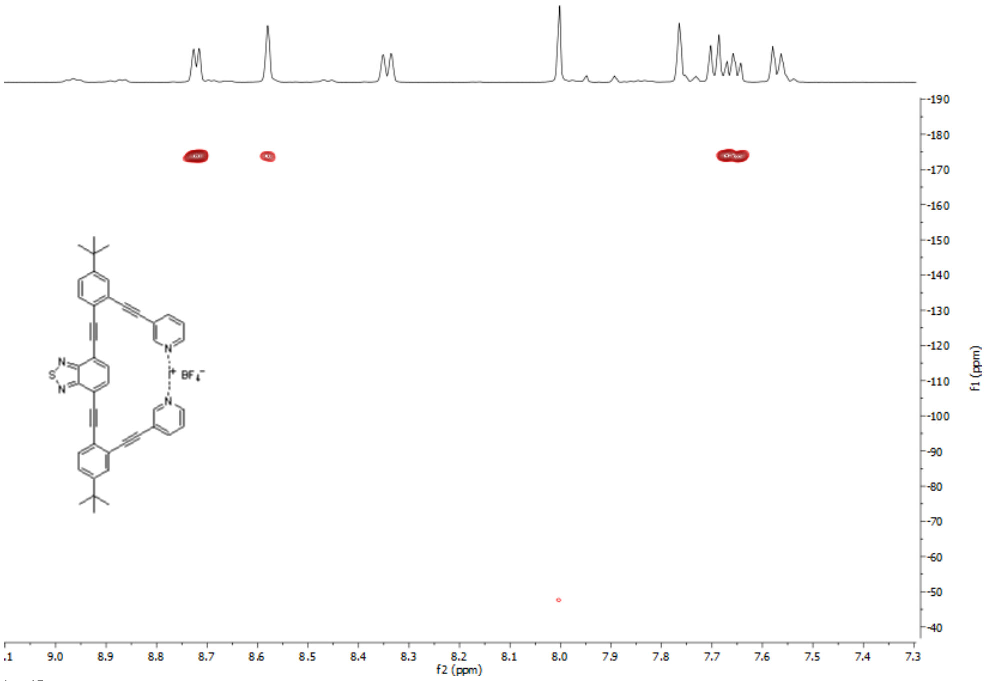

Figure S18. $^{1}H,^{15}N$ HMBC spectrum of **5** (500 and 51 MHz, CD_2Cl_2, 25 °C).

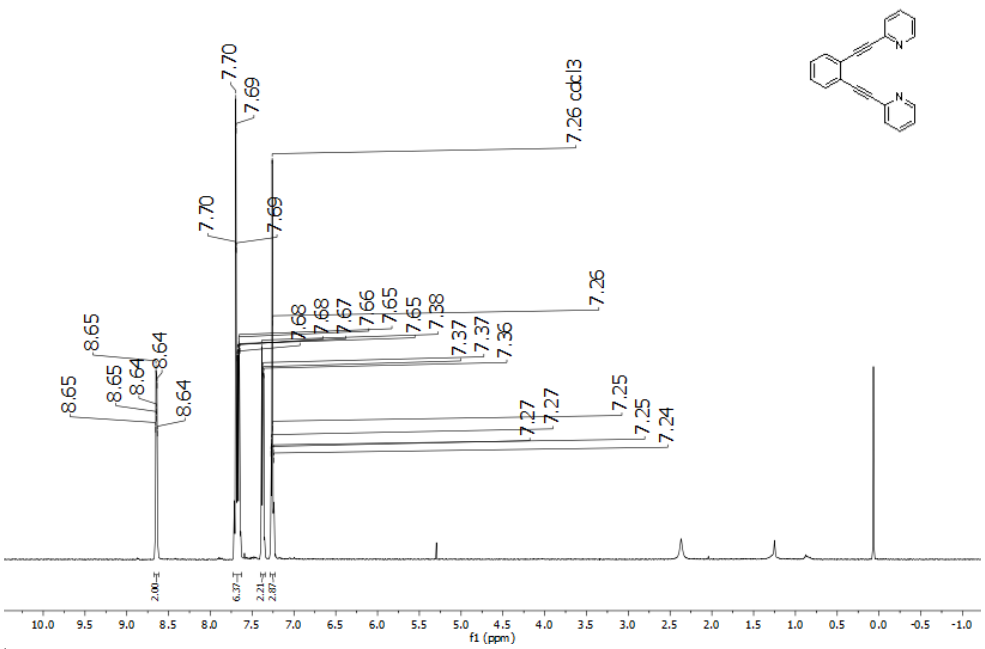

Figure S19. ^{1}H NMR spectrum of **6** (400 MHz, $CDCl_3$, 25 °C).

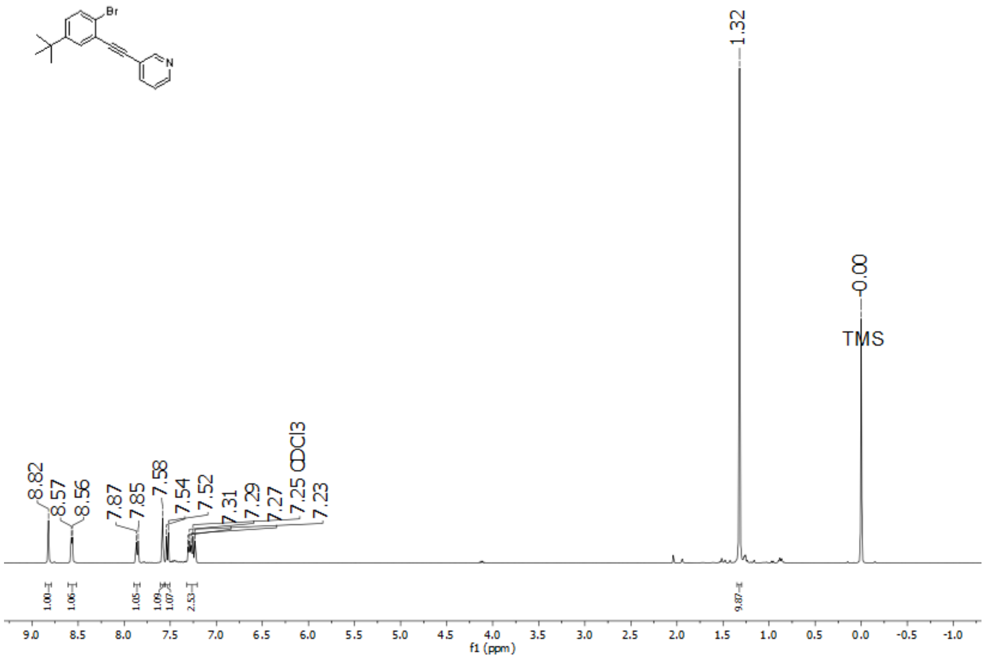

Figure S20. ^{1}H NMR spectrum of **7** (400 MHz, $CDCl_3$, 25 °C).

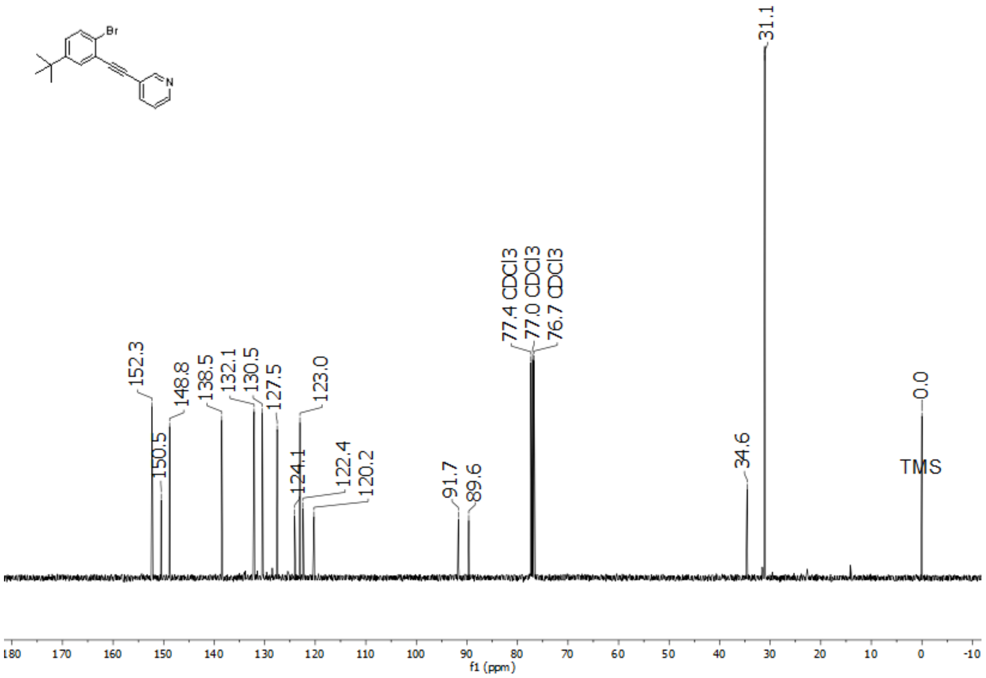

Figure S21. ^{13}C NMR spectrum of **7** (100 MHz, $CDCl_3$, 25 °C).

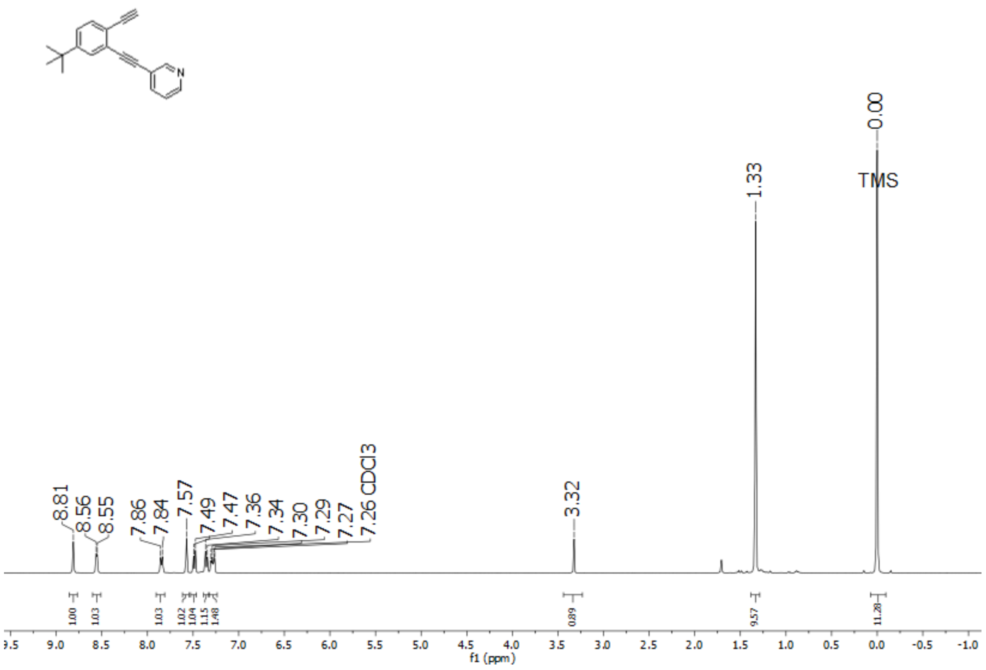

Figure S22. ^{1}H NMR spectrum of **8** (400 MHz, $CDCl_3$, 25 °C).

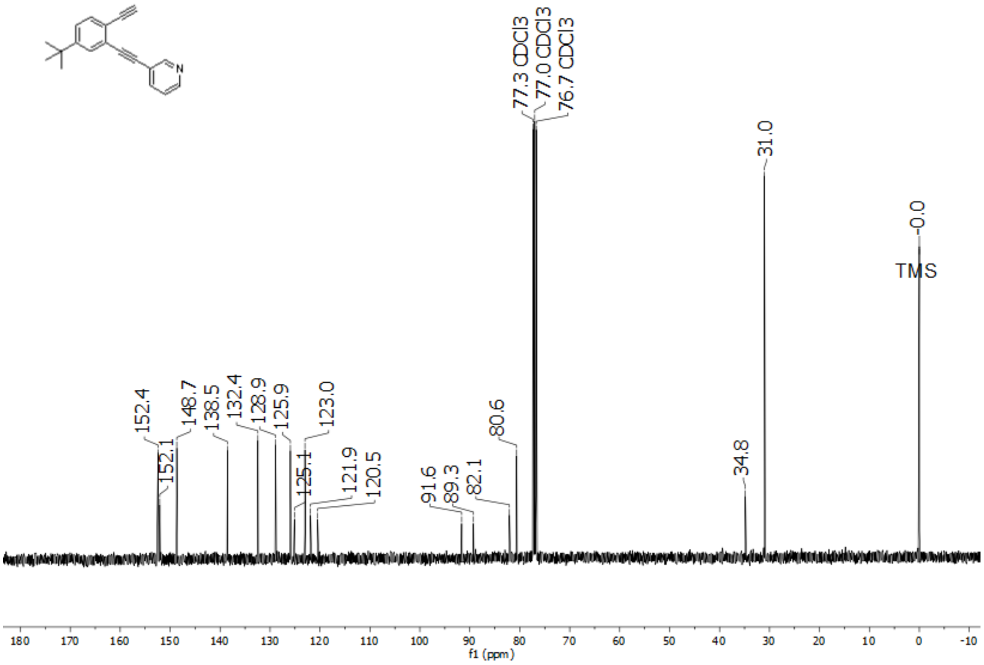

Figure S23. ^{13}C NMR spectrum of **8** (100 MHz, $CDCl_3$, 25 °C).

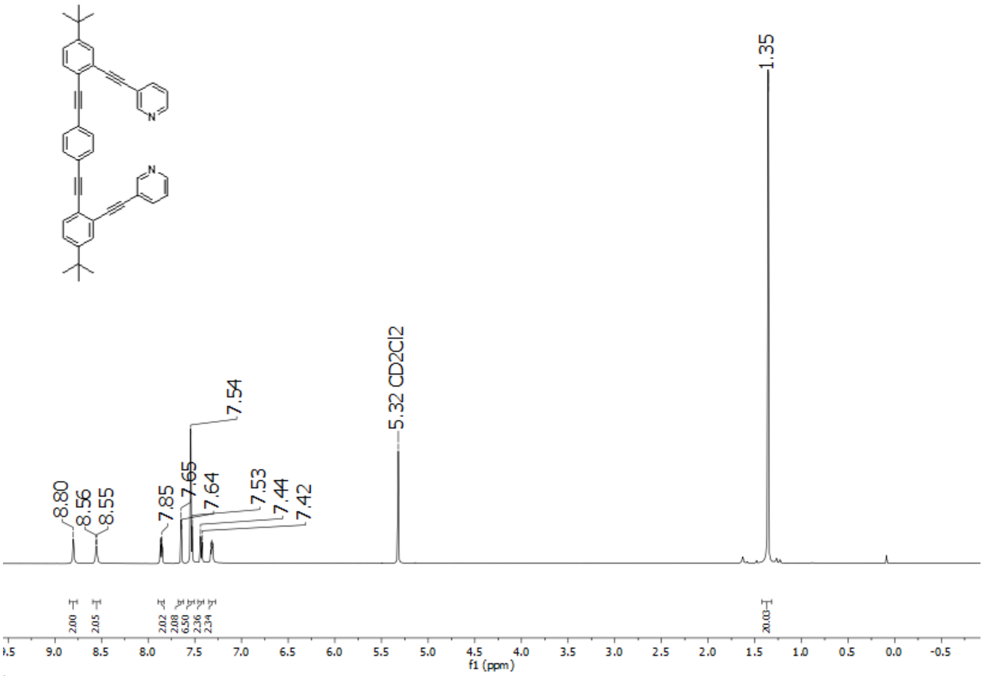

Figure S24. ^{1}H NMR spectrum of **9** (500 MHz, CD_2Cl_2, 25 °C).

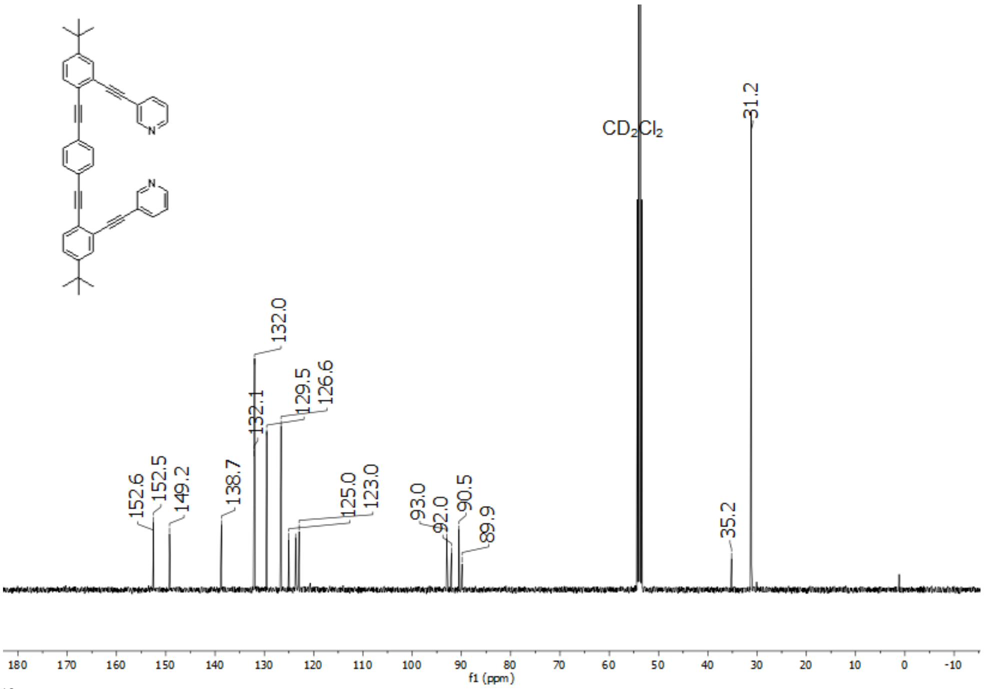

Figure S25. ^{13}C NMR spectrum of **9** (126 MHz, CD_2Cl_2, 25 °C).

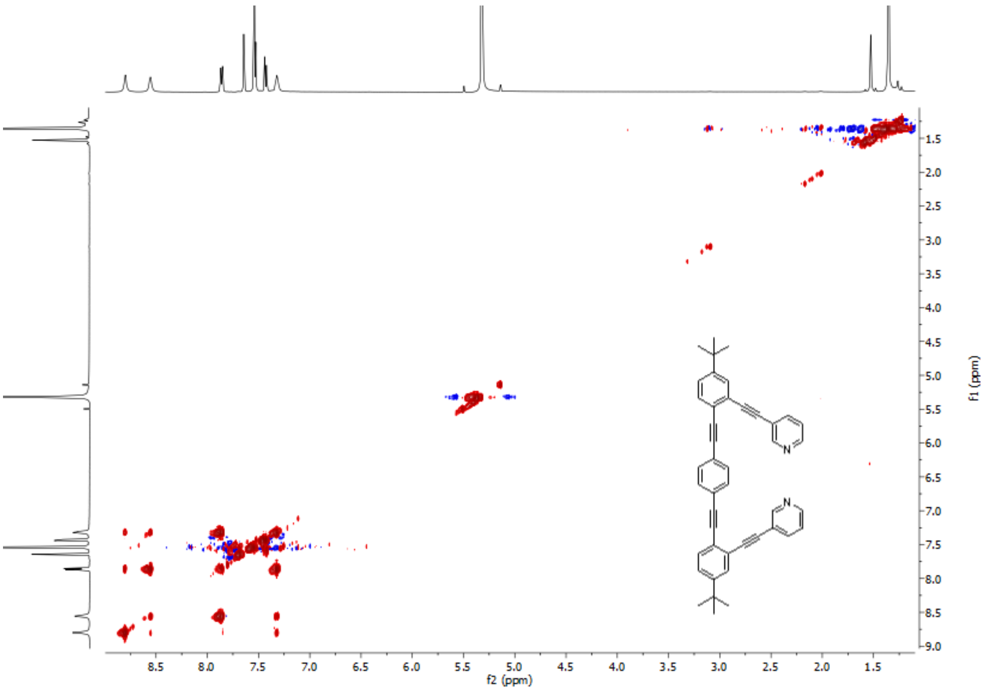

Figure S26. TOCSY spectrum of **9** (500 MHz, CD_2Cl_2, 25 °C).

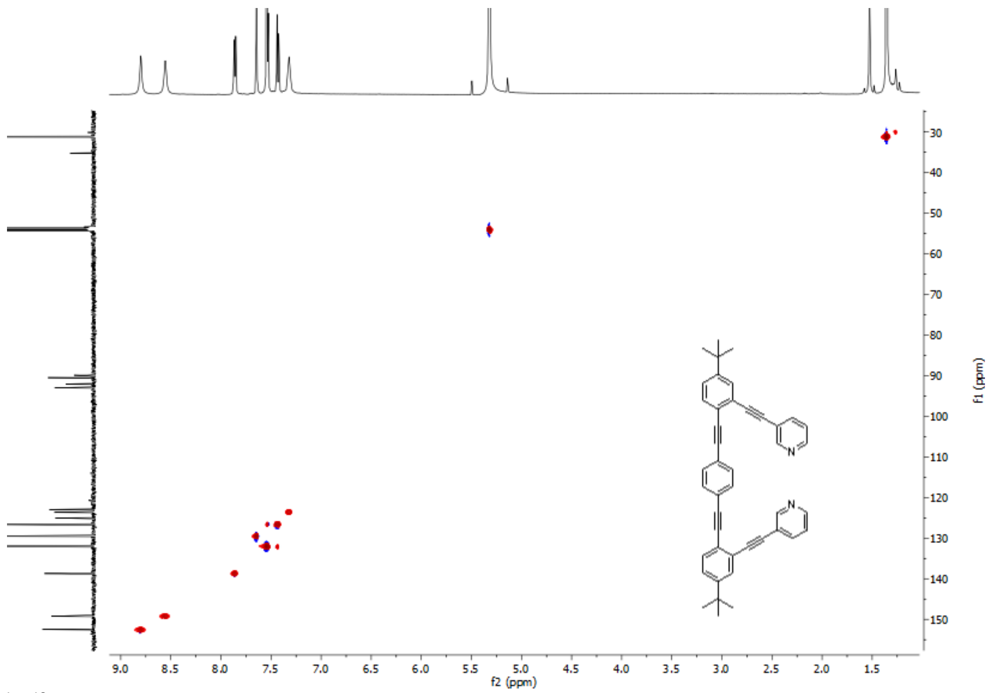

Figure S27. $^{1}H,^{13}C$ HSQC spectrum of **9** (500 and 126 MHz, CD_2Cl_2, 25 °C).

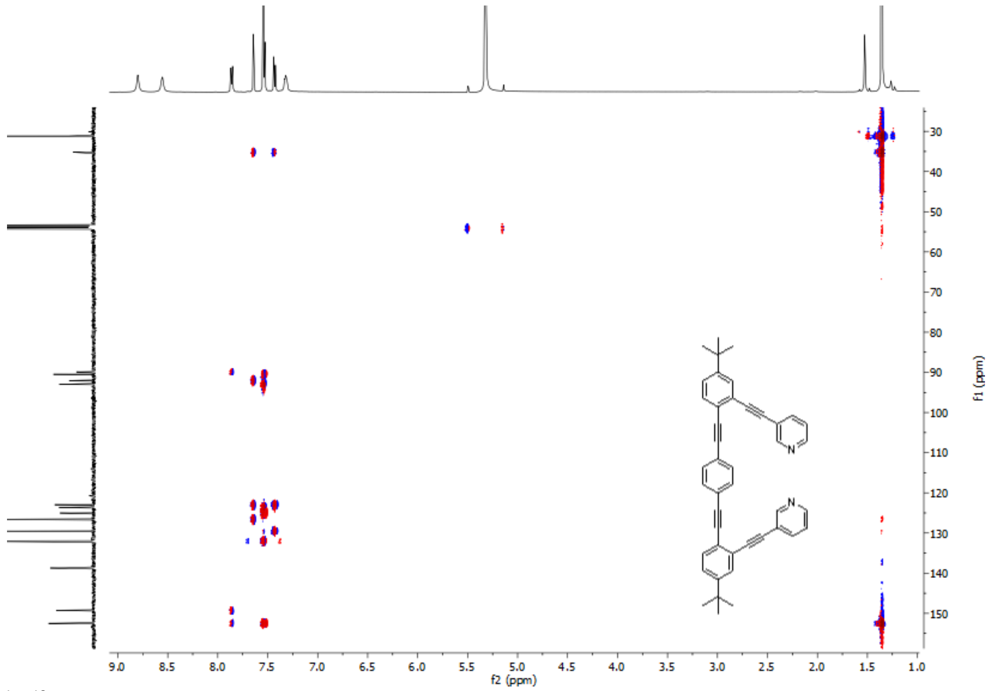

Figure S28. $^{1}H,^{13}C$ HMBC spectrum of **9** (500 and 126 MHz, CD_2Cl_2, 25 °C).

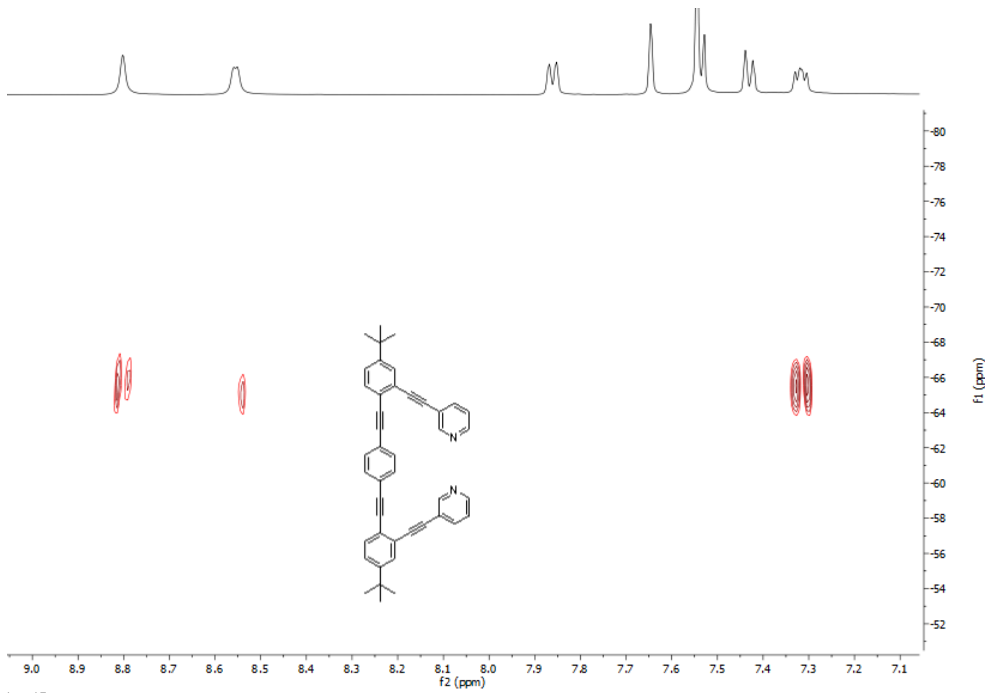

Figure S29. $^{1}H,^{15}N$ HMBC spectrum of **9** (500 and 51 MHz, CD_2Cl_2, 25 °C).

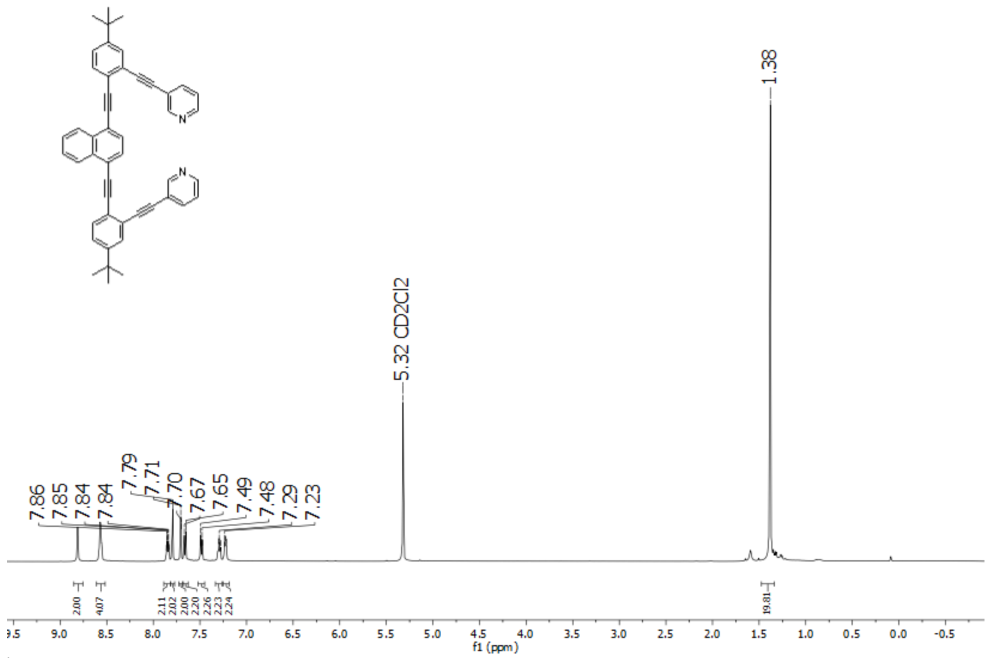

Figure S30. ^{1}H NMR spectrum of **10** (500 MHz, CD_2Cl_2, 25 °C).

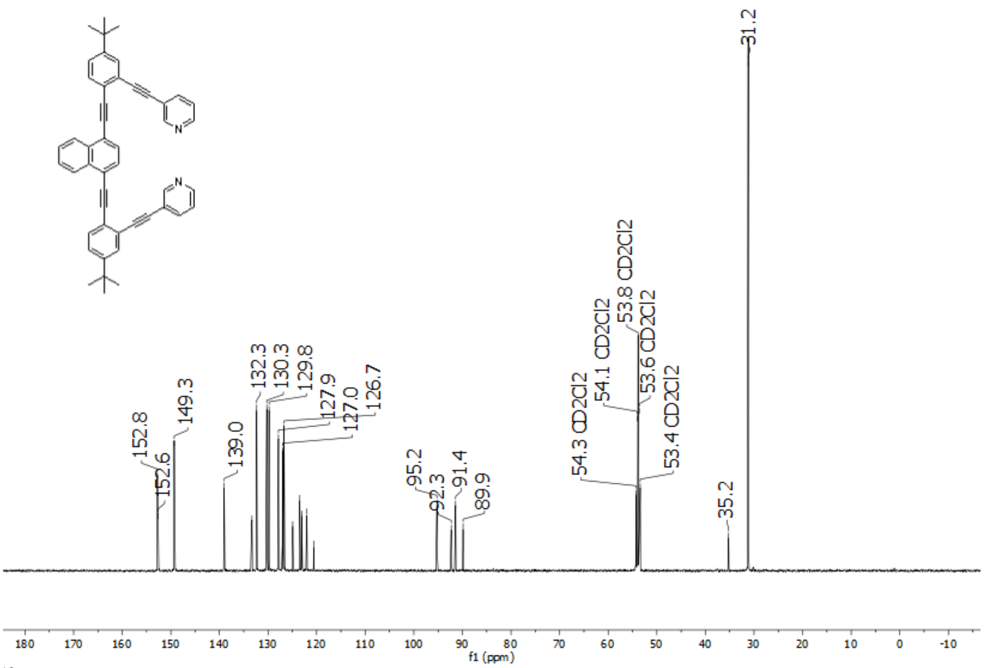

Figure S31. ^{13}C NMR spectrum of **10** (126 MHz, CD_2Cl_2, 25 °C).

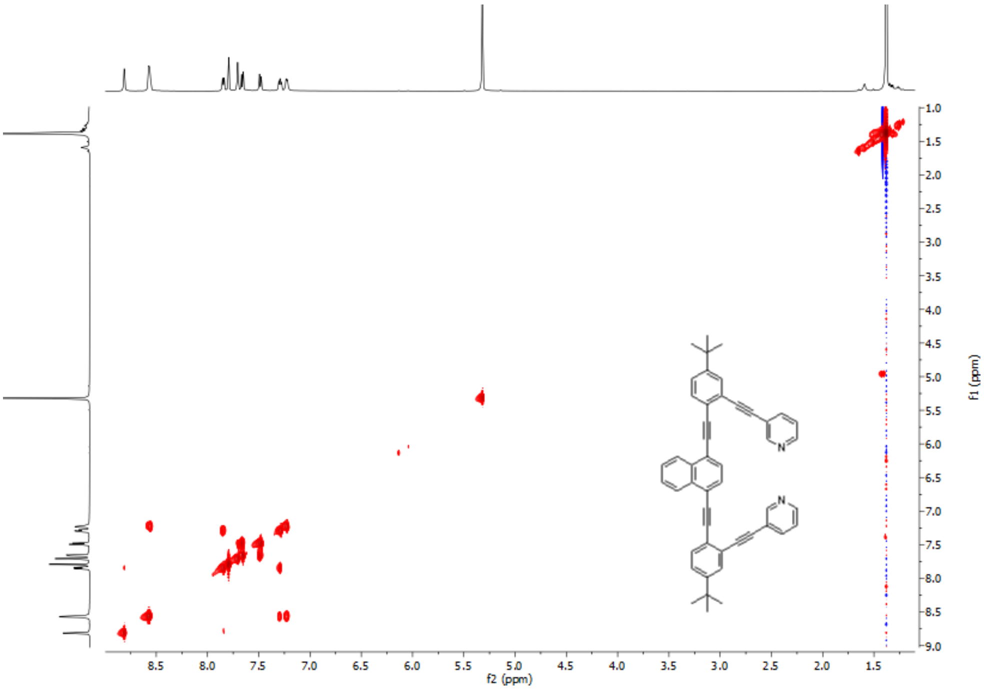

Figure S32. COSY spectrum of **10** (500 MHz, CD_2Cl_2, 25 °C).

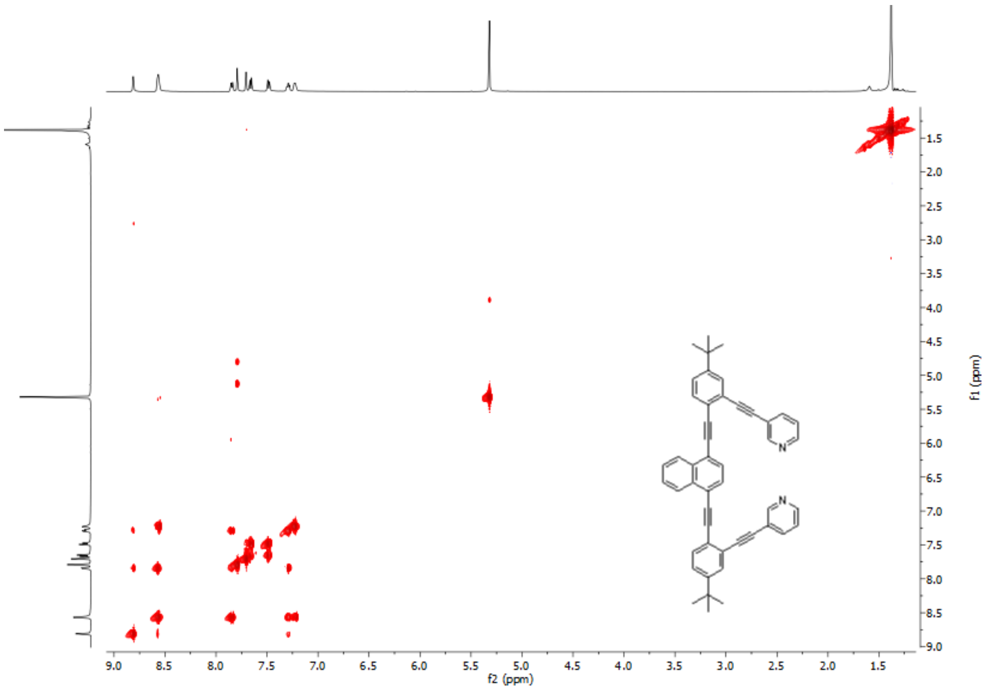

Figure S33. TOCSY spectrum of **10** (500 MHz, CD_2Cl_2, 25 °C).

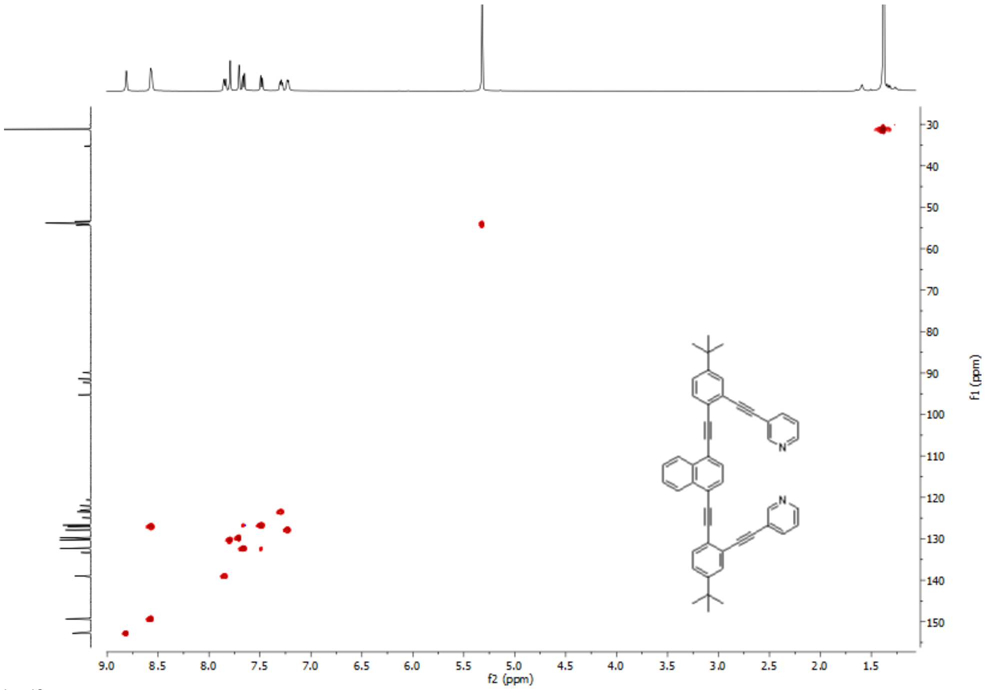

Figure S34. $^1H,^{13}C$ HSQC spectrum of **10** (500 and 126 MHz, CD_2Cl_2, 25 °C).

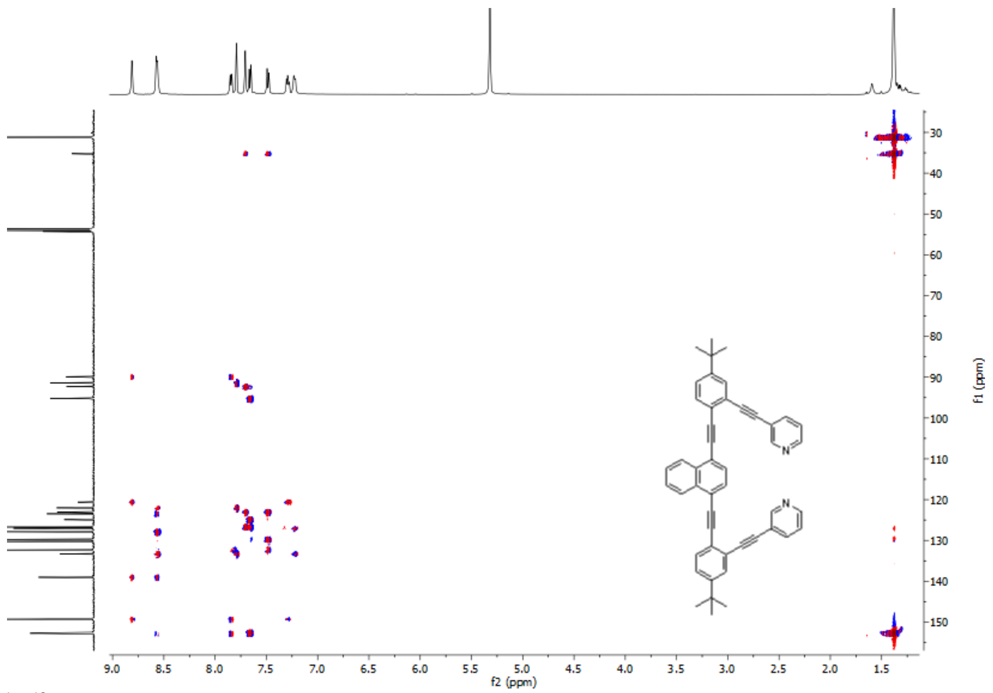

Figure S35. $^{1}H,^{13}C$ HMBC spectrum of **10** (500 and 126 MHz, CD_2Cl_2, 25 °C).

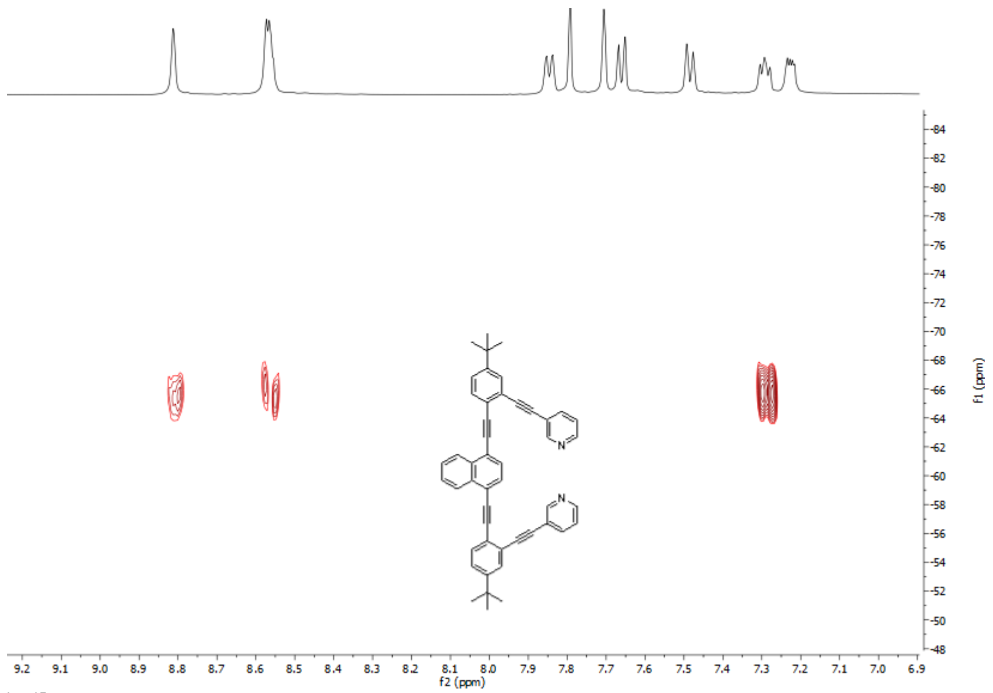

Figure S36. $^{1}H,^{15}N$ HMBC spectrum of **10** (500 and 51 MHz, CD_2Cl_2, 25 °C).

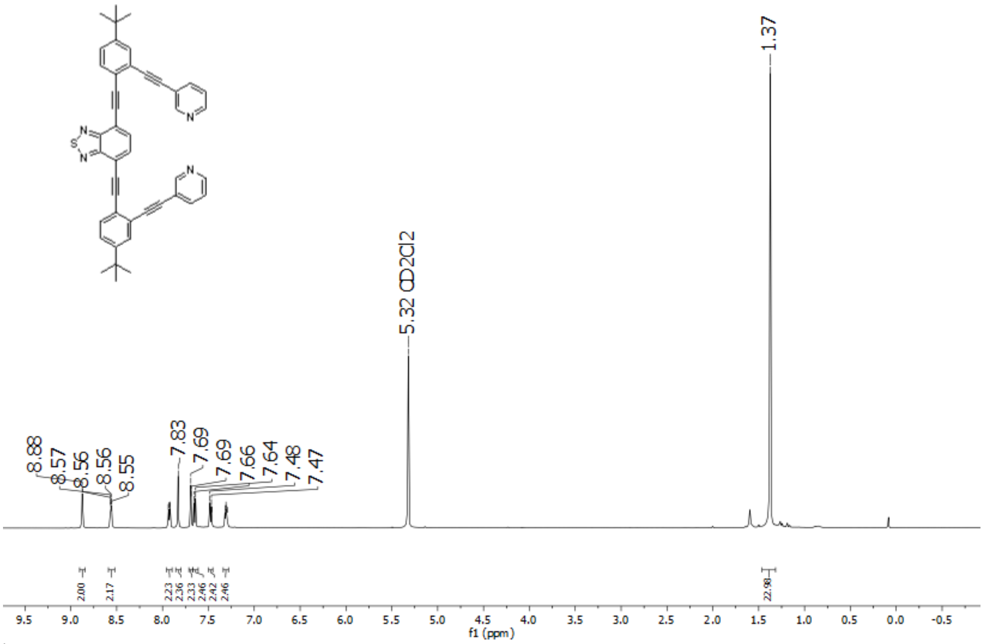

Figure S37. ^{1}H NMR spectrum of **11** (500 MHz, CD_2Cl_2, 25 °C).

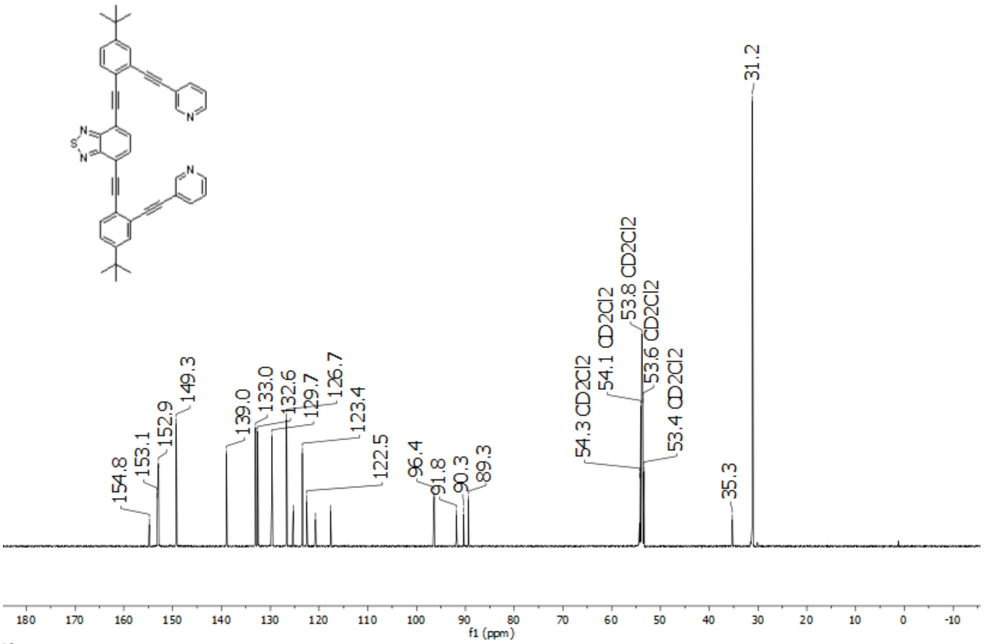

Figure S38. ^{13}C NMR spectrum of **11** (126 MHz, CD_2Cl_2, 25 °C).

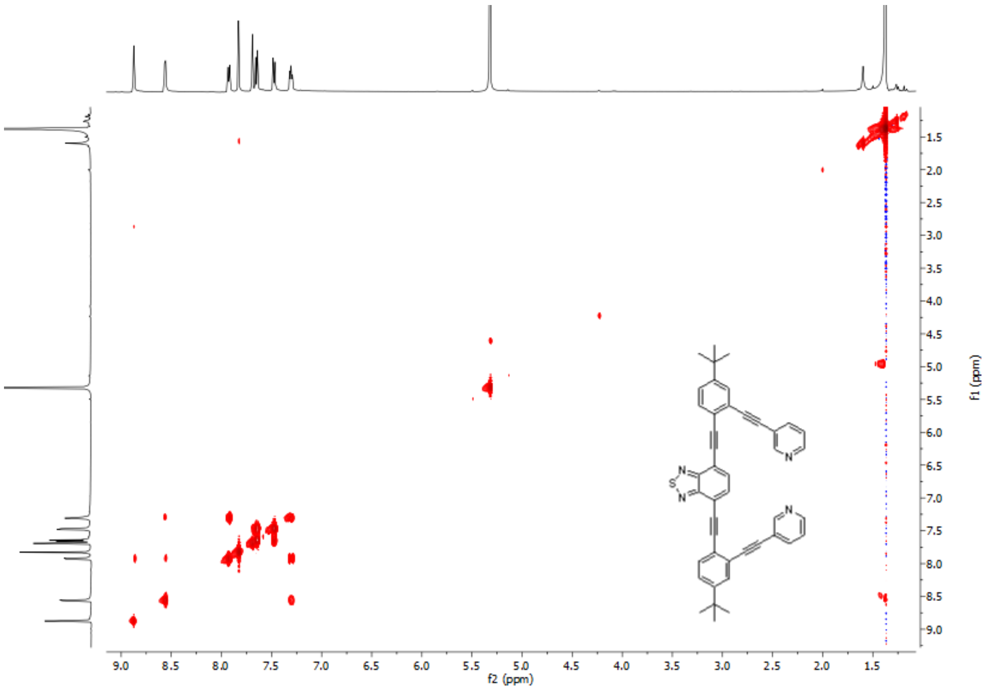

Figure S39. COSY spectrum of **11** (500 MHz, CD_2Cl_2, 25 °C).

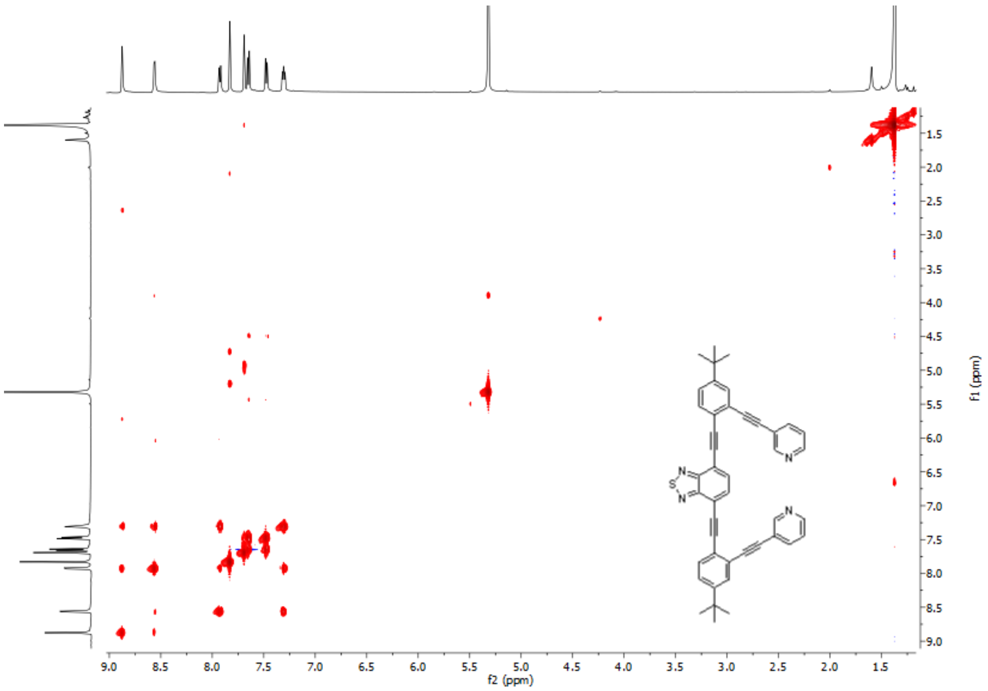

Figure S40. TOCSY spectrum of **11** (500 MHz, CD_2Cl_2, 25 °C).

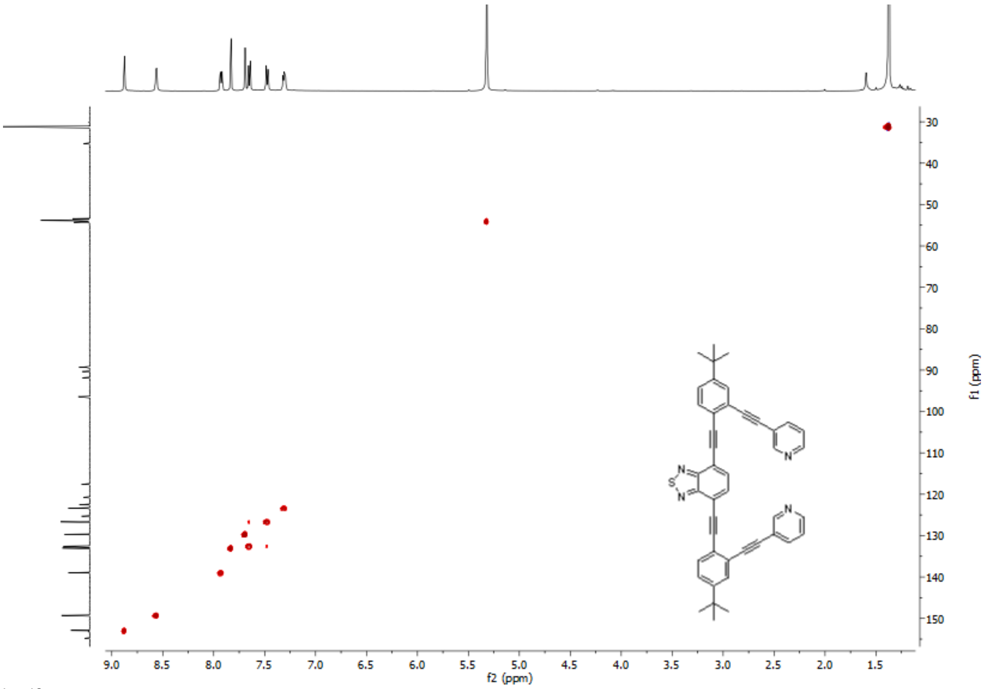

Figure S41. ^{1}H,^{13}C HSQC spectrum of **11** (500 and 126 MHz, CD_2Cl_2, 25 °C).

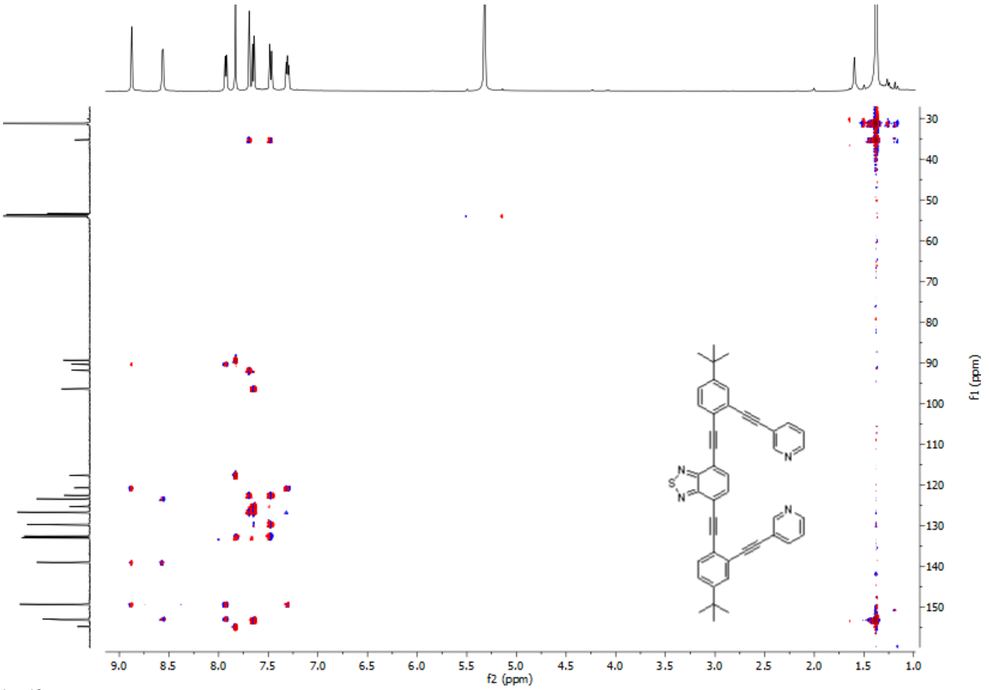

Figure S42. ^{1}H,^{13}C HMBC spectrum of **11** (500 /126 MHz, CD_2Cl_2, 25 °C).

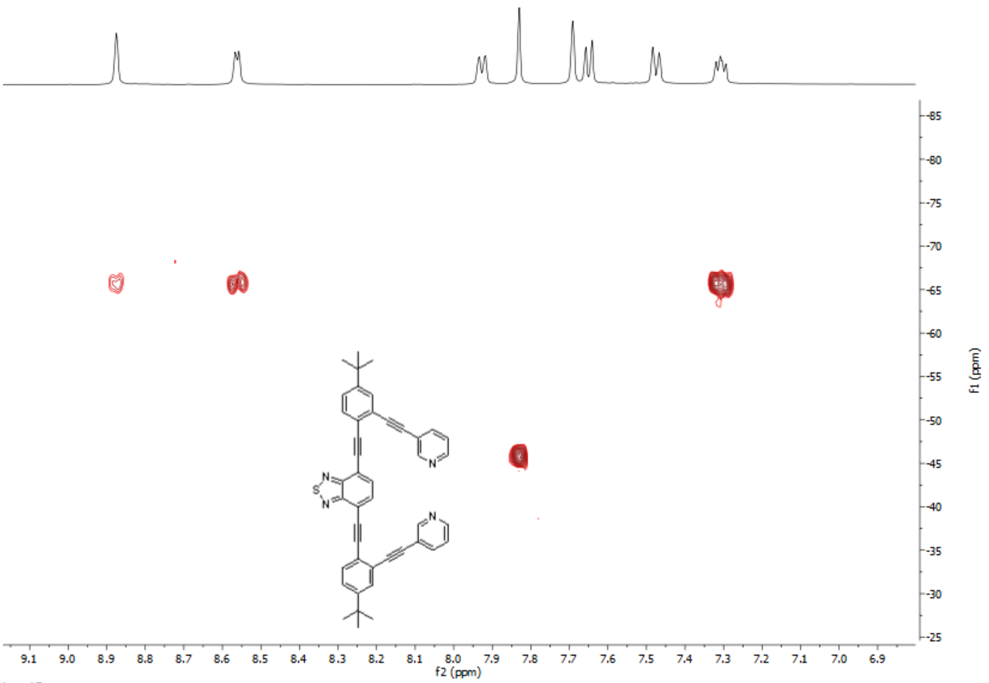

Figure S43. $^{1}H,^{15}N$ HMBC spectrum of **11** (500/51 MHz, CD_2Cl_2, 25 °C).

4. KINETICS

4.1. General information

All kinetic measurements were carried out in anhydrous 1,2-dichloroethane (purchased at Sigma Aldrich) at 25 °C, using a Cary 100 Bio UV-Vis spectrophotometer. Quartz UV-cuvettes were used with 1 mm (for **1**, **3**, and **4**), 2 mm (for **2**) and 10 mm (for **5**) path lengths. The reactions between the iodine(I) complexes and 4-penten-1-ol were followed by measuring the decrease in Absorbance (A, in Absorbance Units (AU)) at specific wavelengths over time. The data was fitted to the standard exponential decay function $A_t = A_\infty + (A_0 - A_\infty)e^{-(K_{obs})t}$ using Origin, expect for the reaction of **2** which had to be fitted to the two-exponential decay function $A_t = A_{\infty^*} + (A_0 - A_\infty)e^{-(K_{obs})t} + (A_{0^*} - A_{\infty^*})e^{-(K_{obs^*})t}$ due to another, much slower, reaction occurring simultaneously. It is believed to be the pyridine nitrogens reacting with the 1,2-dichloroethane, since evidence of such reactions has been found in literature,[7-8] but no experimental proof of this was obtained. The iodine(I) complex concentration was in all cases held constant while [4-penten-1-ol] was varied. Plotting the K_{obs} values against [4-penten-1-ol] in Excel then gave the rate constant k_2 as the slope. For **5** the intercept of the linear fit could not be set to 0.

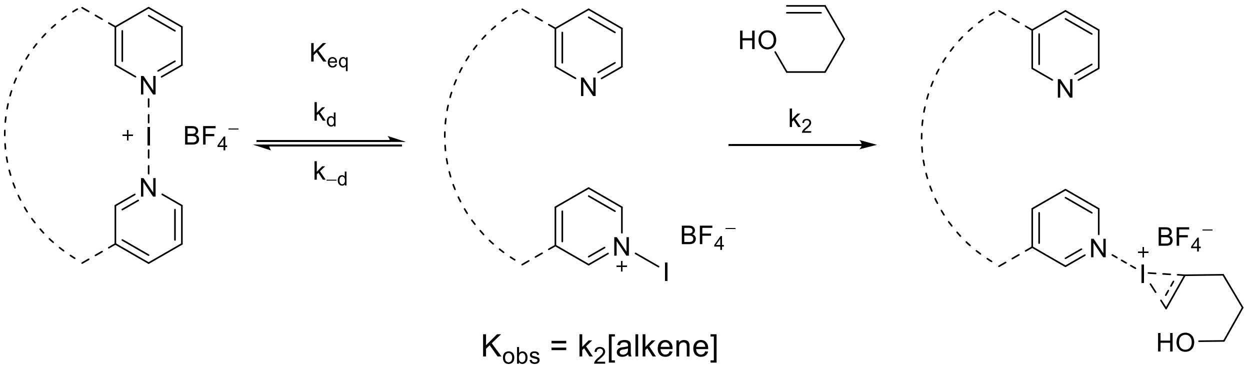

Figure S44. A general, simplified mechanism for the initial steps of the reaction between a pyridine based iodine(I) complex and 4-penten-1-ol, with rate constants.

4.2. Kinetic data

Table S1. The calculated pseudo-first order rate constant K_{obs} (min^{-1}) for the reactions of 4 (0.000040483 M) and 5 (0.000040256 M) with various concentrations of 4-penten-1-ol, as well as the k_2 (M^{-1} min^{-1}) values abstracted from this data. Absorbance was measured at 307.5 nm and 256.5 nm, respectively.

	**1**	**2**
**[4-penten-1-ol] (M)**	**K_{obs} (min^{-1})**	**K_{obs} (min^{-1})**
0.007746	5.578178167	0.03424586
0.015493	5.904581956	0.067288729
0.030986	8.45094228	0.127023161
0.046479	8.371002846	0.201920262
0.061971	10.24800164	0.281301865
0.077464	10.83188908	0.327693724
**k_2 (M^{-1} min^{-1})**	77.596	4.3318

Table S2. The calculated pseudo-first order rate constant K_{obs} (min^{-1}) for the reactions of 1 (0.000040298 M), 2 (0.000040862 M), and 3 (0.000040483 M) with various concentrations of 4-penten-1-ol, as well as the k_2 (M^{-1} min^{-1}) values abstracted from this data. Absorbance was measured at 298 nm, 316 nm, and 295 nm, respectively.

	**3**	**4**	**5**
**[4-penten-1-ol] (M)**	**K_{obs} (min^{-1})**	**K_{obs} (min^{-1})**	**K_{obs} (min^{-1})**
0.007746	0.012029	0.010123	0.014130641
0.015493	0.020188	0.019355	0.028347202
0.030986	0.046274	0.038809	0.070292692
0.046479	0.070932	0.047333	0.080715071
0.061971	0.078187	0.080202	0.149733325
0.077464	0.104566	0.089375	0.183103904
**k_2 (M^{-1} min^{-1})**	1.3635	1.1819	2.2578

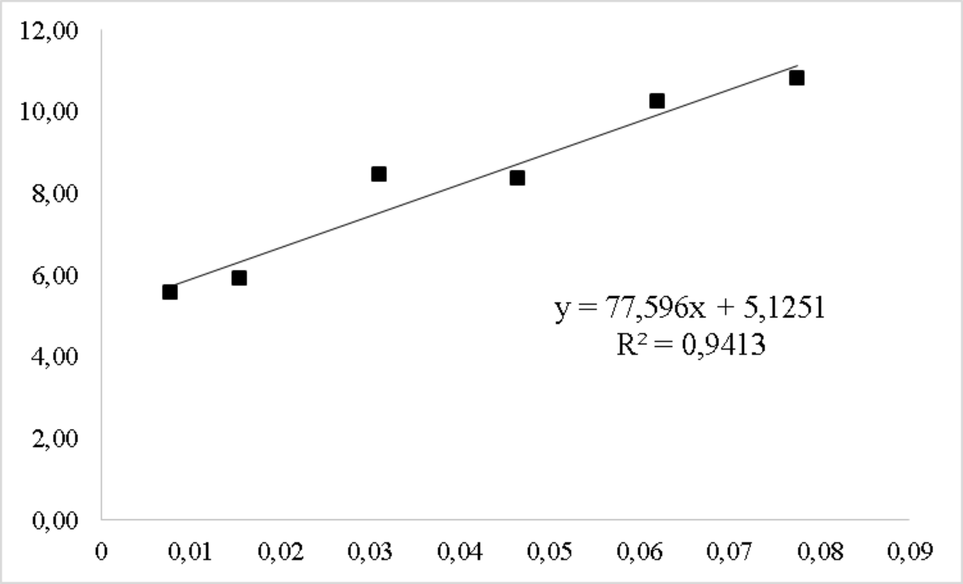

Figure S45. A plot of K_{obs} (min^{-1}) vs [4-penten-1-ol] (M) for **1** (0.000040256 M), measured at 256.5 nm.

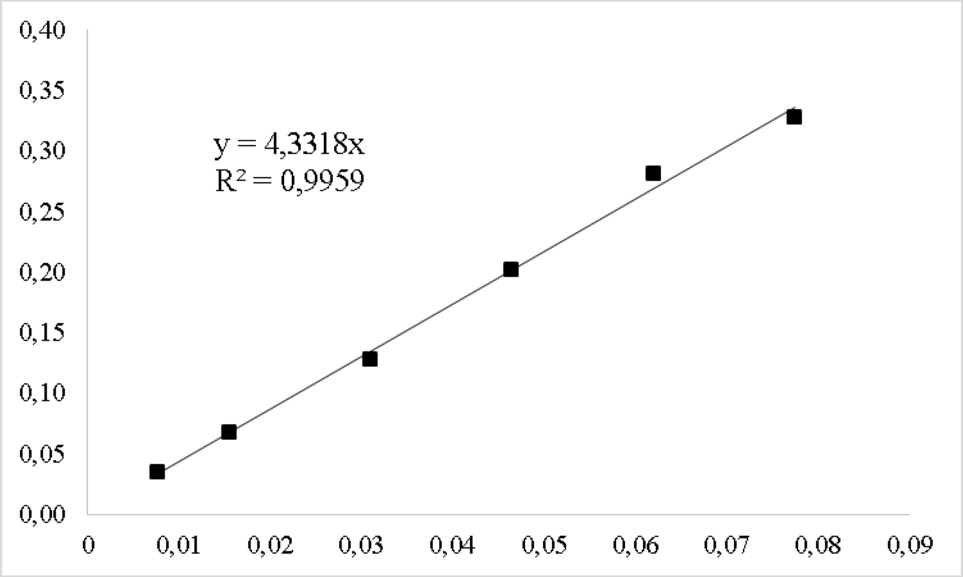

Figure S46. A plot of K_{obs} (min^{-1}) vs [4-penten-1-ol] (M) for **2** (0.000040483 M), measured at 307.5 nm.

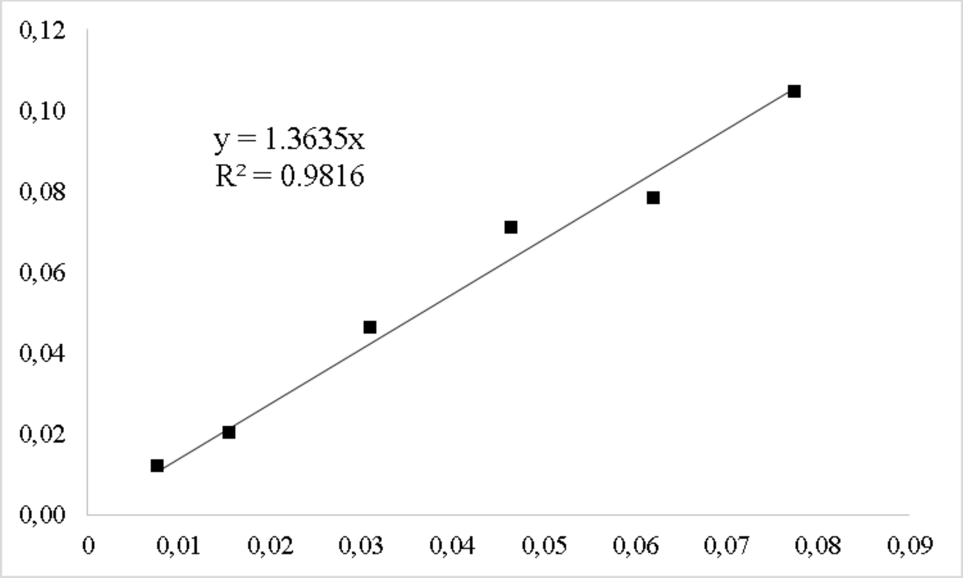

Figure S47. A plot of K_{obs} (min^{-1}) vs [4-penten-1-ol] (M) for **3** (0.000040298 M), measured at 298 nm.

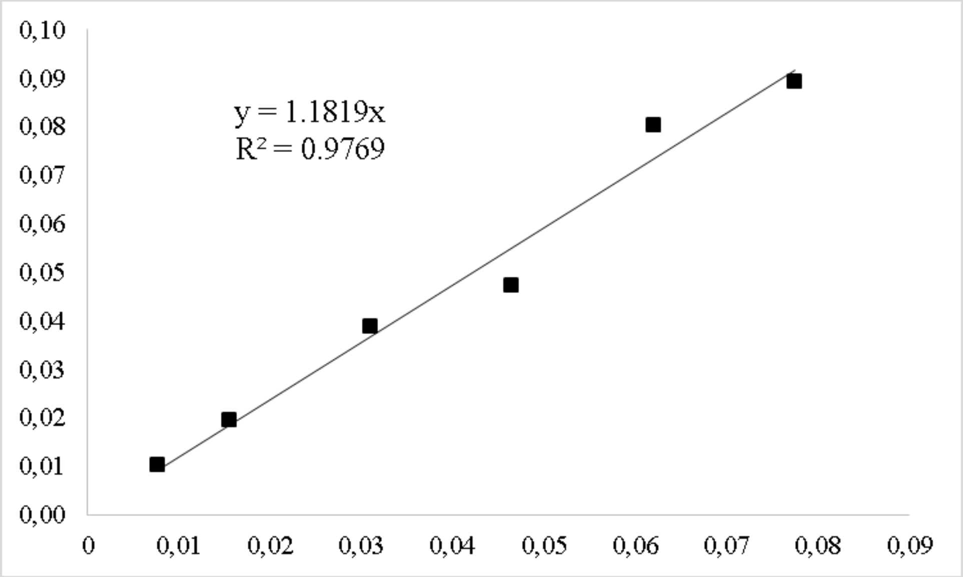

Figure S48. A plot of K_{obs} (min^{-1}) vs [4-penten-1-ol] (M) for **4** (0.000040862 M), measured at 316 nm.

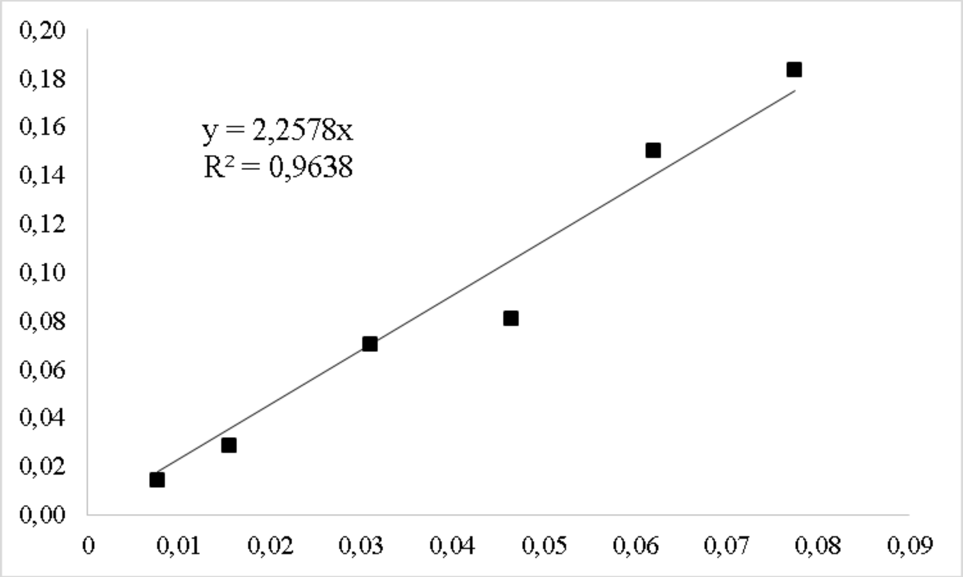

Figure S49. A plot of K_{obs} (min^{-1}) vs [4-penten-1-ol] (M) for **5** (0.000040483 M), measured at 295 nm.

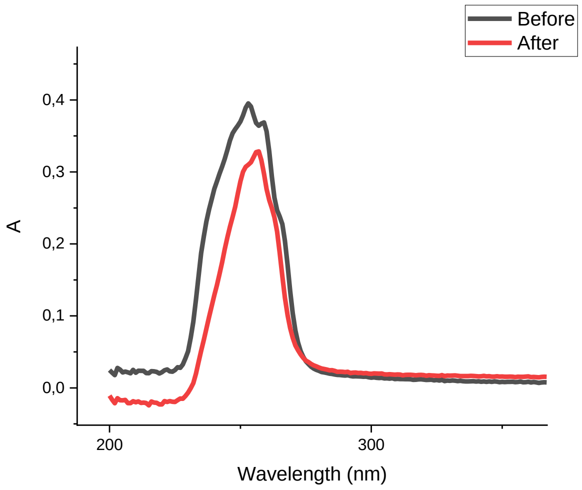

Figure S50. Absorbance (A, in Absorbance Units (AU)) vs wavelength plotted for **1** (0.000040256 M), before and after reaction with 4-penten-1-ol.

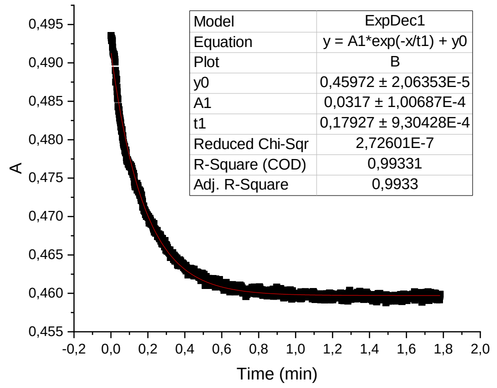

Figure S51. Absorbance (A, in Absorbance Units (AU)) vs time (min) plotted for the reaction of **1** (0.000040256 M) with 4-penten-1-ol (0.007746 M), measured at 256.5 nm. Here, $k_1 = 1/t_1 = 5.578\ min^{-1}$, see Table S1.

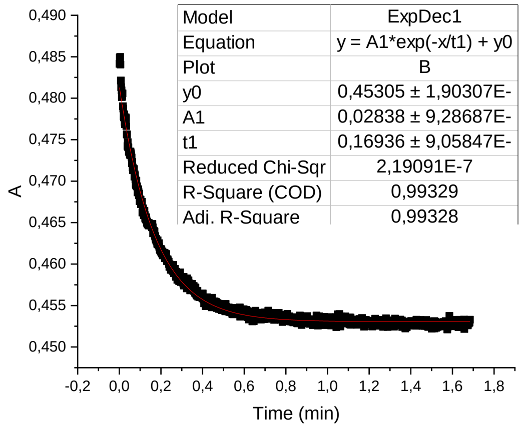

Figure S52. Absorbance (A, in Absorbance Units (AU)) vs time (min) plotted for the reaction of **1** (0.000040256 M) with 4-penten-1-ol (0.015493 M), measured at 256.5 nm. Here, $k_1 = 1/t_1 = 5.905\ min^{-1}$, see Table S1.

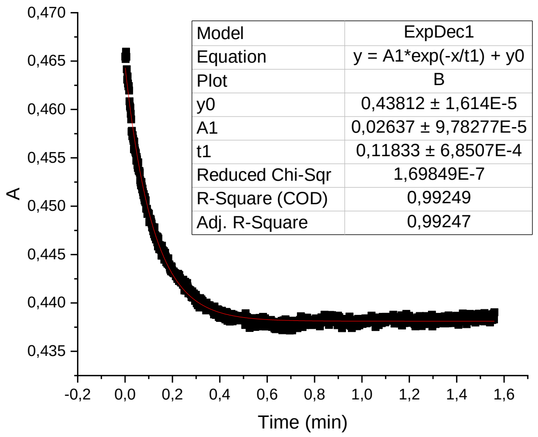

Figure S53. Absorbance (A, in Absorbance Units (AU)) vs time (min) plotted for the reaction of **1** (0.000040256 M) with 4-penten-1-ol (0.030986 M), measured at 256.5 nm. Here, $k_1 = 1/t_1 = 8.451\ min^{-1}$, see Table S1.

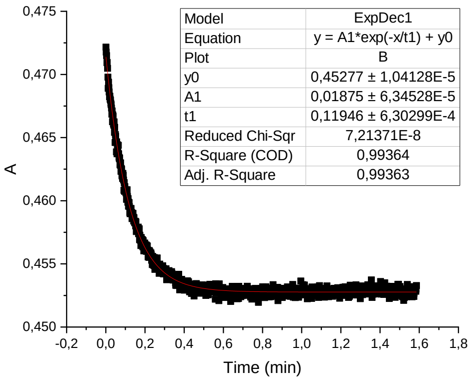

Figure S54. Absorbance (A, in Absorbance Units (AU)) vs time (min) plotted for the reaction of **1** (0.000040256 M) with 4-penten-1-ol (0.046479 M), measured at 256.5 nm. Here, $k_1 = 1/t_1 = 8.371\ min^{-1}$, see Table S1.

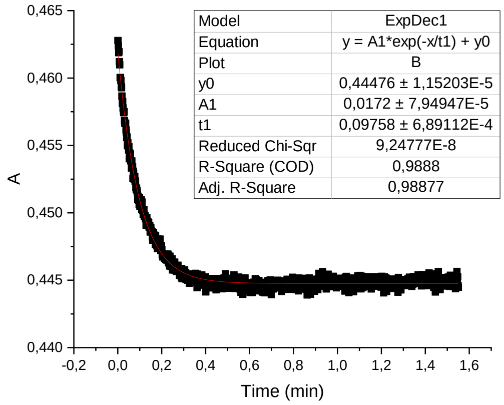

Figure S55. Absorbance (A, in Absorbance Units (AU)) vs time (min) plotted for the reaction of **1** (0.000040256 M) with 4-penten-1-ol (0.061971 M), measured at 256.5 nm. Here, $k_1 = 1/t_1 = 10.248\ min^{-1}$, see Table S1.

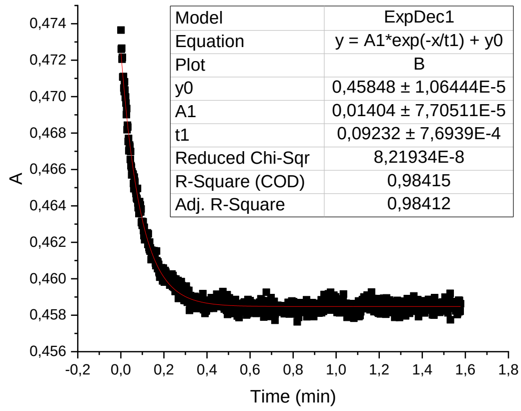

Figure S56. Absorbance (A, in Absorbance Units (AU)) vs time (min) plotted for the reaction of **1** (0.000040256 M) with 4-penten-1-ol (0.077464 M), measured at 256.5 nm. Here, $k_1 = 1/t_1 = 10.831$ min^{-1}, see Table S1.

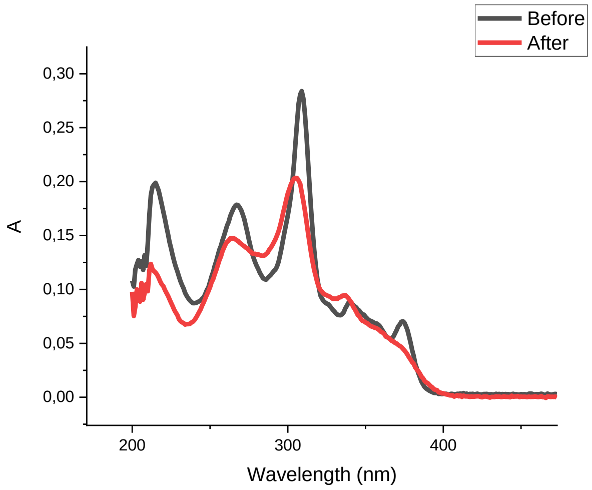

Figure 57. Absorbance (A, in Absorbance Units (AU)) vs wavelength plotted for **2** (0.000040483 M), before and after reaction with 4-penten-1-ol.

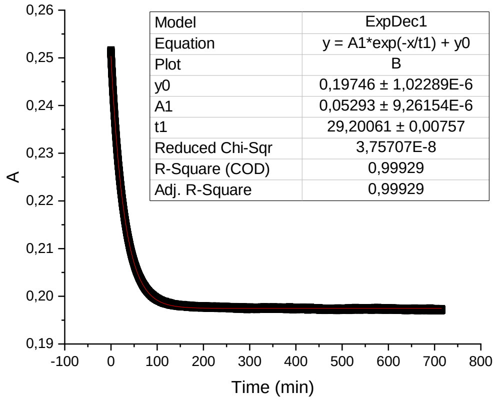

Figure S58. Absorbance (A, in Absorbance Units (AU)) vs time (min) plotted for the reaction of **2** (0.000040483 M) with 4-penten-1-ol (0.007746 M), measured at 307.5 nm. Here, $k_1 = 1/t_1 = 0.034$ min^{-1}, see Table S1.

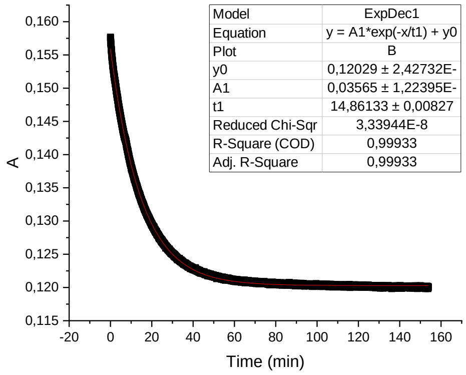

Figure S59. Absorbance (A, in Absorbance Units (AU)) vs time (min) plotted for the reaction of **2** (0.000040483 M) with 4-penten-1-ol (0.015493 M), measured at 307.5 nm. Here, $k_1 = 1/t_1 = 0.067$ min^{-1}, see Table S1.

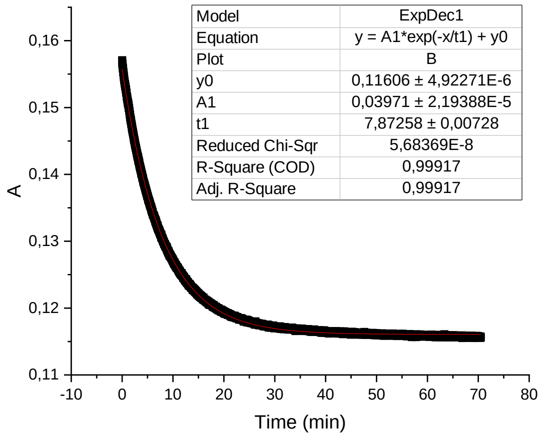

Figure S60. Absorbance (A, in Absorbance Units (AU)) vs time (min) plotted for the reaction of **2** (0.000040483 M) with 4-penten-1-ol (0.030986 M), measured at 307.5 nm. Here, $k_1 = 1/t_1 = 0.127$ min^{-1}, see Table S1.

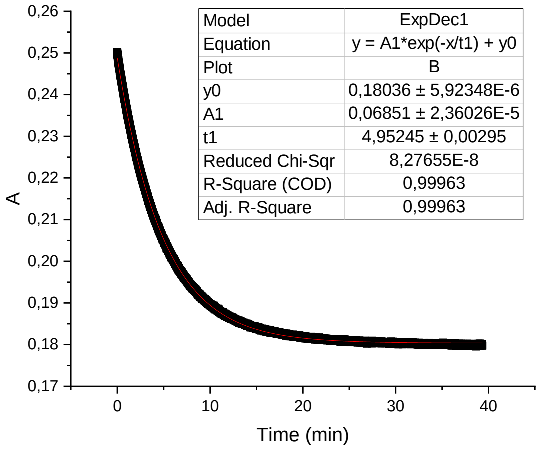

Figure S61. Absorbance (A, in Absorbance Units (AU)) vs time (min) plotted for the reaction of **2** (0.000040483 M) with 4-penten-1-ol (0.046479 M), measured at 307.5 nm. Here, $k_1 = 1/t_1 = 0.202$ min^{-1}, see Table S1.

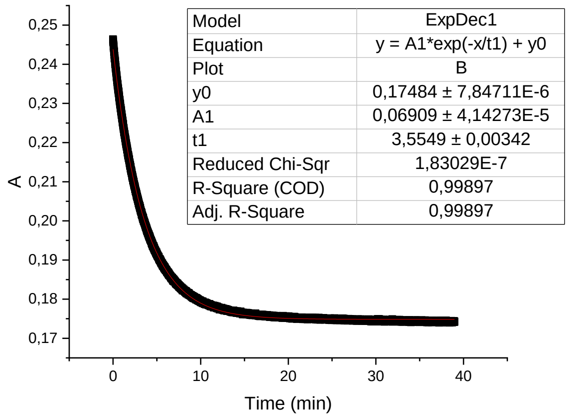

Figure S62. Absorbance (A, in Absorbance Units (AU)) vs time (min) plotted for the reaction of **2** (0.000040483 M) with 4-penten-1-ol (0.061971 M), measured at 307.5 nm. Here, $k_1 = 1/t_1 = 0.281$ min^{-1}, see Table S1.

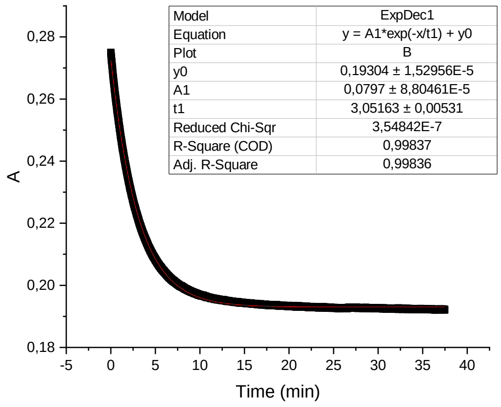

Figure S63. Absorbance (A, in Absorbance Units (AU)) vs time (min) plotted for the reaction of **2** (0.000040483 M) with 4-penten-1-ol (0.077464 M), measured at 307.5 nm. Here, $k_1 = 1/t_1 = 0.327$ min^{-1}, see Table S1.

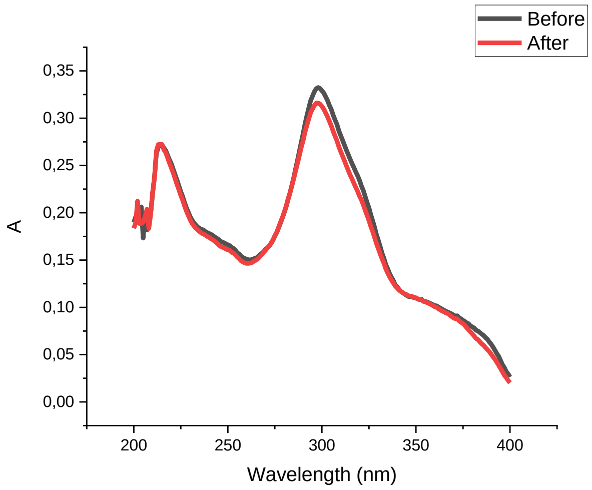

Figure S64. Absorbance (A, in Absorbance Units (AU)) vs wavelength plotted for **3** (0.000040298 M), before and after reaction with 4-penten-1-ol.

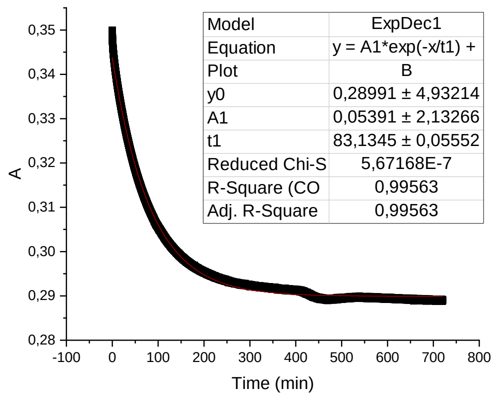

Figure S65. Absorbance (A, in Absorbance Units (AU)) vs time (min) plotted for the reaction of **3** (0.000040298 M) with 4-penten-1-ol (0.007746 M), measured at 298 nm. Here, $k_1 = 1/t_1 = 0.012$ min^{-1}, see Table S2.

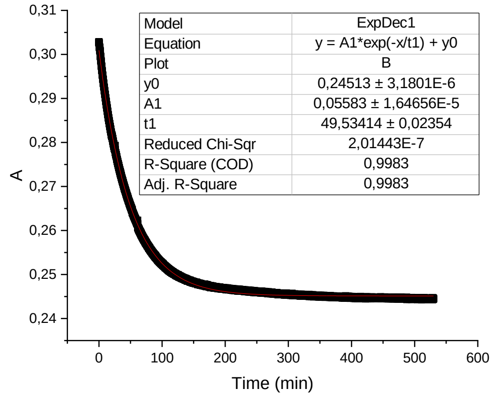

Figure S66. Absorbance (A, in Absorbance Units (AU)) vs time (min) plotted for the reaction of **3** (0.000040298 M) with 4-penten-1-ol (0.015493 M), measured at 298 nm. Here, $k_1 = 1/t_1 = 0.020$ min^{-1}, see Table S2.

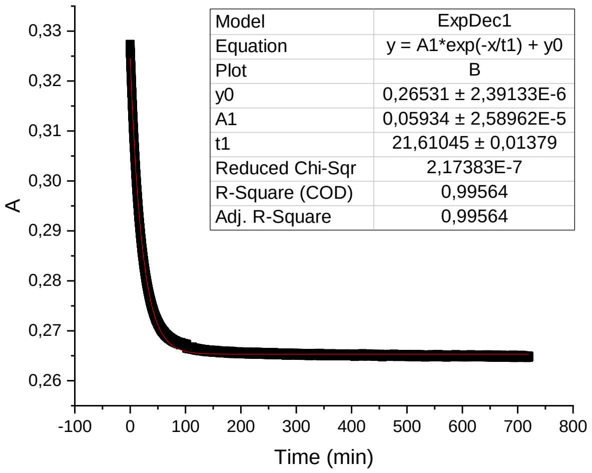

Figure S67. Absorbance (A, in Absorbance Units (AU)) vs time (min) plotted for the reaction of **3** (0.000040298 M) with 4-penten-1-ol (0.030986 M), measured at 298 nm. Here, $k_1 = 1/t_1 = 0.046$ min^{-1}, see Table S2.

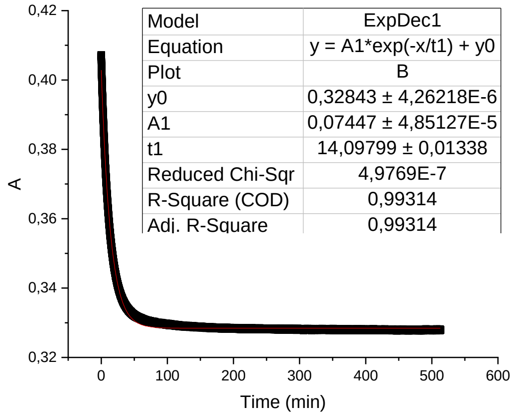

Figure S68. Absorbance (A, in Absorbance Units (AU)) vs time (min) plotted for the reaction of **3** (0.000040298 M) with 4-penten-1-ol (0.046479 M), measured at 298 nm. Here, $k_1 = 1/t_1 = 0.071$ min^{-1}, see Table S2.

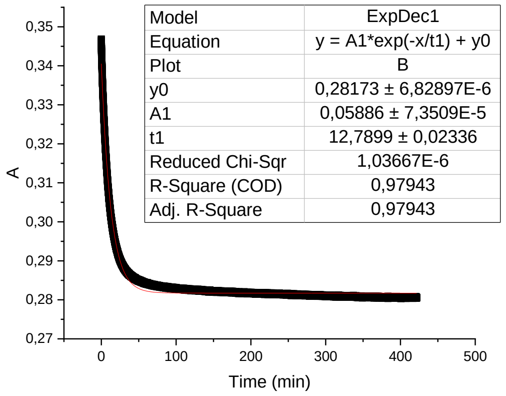

Figure S69. Absorbance (A, in Absorbance Units (AU)) vs time (min) plotted for the reaction of **3** (0.000040298 M) with 4-penten-1-ol (0.061971 M), measured at 298 nm. Here, $k_1 = 1/t_1 = 0.078$ min^{-1}, see Table S2.

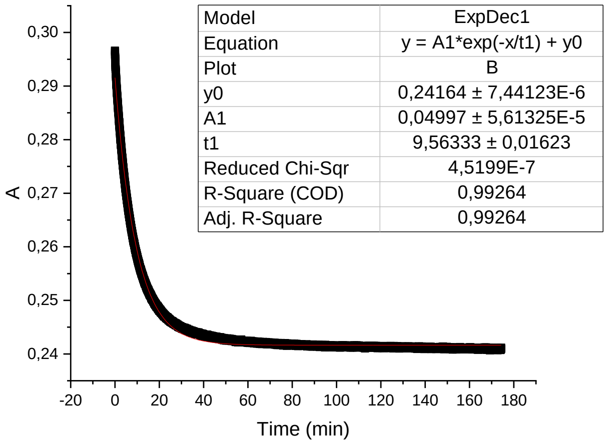

Figure S70. Absorbance (A, in Absorbance Units (AU)) vs time (min) plotted for the reaction of **3** (0.000040298 M) with 4-penten-1-ol (0.077464 M), measured at 298 nm. Here, $k_1 = 1/t_1 = 0.105$ min^{-1}, see Table S2.

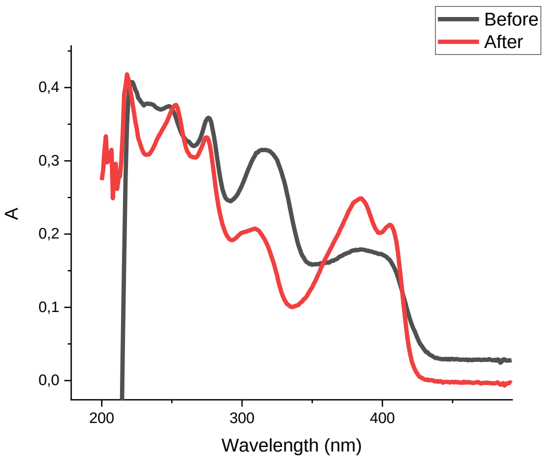

Figure S71. Absorbance (A, in Absorbance Units (AU)) vs wavelength plotted for **4** (0.000040862 M), before and after reaction with 4-penten-1-ol.

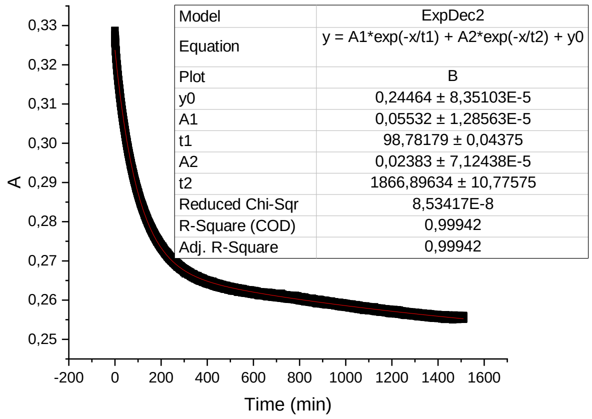

Figure S72. Absorbance (A, in Absorbance Units (AU)) vs time (min) plotted for the reaction of **4** (0.000040862 M) with 4-penten-1-ol (0.007746 M), measured at 316 nm. Here, $k_1 = 1/t_1 = 0.010$ min^{-1}, see Table S2.

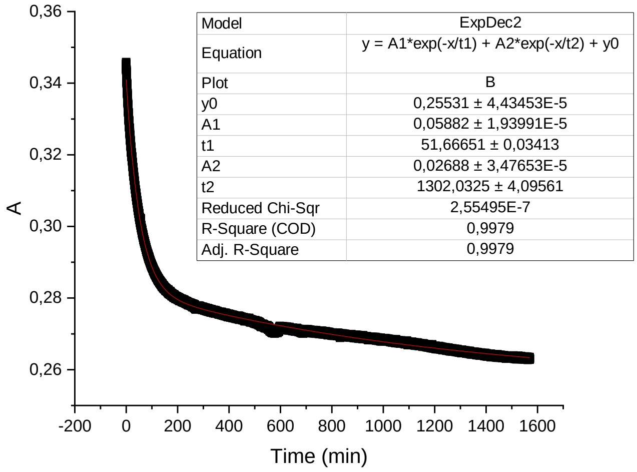

Figure S73. Absorbance (A, in Absorbance Units (AU)) vs time (min) plotted for the reaction of **4** (0.000040862 M) with 4-penten-1-ol (0.015493 M), measured at 316 nm. Here, $k_1 = 1/t_1 = 0.019$ min^{-1}, see Table S2.

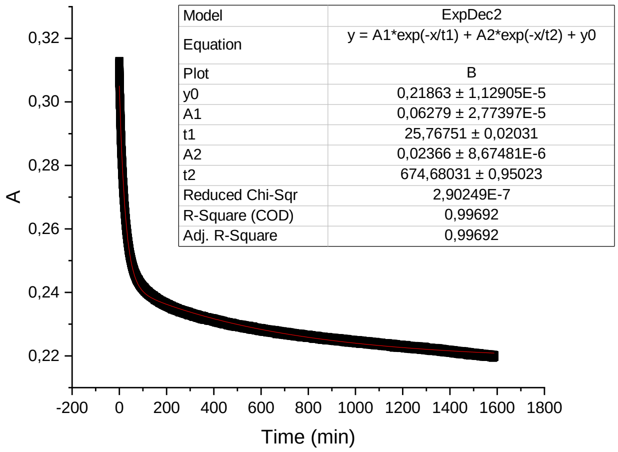

Figure S74. Absorbance (A, in Absorbance Units (AU)) vs time (min) plotted for the reaction of **4** (0.000040862 M) with 4-penten-1-ol (0.030986 M), measured at 316 nm. Here, $k_1 = 1/t_1 = 0.039$ min^{-1}, see Table S2.

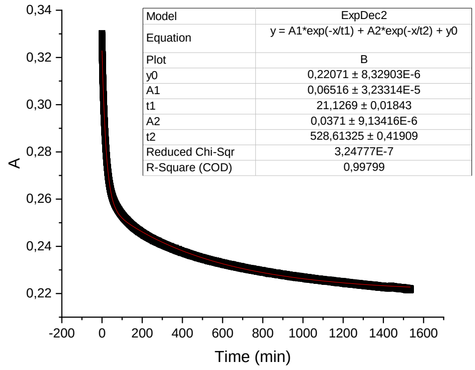

Figure S75. Absorbance (A, in Absorbance Units (AU)) vs time (min) plotted for the reaction of **4** (0.000040862 M) with 4-penten-1-ol (0.046479 M), measured at 316 nm. Here, $k_1 = 1/t_1 = 0.047$ min^{-1}, see Table S2.

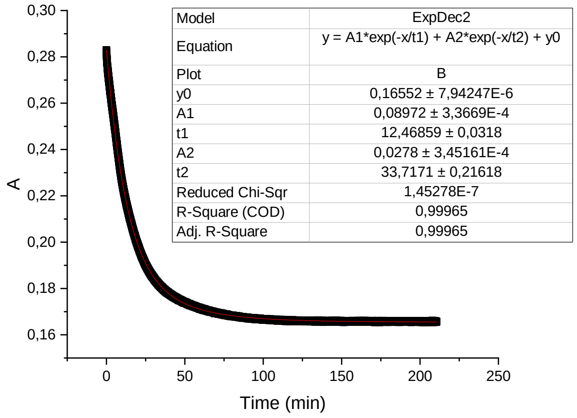

Figure S76. Absorbance (A, in Absorbance Units (AU)) vs time (min) plotted for the reaction of **4** (0.000040862 M) with 4-penten-1-ol (0.061971 M), measured at 316 nm. Here, $k_1 = 1/t_1 = 0.080$ min^{-1}, see Table S2.

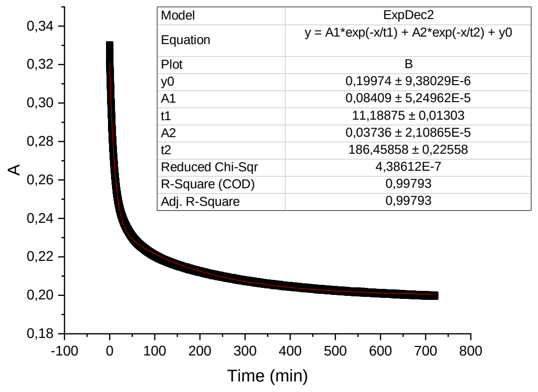

Figure S77. Absorbance (A, in Absorbance Units (AU)) vs time (min) plotted for the reaction of **4** (0.000040862 M) with 4-penten-1-ol (0.077464 M), measured at 316 nm. Here, $k_1 = 1/t_1 = 0.089$ min^{-1}, see Table S2.

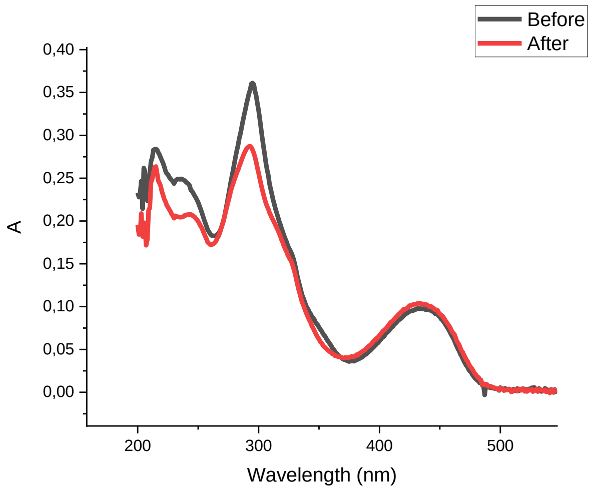

Figure S78. Absorbance (A, in Absorbance Units (AU)) vs wavelength plotted for **5** (0.000040483 M), before and after reaction with 4-penten-1-ol.

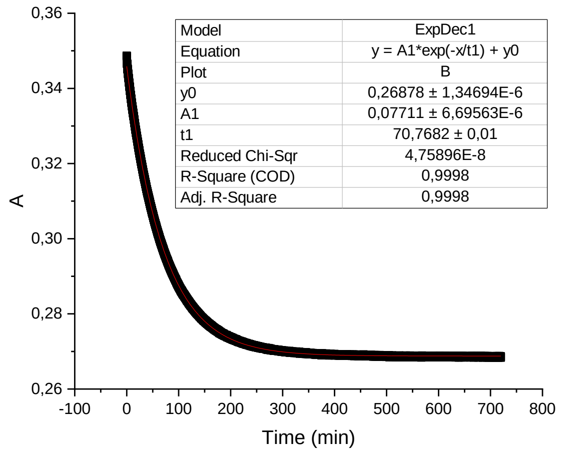

Figure S79. Absorbance (A, in Absorbance Units (AU)) vs time (min) plotted for the reaction of **5** (0.000040483 M) with 4-penten-1-ol (0.007746 M), measured at 295 nm. Here, $k_1 = 1/t_1 = 0.014$ min^{-1}, see Table S2.

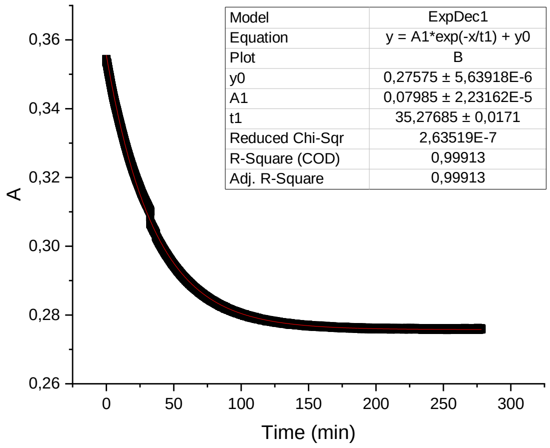

Figure S80. Absorbance (A, in Absorbance Units (AU)) vs time (min) plotted for the reaction of **5** (0.000040483 M) with 4-penten-1-ol (0.015493 M), measured at 295 nm. Here, $k_1 = 1/t_1 = 0.028\ min^{-1}$, see Table S2.

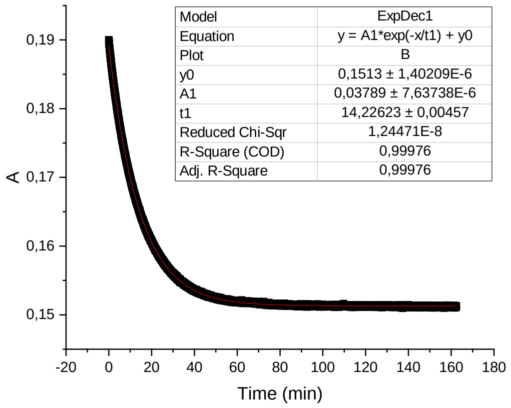

Figure S81. Absorbance (A, in Absorbance Units (AU)) vs time (min) plotted for the reaction of **5** (0.000040483 M) with 4-penten-1-ol (0.030986 M), measured at 295 nm. Here, $k_1 = 1/t_1 = 0.070\ min^{-1}$, see Table S2.

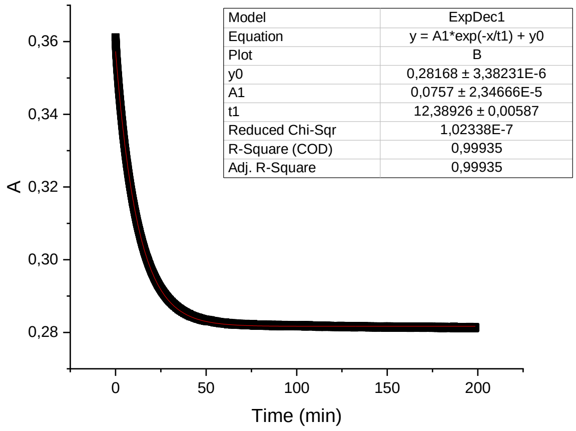

Figure S82. Absorbance (A, in Absorbance Units (AU)) vs time (min) plotted for the reaction of **5** (0.000040483 M) with 4-penten-1-ol (0.046479 M), measured at 295 nm. Here, $k_1 = 1/t_1 = 0.081\ min^{-1}$, see Table S2.

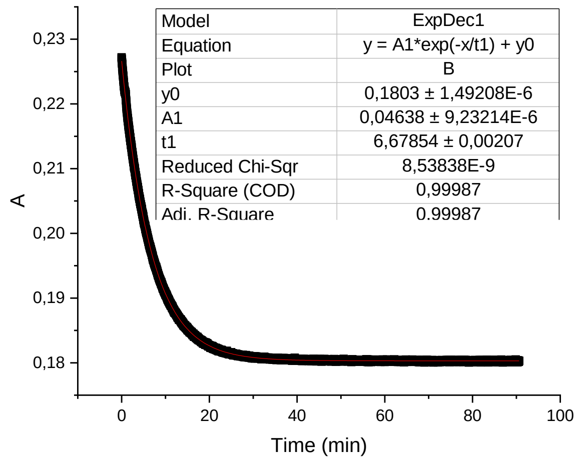

Figure S83. Absorbance (A, in Absorbance Units (AU)) vs time (min) plotted for the reaction of **5** (0.000040483 M) with 4-penten-1-ol (0.061971 M), measured at 295 nm. Here, $k_1 = 1/t_1 = 0.150$ min^{-1}, see Table S2.

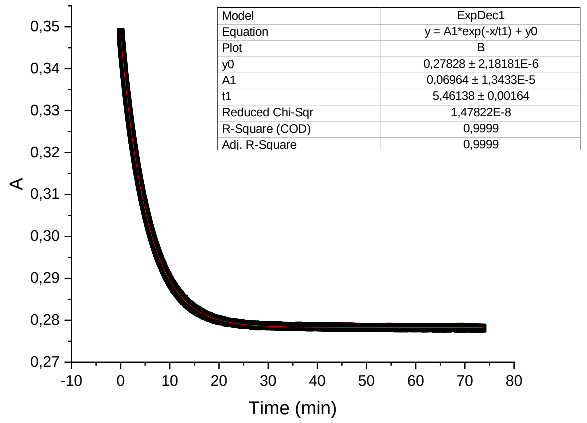

Figure S84. Absorbance (A, in Absorbance Units (AU)) vs time (min) plotted for the reaction of **5** (0.000040483 M) with 4-penten-1-ol (0.077464 M), measured at 295 nm. Here, $k_1 = 1/t_1 = 0.183$ min^{-1}, see Table S2.

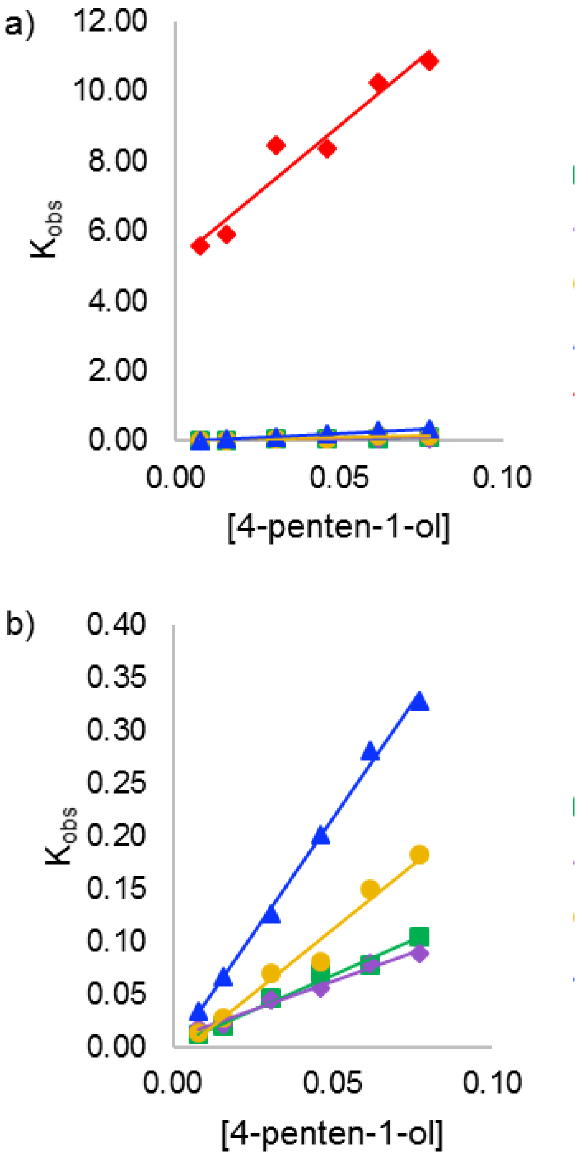

Figure S85. The second-order-rate constants (k_2, $M^{-1}s^{-1}$) of **1**-**5** in iodocyclization with 4-penten-1-ol in dry dicloroethane was obtained by fitting the observed pseudo-first order rate constant (k_{obs}) of each complex to the 4-penten-1-ol concentration. The slopes of the corresponding graphs provided the k_2 values 77.6 (R^2 = 0.94) for **1**, 4.3 (blue) for **2**, 1.4 (green) for **3**, 1.2 (violet) for **4**, and 2.3 (yellow) for **5**.

5. COMPUTATIONS

5.1. Computational approach

Density functional theory (DFT) was applied in our present work to describe the electronic structure of the investigated complexes, as well as the species (intermediates and transition states) involved in the explored reaction mechanism. Truhlar's M06-2X exchange-correlation functional[9] was used along with the Def2SVP basis set[10] for geometry optimizations and vibrational analysis. This choice for the approximated functional is justified by previous benchmark studies,[11-12] which reported good performance of the hybrid meta-GGA M06-2X functional for non-covalent and halogen bonding interactions, both important in our computational analysis. For each optimized structure, additional single-point energy calculations were performed with the larger Def2TZVPP basis set.[10] All these calculations (geometry optimizations and vibrational analysis as well) were carried out with the inclusion of solvent effects via the integral equation formalism of the polarizable continuum model (IEFPCM).[13] The atomic radii and non-electrostatic terms in the IEFPCM calculations were those introduced by Truhlar and coworkers in terms of the SMD solvation model.[14] For iodine, the atomic radius was set to 2.74 Å as suggested in the SMD18 refinement of the model.[15] We used dichloroethane as a solvent utilized in our kinetic experiments.

The thermal and entropic contributions to the Gibbs free energies were computed for 298.15 K and c = 1 mol/dm^3 conditions and employing Grimme's quasi-RRHO approximation.[16] This approach is expected to be more appropriate than the standard ideal gas RRHO (rigid rotor – harmonic oscillator) model, because the optimized structures of the present iodonium complexes have several low harmonic frequency modes (torsional motions and skeletal vibrations). The relative stabilities reported in our paper correspond to solution phase Gibbs free energies computed as $G = E_{0,sol}' + (G_{0,sol} - E_{0,sol})$, where $E_{0,sol}'$ and $E_{0,sol}$ are solution phase electronic energies obtained at the M06-2X/Def2TZVPP and M06-2X/Def2SVP levels, respectively, and $G_{0,sol}$ is solution phase Gibbs free energy computed at M06-2X/Def2SVP level. All DFT calculations were carried out with the *Gaussian16* software.[17] The molecular structures were visualized with the *CYLview*[18] program. The reduced density gradient (RDG) data in the noncovalent interaction (NCI) analysis were computed by using the *NCIPLOT* program.[19-20] These results were visualized with the *VMD* software.[21]

Our test calculations using the dispersion-corrected, range-separated hybrid ωB97X-D exchange-correlation functional[22-24] (and the same basis sets) gave very similar results for the relative stabilities of the species involved in the computational mechanistic study, therefore we think that the conclusions of our computational analysis should be invariant with respect to the choice of hybrid functionals.

5.2. Structural analysis of complexes 1-5

The structures optimized at the M06-2X/Def2SVP(SMD18) level of theory for the most stable forms of complexes **1**-**5** along with some structural data are depicted in Figure 2 of the main text. The optimized geometries of **1**-**5** did not have any imaginary frequencies. Additional structural information, namely the dihedral angles corresponding to the torsion of the aromatic units are shown in Figure S86. These data reveal that the distortion of the conjugated aromatic system is already notable for complex **3**, but becomes even more significant for **4** and **5**, where the central bicyclic aromatic groups interact with the adjacent pyridines.

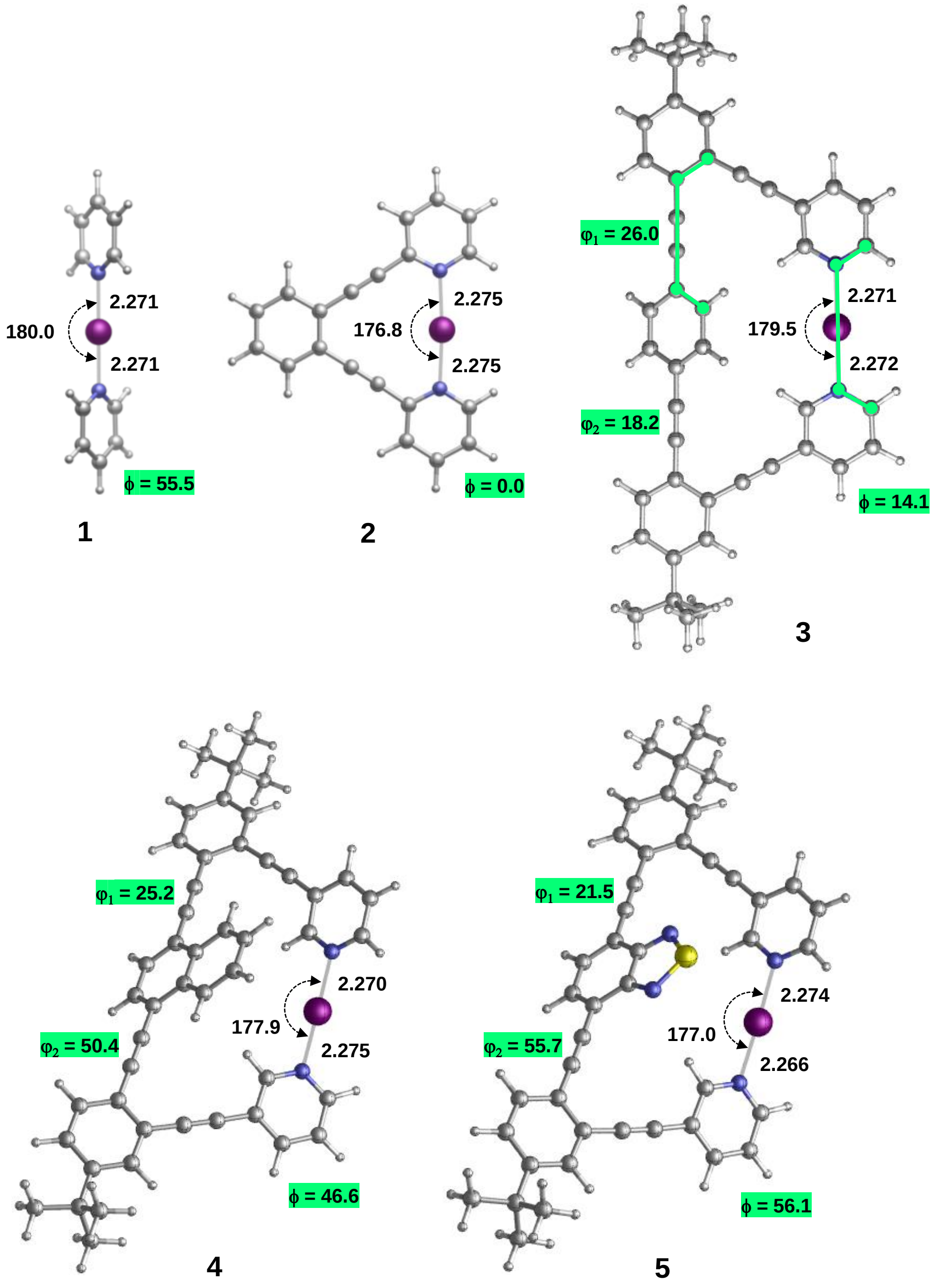

Figure S86. Optimized structures of the most stable forms of complexes **1**-**5**. Computed bond lengths are in Å, angles are in degrees. Bonds and atoms that define the ϕ and φ torsional angles are highlighted in green in complex **3**. Dihedral angle ϕ characterizes the torsion of the pyridine rings (coplanar arrangemeng corresponds to □ = 0), dihedral angle φ_i indicates torsion of the aromatic rings in the linker units of complexes **3**-**5**.

The *anti* conformers and their relative stabilities of complexes **4** and **5** are shown in Figure S87. Computations indicate that noncovalent aryl-aryl contacts between the central naphthalene and the pyridines in complex **4** provides slightly higher stabilization to the *syn* structure than those bewteeen the benzo[*c*][1,2,5]thiadiazole ring and pyridines in complex **5**. This is supported by the NCI analysis presented in the main text (Figure 3).

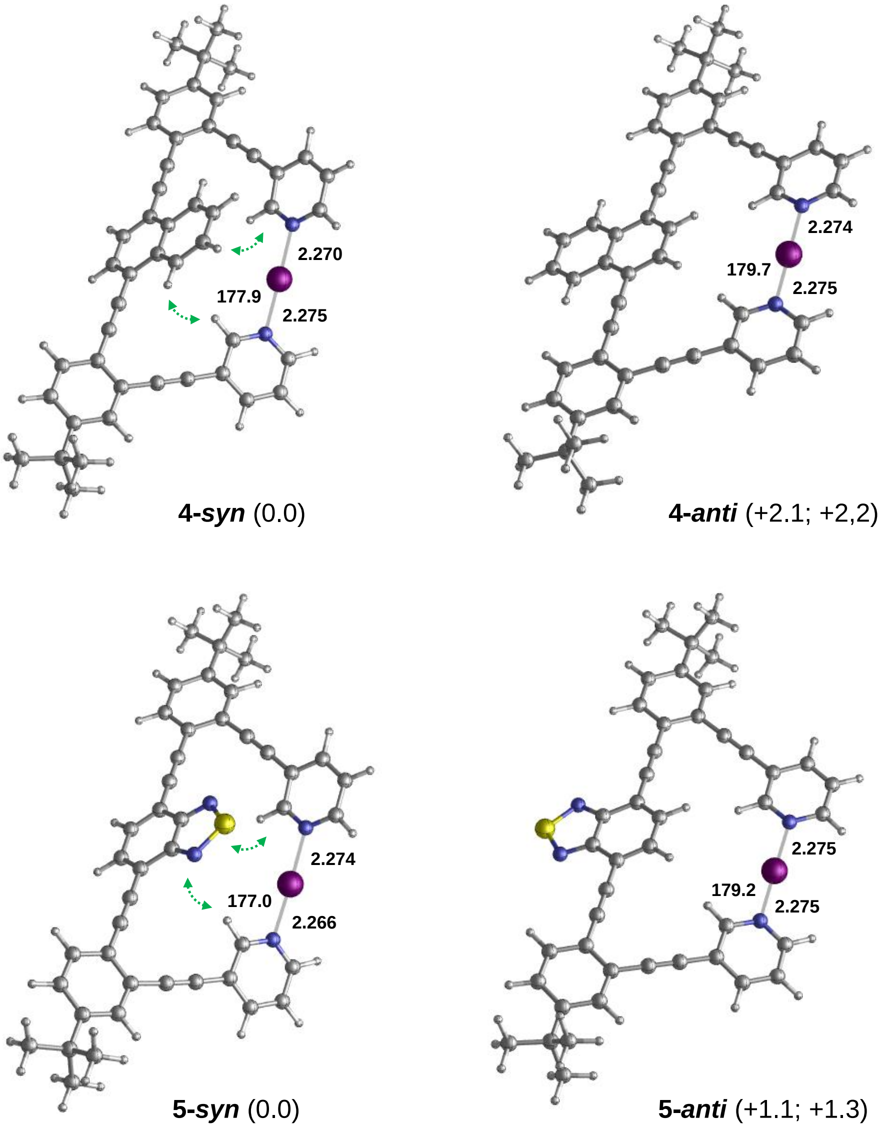

Figure S87. Optimized structures of the *syn* and *anti* forms of complexes **4** and **5**. Computed bond lengths are given in Å, whereas angles are given in degrees. Relative stabilities in terms of solution phase Gibbs energies and M06-2X/Def2TZVPP(SMD18) electronic energies, respectively, are shown in parenthesis (in kcal/mol).

5.3. Molecular electrostatic potentials of 3-5

To characterize the charge distribution in chelating complexes **3**-**5**, we computed the molecular electrostatic potential (ESP) maps and projected them to isodensity surfaces (Figure S88). These maps indicate that the positive charge of iodine(I) is distributed over the [bis(pyridine)iodine(I)] moiety, and the central aromatic rings are polarized such that the atoms in the vicinity of the [N-I-N] unit become more electropositive. We note that the S atom in **5** is electropositive as well.

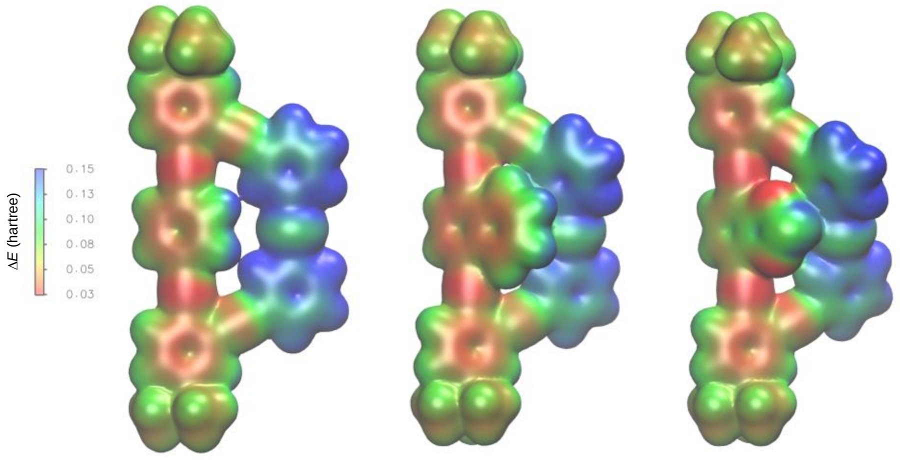

3 **4-*syn*** **5-*syn***

Figure S88. Electrostatic potential maps of the most stable forms of **3**-**5**. The computed potentials are projected to isodensity surfaces at 0.004 au. Blue colour represents electropositive regions, red illustrates more electron rich areas.

5.4. Potential energy scans for aryl rotations in 3-5

To gain insight into the steric and electronic effects imposed by the central aromatic ring in compounds **3**-**5**, we performed potential energy scan calculations by varying the $\ni_1$ dihedral angle (see Figure S86) in subsequent steps and optimizing all other parameters at each given φ_1 value. The obtained potential energy curves are shown in Figures S89-91. These results reveal nearly free rotation of the benzene ring in **3** (the rotational barrier is about 1.4 kcal/mol), but for complexes **4** and **5** the rotation is sterically hindered by the $[N\text{-}I\text{-}N]^+$ bond.

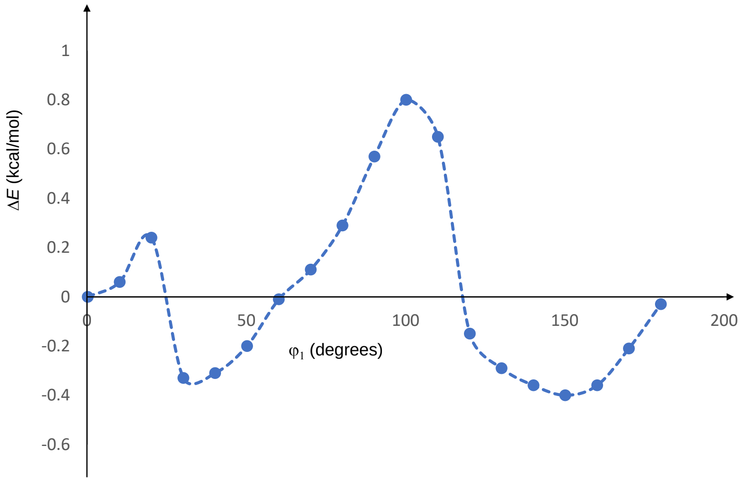

Figure S89. Potential energy curve derived for complex **3** by subsequent constrained geometry optimizations along dihedral angle φ_1.

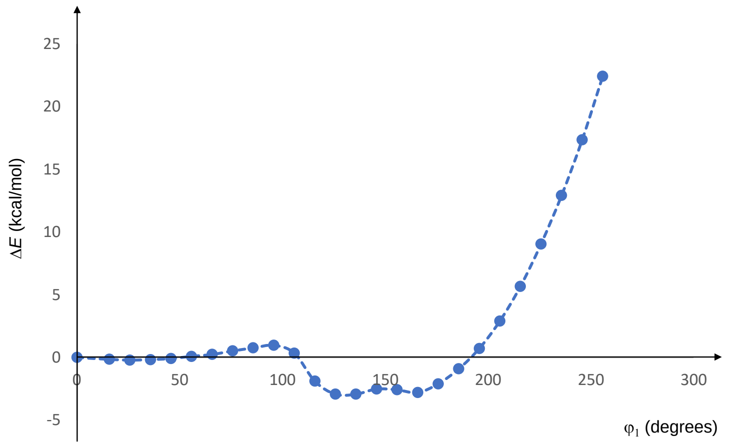

Figure S90. Potential energy curve derived for complex **4** by subsequent constrained geometry optimizations along dihedral angle φ_1.

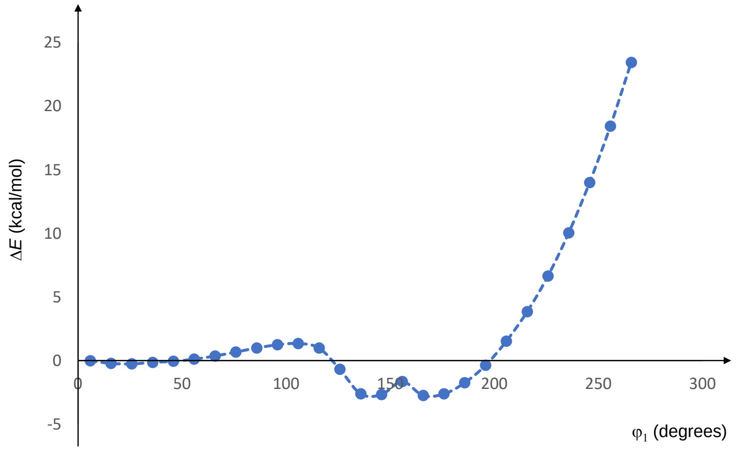

Figure S91. Potential energy curve derived for complex **5** by subsequent constrained geometry optimizations along dihedral angle φ_1.

5.5. Relative stabilities of complexes 1-5

The relative stabilities of iodine(I) complexes **1**-**5** were quantified by the free energy of the hypothetical isodesmic reactions shown in Figure S92. For this purpose, the geometries of the free ligands (i.e. without the I^+ ion) were optimized. The most stable forms of the ligand molecules (denoted as **L_1-L_5**) are depicted in Figure S93. All chelating ligands involve intramolecular C-H···N hydrogen bonding interactions (highlighted by dashed lines). Ligands **L_4** and **L_5** are characterized by aryl-aryl contacts, similarly to their complexed forms **4** and **5**.

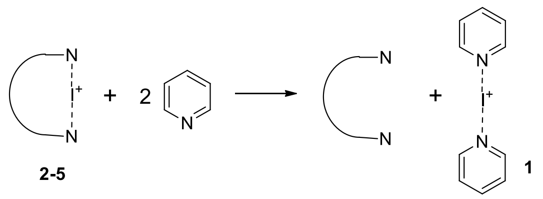

Figure S92. Isodesmic reactions used to estimate the relative stabilities of iodine(I) complexes.

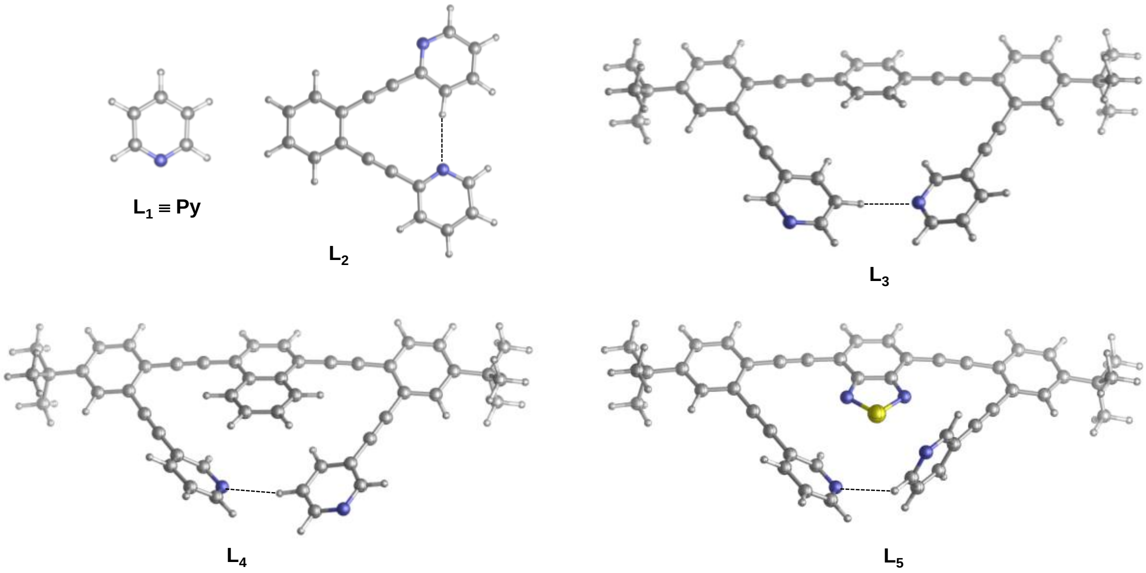

Figure S93. Most stable forms of ligands $\mathbf{L_1}$-$\mathbf{L_5}$.

5.6. Iodocyclization of pentenol with complex 1

The reaction intermediates and transition states identified computationally on the most favored reaction pathway for the iodocyclization of 4-penten-1-ol (**pent**) with complex **1** are reported in the main text (Figures 6 and 7). Herein, we present the species corresponding to the hydrogen bond assisted pathway (Figure S93).

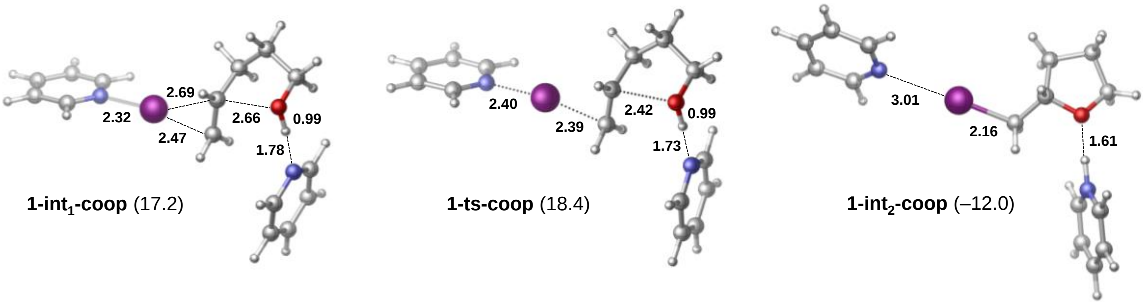

Figure S94. Transition state of iodocylization and the related intermediates identified on the H-bond assisted iodocyclization pathway in the reaction of **pent** with **1**. Bond distances are given in Å, relative stabilities in kcal/mol.

Along this cooperative reaction route, the pyridine molecule dissociated from **1** forms an H-bond with the OH group of **pent** prior to ring closure (intermediate $\mathbf{1\text{-}int_1\text{-}coop}$), and the assists the iodocyclization process. However, due to the entropic cost of molecular association, the predicted transition state (**1-ts-coop**) represents a slightly higher barrier as compared to the stepwise pathway (18.7 vs. 17.0 kcal/mol). In this mechanism, the deprotonation of the cyclic product occurs in a concerted-asynchronous manner with the iodonium transfer process, resulting in the $\mathbf{1\text{-}int_2\text{-}coop}$ intermediate.

5.7. Iodocyclization of pentenol with complex 2

Iodocyclization pathways associated with all four dissociated forms of complex **2** (Figure 8 of the main text) were systematically explored, and the transition states are reported in the paper (Figure 9). For the sake of completeness, herein we present the associated int_1 and int_2 intermediates as well (Figures S95-S98 for routes *a*, *b*, *c* and *d*, respectively).

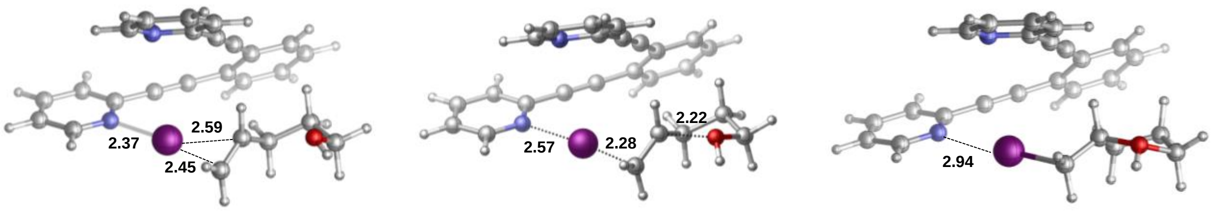

2-int$_{1a}$ (18.6) **2-ts$_a$** (22.6) **2-int$_{2a}$** (13.7)

Figure S95. Transition state of iodocylization and the related intermediates identified on iodocyclization pathway *a* in the reaction of **pent** with **2**. Bond distances are given in Å, relative stabilities in kcal/mol.

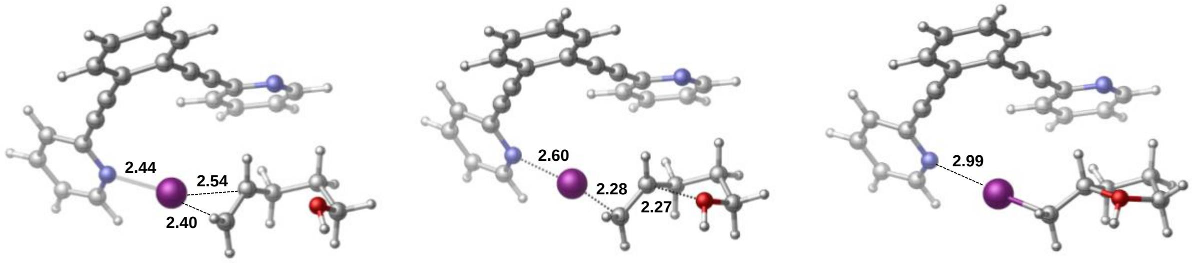

2-int$_{1b}$ (17.5) **2-ts$_b$** (20.9) **2-int$_{2b}$** (11.3)

Figure S96. Transition state of iodocylization and the related intermediates identified on iodocyclization pathway *b* in the reaction of **pent** with **2**. Bond distances are given in Å, relative stabilities in kcal/mol.

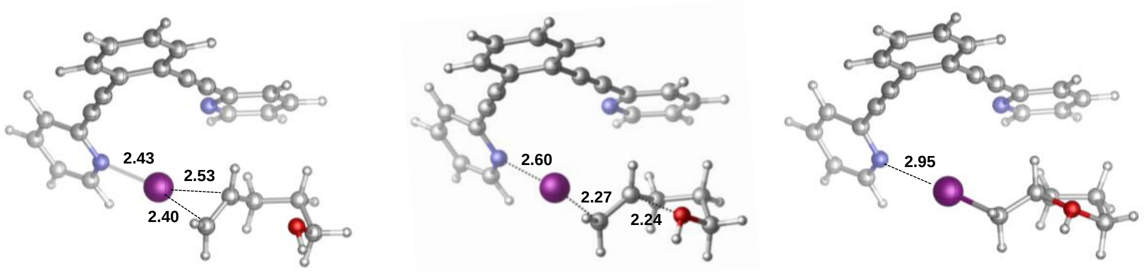

2-int$_{1c}$ (17.9) **2-ts$_c$** (21.9) **2-int$_{2c}$** (13.3)

Figure S97. Transition state of iodocylization and the related intermediates identified on iodocyclization pathway *c* in the reaction of **pent** with **2**. Bond distances are given in Å, relative stabilities in kcal/mol.

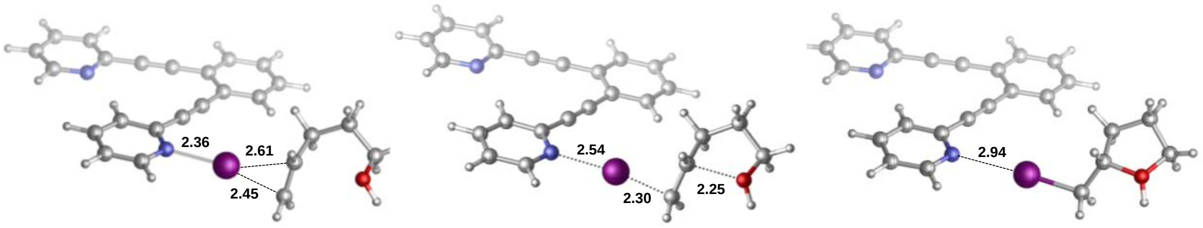

2-int$_{1d}$ (19.0) **2-ts$_d$** (22.5) **2-int$_{2d}$** (13.6)

Figure S98. Transition state of iodocylization and the related intermediates identified on iodocyclization pathway *d* in the reaction of **pent** with **2**. Bond distances are given in Å, relative stabilities in kcal/mol.

Additional transition states were found that differ from those presented above in the orientation of the double bond of **pent**, but they are predicted to be slightly less stable with respect to their counterparts (see Figure S99). We note that the proton transfer (from the cyclic product to the adjacent pyridine moiety occurs spontaneously on route *b′* leading to thermodynamically stable intermediate (**2-int$_{2b}$′**).

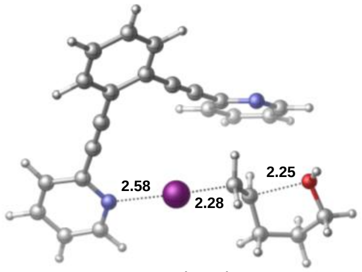

2-ts$_{b}$´ (21.2)

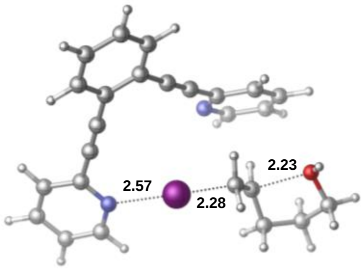

2-ts$_{c'}$ (22.3)

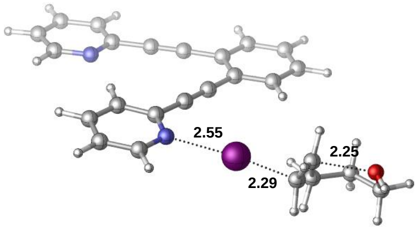

2-ts$_{d}$´ (23.0)

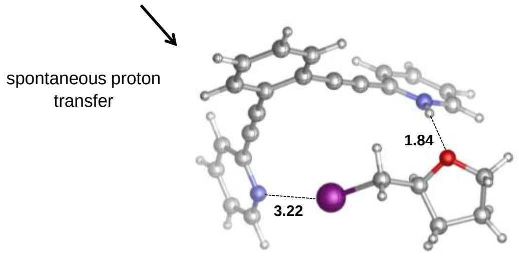

2-int$_{2b}$´ (-8.1)

Figure S99. Additional iodocylization transition states identified in the reaction of **pent** with **2**. Bond distances are given in Å, relative stabilities in kcal/mol.

5.8. Iodocyclization transition states for complexes 3-5

As noted in the paper, the increased size and the flexibility of complexes **3-5** did not allow us to carry out a systematic conformational search for the iodocyclization transition states in these reactions. However, we found a reasonable transition state for complex **3**, and analogous transition states were explored for complexes **4** and **5** as well. In these latter cases, the transition states were located for both *syn* and *anti*-orientation of the central aryl groups (naphthalene and benzo[*c*][1,2,5]thiadiazole rings). The results are summarized in Figures S100 and S101.

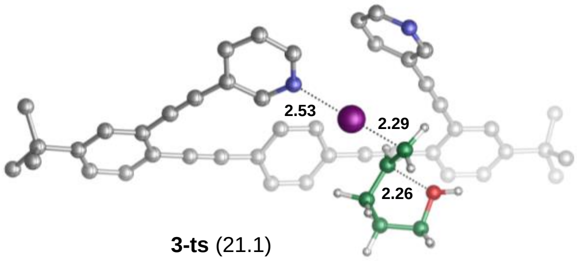

3-ts (21.1)

Figure S100. Transition state of iodocylization identified in the reaction of **pent** with **3** Bond distances are given in Å, relative stabilities in kcal/mol (with respect to reactant state **3** + **pent**). H atoms of the ligand are omitted for clarity.

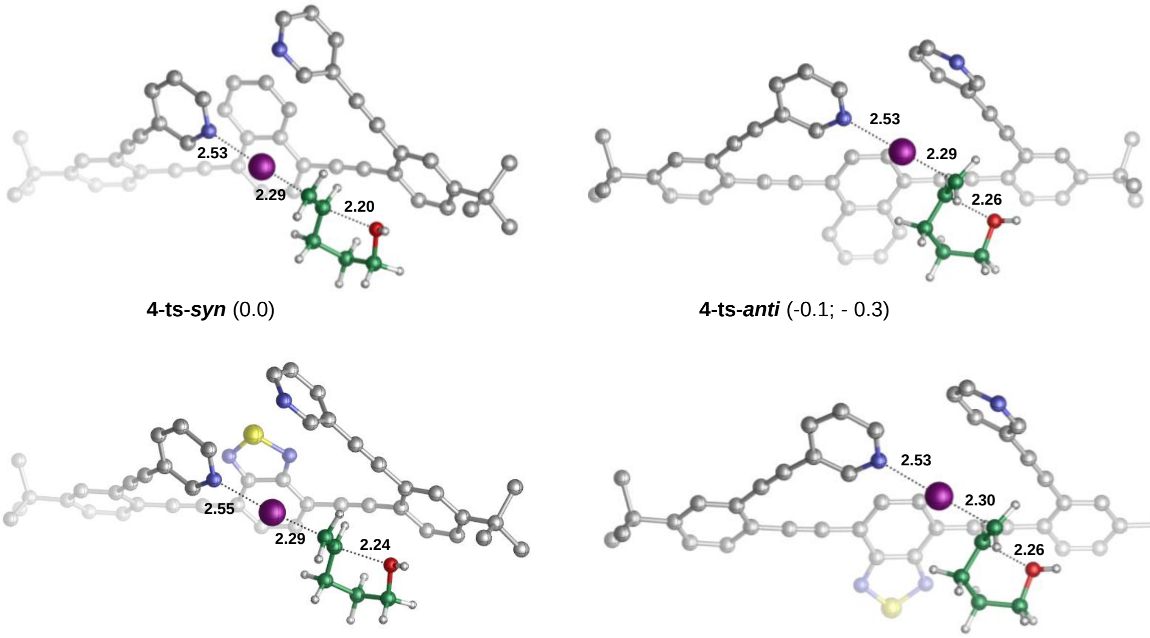

4-ts-*syn* (0.0)

4-ts-*anti* (-0.1; - 0.3)

5-ts-*syn* (0.0)

5-ts-*anti* (0.1; 0.7)

Figure S101. Transition states of iodocylization identified in the reaction of **pent** with the *syn* and *anti*-forms of complexes **4** and **5**. Bond distances are given in Å. Relative stabilities in terms of solution phase Gibbs energies and M06-2X/Def2TZVPP (SMD18) electronic energies, respectively, are shown in parenthesis (in kcal/mol).

Computations indicate that the relative stabilities of the transition state of the iodocyclization reactions of **4** and **5** are barely influenced by the orientation of the naphthalene and benzo[*c*][1,2,5]thiadiazole rings. Considering that the *syn*-orientation of complex **4** is more stabilized (2.1 kcal/mol) as compared to that of **5** (1.1 kcal/mol, Figure S87) by intramolecular aryl-aryl interactions, the barrier for the iodocylization reaction with **4** is slightly higher, which is in line with the observed reaction rates.

5.9. Total energy data

Table S3. Total energy data (in a.u.) computed for M06-2X/Def2SVP(SMD18) optimized structures.[a]

structure	$E_{0,sol}$	$G_{0,sol}$	$E_{0,sol}'$	G
**1**	-793.4717	-793.3269	-794.0324	-793.8846
**2**	-1175.2284	-1175.0100	-1176.1997	-1175.9782
**3**	-2103.1002	-2102.4957	-2105.0976	-2104.4900
**4-*syn***	-2256.5810	-2255.9304	-2258.7412	-2258.0876
**4-*anti***	-2256.5765	-2255.9259	-2258.7378	-2258.0842
**5-*syn***	-2609.3201	-2608.7260	-2611.5860	-2610.9889
**5-*anti***	-2609.3155	-2608.7218	-2611.5839	-2610.9871
**L_1**	-247.9982	-247.9359	-248.2774	-248.2121
**L_2**	-877.7662	-877.5485	-878.7323	-878.5115
**L_3**	-1805.6351	-1805.0333	-1807.6269	-1807.0221
**L_4-*syn***	-1959.1152	-1958.4679	-1961.2702	-1960.6198
**L_4-*anti***	-1959.1115	-1958.4642	-1961.2672	-1960.6169
**L_5-*syn***	-2311.8556	-2311.2640	-2314.1159	-2313.5212
**L_5-*anti***	-2311.8506	-2311.2601	-2314.1135	-2313.5200
**pent**	-271.4122	-271.3012	-271.7341	-271.6200
**prod**	-568.4649	-568.3625	-568.7888	-568.6834
**1-H**	-496.4669	-496.3120	-497.0184	-496.8605
**1-dis**	-545.4246	-545.3645	-545.7126	-545.6494
**1-int$_1$**	-816.8623	-816.6694	-817.4703	-817.2744
**1-int$_1$'**	-816.8633	-816.6695	-817.4700	-817.2732
**1-ts**	-816.8598	-816.6650	-817.4633	-817.2655
**1-int$_2$**	-816.8806	-816.6831	-817.4801	-817.2796
**1-int$_3$**	-1064.9347	-1064.6538	-1065.8077	-1065.5237
**1-int$_1$-coop**	-1064.8769	-1064.5991	-1065.7580	-1065.4772
**1-ts-coop**	-1064.8766	-1064.5985	-1065.7564	-1065.4753
**1-int$_2$-coop**	-1064.9347	-1064.6539	-1065.8077	-1065.5238
**2-H**	-878.2212	-877.9890	-879.1850	-878.9498
**2-dis$_a$**	-1175.1891	-1174.9718	-1176.1682	-1175.9478
**2-dis$_b$**	-1175.1919	-1174.9743	-1176.1682	-1175.9475
**2-dis$_c$**	-1175.1919	-1174.9742	-1176.1682	-1175.9475
**2-dis$_d$**	-1175.1919	-1174.9743	-1176.1682	-1175.9475
**2-int$_{1a}$**	-1446.6305	-1446.2777	-1447.9245	-1447.5687
**2-ts$_a$**	-1446.6278	-1446.2740	-1447.9190	-1447.5622
**2-int$_{2a}$**	-1446.6487	-1446.2918	-1447.9364	-1447.5764
**2-int$_{1a}$**	-1446.6327	-1446.2796	-1447.9266	-1447.5704
**2-ts$_b$**	-1446.6306	-1446.2765	-1447.9220	-1447.5649
**2-int$_{2b}$**	-1446.6533	-1446.2963	-1447.9403	-1447.5803
**2-int$_{1c}$**	-1446.6318	-1446.2786	-1447.9259	-1447.5698
**2-ts$_c$**	-1446.6286	-1446.2748	-1447.9201	-1447.5633
**2-int$_{2c}$**	-1446.6496	-1446.2925	-1447.9372	-1447.5770
**2-int$_{1d}$**	-1446.6291	-1446.2778	-1447.9224	-1447.5681
**2-ts$_d$**	-1446.6266	-1446.2748	-1447.9174	-1447.5625
**2-int$_{2d}$**	-1446.6490	-1446.2932	-1447.9355	-1447.5767
**3-dis**	-2103.0623	-2102.4590	-2105.0647	-2104.4584
**3-ts**	-2374.5058	-2373.7639	-2376.8214	-2376.0765
**4-ts-*syn***	-2527.9860	-2527.1989	-2530.4618	-2529.6717
**4-ts-*anti***	-2527.9829	-2527.1954	-2530.4623	-2529.6718
**5-ts-*syn***	-2880.7282	-2879.9976	-2883.3088	-2882.5752
**5-ts-*anti***	-2880.7214	-2879.9917	-2883.3077	-2882.5750

[a] $G = E_{0,sol}' + (G_{0,sol} - E_{0,sol}) + \Delta G_{conc}$, where $E_{0,sol}'$ and $E_{0,sol}$ are solution phase electronic energies obtained at the M06-2X/Def2TZVPP(SMD18) and M06-2X/Def2SVP(SMD18) levels, respectively, and $G_{0,sol}$ is solution phase Gibbs free energy computed at M06-2X/Def2SVP(SMD18) level using the *quasi*-RRHO approximation. ΔG_{conc} = 0.003019 a.u. corresponding to c = 1 mol/dm^3 concentration.

5.10. Cartesian coordinates

Cartesian coordinates of the optimized geometries of molecular models are given below in standard XYZ format (units are in Å). The first line shows the number of atoms, the second line is the notation used for structures discussed in the main text and the SI (see above in Table S3).

```
23
1
C   -2.929861    -0.559876    -1.030419
C   -4.317623    -0.586994    -1.062627
C   -5.025123    -0.031968     0.002032
C   -4.323501     0.533230     1.065223
C   -2.935544     0.525677     1.030442
N   -2.271230    -0.012638    -0.000513
H   -6.116146    -0.039709     0.003113
H   -2.327750    -0.980788    -1.838575
H   -4.826510    -1.039266    -1.913361
H   -4.837108     0.978354     1.916895
H   -2.337897     0.955188     1.837393
I   -0.000009     0.000047    -0.001272
C    2.935937    -0.525709     1.029168
C    4.323909    -0.533454     1.063310
C    5.025121     0.031795    -0.000126
C    4.317208     0.587074    -1.064375
C    2.929457     0.560117    -1.031544
N    2.271221     0.012823    -0.001415
H    6.116146     0.039375     0.000454
H    2.338591    -0.955263     1.836320
H    4.837848    -0.978780     1.914676
H    4.825763     1.039410    -1.915274
H    2.327052     0.981201    -1.839390

35
2
C    0.691098    -5.541087     0.000103
C    1.391658    -4.339059     0.000033
C    0.704961    -3.115755    -0.000022
C   -0.713380    -3.114121    -0.000009
C   -1.402864    -4.335856     0.000060
C   -0.705026    -5.539480     0.000116
H    1.238602    -6.484352     0.000152
H   -2.493348    -4.327956     0.000078
H   -1.254679    -6.481496     0.000175
C   -1.456240    -1.889057    -0.000011
C   -2.107040    -0.862979    -0.000001
C   -2.973372     2.669177     0.000075
C   -4.362430     2.670964    -0.000367
C   -5.030878     1.448854    -0.000746
C   -4.296530     0.267903    -0.000641
C   -2.898912     0.332756    -0.000175
N   -2.272295     1.531624     0.000145
H   -6.121039     1.413458    -0.001118
H   -2.399953     3.598215     0.000349
H   -4.899586     3.618590    -0.000423
H   -4.780906    -0.707637    -0.000904
C    1.450723    -1.892465    -0.000036
C    2.104125    -0.868048    -0.000037
C    2.979506     2.661817     0.000045
C    4.368566     2.660031    -0.000433
```

C	5.033897	1.436220	-0.000844
C	4.296544	0.257144	-0.000731
C	2.899090	0.325611	-0.000227
N	2.275533	1.526059	0.000120
H	6.123957	1.398057	-0.001247
H	2.408496	3.592328	0.000343
H	4.908135	3.606288	-0.000491
H	4.778435	-0.719625	-0.001016
H	2.482155	-4.333600	0.000032
I	0.001639	1.591923	0.000280

83

3

C	-0.011733	0.002181	-0.001882
C	-0.008556	-0.031549	1.397747
C	-1.233562	0.126721	2.065740
C	-2.395642	0.307810	1.325333
C	-2.318088	0.325110	-0.061185
N	-1.143169	0.173130	-0.687119
H	-1.262795	0.106167	3.155841
H	0.914703	-0.111731	-0.569360
H	-3.361891	0.434764	1.812193
H	-3.201751	0.461168	-0.687833
C	1.223222	-0.225263	2.098915
C	2.274830	-0.392157	2.683155
C	3.518000	-0.580852	3.368956
C	4.733859	-0.640708	2.641177
C	3.535080	-0.697823	4.763373
C	5.925744	-0.814375	3.353842
C	4.727976	-0.873777	5.476112
H	2.581649	-0.646388	5.292170
C	5.920726	-0.929017	4.742075
H	6.867755	-0.861137	2.806213
H	6.874289	-1.064453	5.251153
C	4.747447	-0.522366	1.213682
C	4.769241	-0.419086	0.002020
C	4.811514	-0.280919	-1.424702
C	3.697817	-0.625702	-2.211774
C	5.972087	0.207251	-2.051680
C	3.741113	-0.477605	-3.591193
H	2.800447	-1.017878	-1.730665
C	6.014942	0.356425	-3.430420
H	6.838947	0.472248	-1.445248
C	4.898495	0.019965	-4.216748
H	2.879191	-0.753166	-4.200903
H	6.915384	0.739195	-3.912162
C	4.942814	0.184006	-5.640139
C	4.988754	0.329279	-6.846758
C	5.053109	0.492814	-8.267766
C	6.287258	0.609382	-8.917403
C	3.872649	0.535972	-9.052953
C	6.357865	0.757073	-10.300610
H	7.202266	0.580245	-8.324641
C	3.966058	0.684125	-10.441295
C	5.201693	0.794693	-11.092024
H	7.342354	0.842791	-10.759066
H	3.038514	0.714640	-11.015727
C	2.587158	0.434524	-8.428933
C	1.499631	0.353446	-7.894138
C	0.224075	0.248370	-7.254487
C	0.142636	0.280392	-5.857017
C	-0.968159	0.104754	-7.982434

H	1.038000	0.399130	-5.242705
C	-2.174486	-0.004401	-7.300916
H	-0.937607	0.077935	-9.072373
C	-2.172435	0.027843	-5.912480
H	-3.117078	-0.117628	-7.835214
H	-3.092383	-0.059240	-5.331123
N	-1.028613	0.167402	-5.228878
C	5.240189	0.948121	-12.614989
C	4.579659	-0.280528	-13.261161
H	3.528462	-0.386270	-12.955164
H	4.603748	-0.184702	-14.357475
H	5.112561	-1.202954	-12.984713
C	4.466744	2.214841	-13.014663
H	4.917135	3.109667	-12.558874
H	4.489201	2.337441	-14.108342
H	3.413281	2.163388	-12.702845
C	6.673100	1.064203	-13.139458
H	7.188522	1.942535	-12.722833
H	7.266618	0.168634	-12.902205
H	6.652435	1.173790	-14.233593
C	4.683771	-0.991834	7.001936
C	6.078576	-1.196842	7.596976
H	6.745005	-0.350745	7.372086
H	5.999002	-1.280629	8.690683
H	6.548904	-2.118112	7.221749
C	3.803087	-2.190104	7.390856
H	2.772623	-2.074088	7.024376
H	4.210532	-3.126147	6.979662
H	3.762930	-2.284598	8.486793
C	4.084166	0.296347	7.588982
H	4.693173	1.171854	7.317244
H	3.058037	0.467643	7.231739
H	4.052003	0.227190	8.687114
I	-1.079794	0.178165	-2.957121

89

4-syn

N	0.112307	0.189860	0.000630
C	0.045131	0.105208	1.330265
C	-0.975241	0.743768	2.046496
C	-1.944887	1.457230	1.324838
C	-1.856334	1.516440	-0.061146
C	-0.802821	0.874513	-0.698938
C	-0.977662	0.673670	3.475584
C	-0.927489	0.622800	4.688103
C	-0.822434	0.547717	6.114860
C	0.452191	0.380237	6.715025
C	0.523802	0.289844	8.109515
C	-0.626646	0.369477	8.890839
C	-1.892468	0.542349	8.314290
C	-1.964813	0.628406	6.917679
C	1.634799	0.318269	5.910358
C	2.635455	0.277851	5.219526
C	3.824359	0.219538	4.423135
C	3.866576	0.839820	3.126552
C	5.060683	0.753956	2.353085
C	6.194803	0.051162	2.887954
C	6.120280	-0.522220	4.147240
C	4.942427	-0.439085	4.909856
C	7.406418	-0.062210	2.129347
C	8.442129	-0.173534	1.501379
C	9.661175	-0.306822	0.758880

C	9.647846	-0.766562	-0.582780
C	10.849337	-0.884187	-1.289962
C	12.082287	-0.556581	-0.710980
C	12.078182	-0.110698	0.617625
C	10.893120	0.011069	1.340235
C	8.406325	-1.116697	-1.206148
C	7.344287	-1.416307	-1.713596
C	6.073595	-1.749373	-2.280699
C	4.904530	-1.315738	-1.644277
N	3.699467	-1.593201	-2.143560
C	3.562600	-2.305881	-3.269845
C	4.675454	-2.776236	-3.955619
C	5.944579	-2.495142	-3.462963
I	1.883487	-0.736859	-1.074531
C	13.362448	-0.690955	-1.540106
C	13.516664	-2.148324	-2.003375
C	-3.177772	0.634880	9.140994
C	-2.898622	0.532558	10.642022
C	-3.862763	1.981771	8.859411
C	-4.118497	-0.511721	8.737088
C	14.604794	-0.299923	-0.736793
C	13.262389	0.228727	-2.767880
H	6.836678	-2.846395	-3.982986
H	4.948360	-0.728837	-0.723350
H	4.539413	-3.353480	-4.869489
H	2.545958	-2.493529	-3.620720
H	10.803388	-1.241967	-2.320005
H	10.917152	0.362765	2.372258
H	13.012109	0.152688	1.112882
C	5.100955	1.382060	1.078124
H	6.986167	-1.049682	4.548361
C	2.754474	1.550932	2.598651
H	4.904896	-0.902592	5.896059
H	1.497601	0.160253	8.583249
H	-0.520135	0.295911	9.972530
H	-2.929959	0.758685	6.424811
H	0.815582	-0.475429	1.842902
H	-2.752544	1.962521	1.855695
H	-2.591109	2.062485	-0.651703
H	-0.678885	0.905224	-1.783195
H	-4.128806	2.088444	7.797557
H	-4.789162	2.060343	9.448726
H	-3.206888	2.821297	9.135674
H	-3.647883	-1.489123	8.922783
H	-5.047046	-0.459266	9.325948
H	-4.390206	-0.457858	7.672622
H	-2.433106	-0.429185	10.904616
H	-2.240394	1.342990	10.989246
H	-3.846621	0.609034	11.194310
H	14.736726	-0.942818	0.146431
H	15.497260	-0.410310	-1.369894
H	14.558645	0.746555	-0.400227
H	12.406343	-0.036171	-3.405694
H	13.150449	1.279986	-2.461936
H	14.176285	0.142484	-3.375402
H	13.579398	-2.829707	-1.141362
H	12.673472	-2.467463	-2.633291
H	14.438611	-2.256190	-2.595003
C	4.007785	2.059202	0.591521
C	2.821408	2.144494	1.359855
H	6.020059	1.323527	0.491727
H	4.054165	2.536733	-0.388668

H	1.958176	2.687420	0.968485
H	1.843288	1.621102	3.197299

89

4-anti

N	-4.672317	0.215491	0.662538
C	-5.314947	-0.950594	0.579366
C	-6.690227	-1.043792	0.822484
C	-7.383113	0.132937	1.150462
C	-6.689124	1.334722	1.224408
C	-5.322696	1.344473	0.975332
C	-7.345910	-2.313024	0.736268
C	-7.906254	-3.388924	0.670470
C	-8.591070	-4.645317	0.592592
C	-7.875072	-5.850339	0.375140
C	-8.596977	-7.047701	0.299705
C	-9.982498	-7.060544	0.438850
C	-10.704818	-5.879851	0.660055
C	-9.983472	-4.681469	0.731346
C	-6.450415	-5.855474	0.241047
C	-5.238833	-5.882850	0.132385
C	-3.812568	-5.894453	0.012173
C	-3.119572	-7.088508	-0.389842
C	-1.698563	-7.065064	-0.501388
C	-0.990856	-5.847249	-0.210962
C	-1.694752	-4.718183	0.176934
C	-3.094422	-4.741880	0.288894
C	0.435822	-5.791245	-0.315431
C	1.648402	-5.734318	-0.399135
C	3.076097	-5.705823	-0.497329
C	3.793448	-4.482380	-0.520526
C	5.189948	-4.498762	-0.616531
C	5.914041	-5.695421	-0.690428
C	5.189395	-6.895035	-0.668796
C	3.800187	-6.901755	-0.574775
C	3.103998	-3.227901	-0.454115
C	2.535735	-2.155253	-0.405943
C	1.859632	-0.894814	-0.357905
C	0.480112	-0.855222	-0.124986
N	-0.185619	0.299797	-0.083385
C	0.444644	1.468981	-0.260591
C	1.813675	1.513051	-0.491707
C	2.531772	0.324195	-0.542322
I	-2.430022	0.263570	0.288261
C	7.440879	-5.647678	-0.792398
C	7.836260	-4.882993	-2.065826
C	-12.226541	-5.852344	0.824966
C	-12.835491	-7.252201	0.720173
C	-12.572133	-5.268207	2.204142
C	-12.837200	-4.967105	-0.273304
C	8.050888	-7.049796	-0.854288
C	8.007257	-4.920410	0.437827
H	3.606862	0.328222	-0.725916
H	-0.088124	-1.773467	0.030636
H	2.304687	2.475372	-0.632131
H	-0.162873	2.375180	-0.215854
H	5.712284	-3.540541	-0.633042
H	3.259416	-7.848924	-0.558295
H	5.706935	-7.851924	-0.725120
C	-1.020360	-8.248654	-0.898484
H	-1.151834	-3.800416	0.401142
C	-3.812847	-8.294295	-0.679162

H	-3.626299	-3.843577	0.601754
H	-8.057483	-7.980849	0.132041
H	-10.498261	-8.017907	0.372843
H	-10.505850	-3.737953	0.899835
H	-4.730338	-1.833440	0.314291
H	-8.455536	0.093959	1.345315
H	-7.196902	2.265303	1.475741
H	-4.734185	2.262763	1.025328
H	-12.197193	-4.240219	2.315362
H	-13.664513	-5.245524	2.337934
H	-12.139576	-5.881157	3.009398
H	-12.596745	-5.359936	-1.272856
H	-13.932605	-4.942462	-0.167769
H	-12.469499	-3.932306	-0.212813
H	-12.636518	-7.709589	-0.260547
H	-12.447866	-7.923444	1.501149
H	-13.926127	-7.185365	0.845093
H	7.698855	-7.608061	-1.734677
H	9.145390	-6.967632	-0.923902
H	7.813890	-7.636260	0.046051
H	7.642243	-3.884894	0.503506
H	7.728486	-5.443030	1.365426
H	9.105912	-4.887023	0.378790
H	7.440425	-5.383390	-2.962590
H	7.457991	-3.850308	-2.053753
H	8.932886	-4.840288	-2.151331
C	-1.718207	-9.401053	-1.170732
C	-3.129183	-9.424139	-1.060003
H	0.067090	-8.228876	-0.984640
H	-1.183848	-10.302694	-1.473916
H	-3.674605	-10.343433	-1.278719
H	-4.900452	-8.309548	-0.593622

84

5-*syn*

N	0.054748	0.115386	0.005135
C	0.013529	0.086199	1.338364
C	-0.991226	0.755850	2.048350
C	-1.966830	1.451076	1.317650
C	-1.901426	1.459080	-0.071137
C	-0.867781	0.781396	-0.703641
C	-0.983176	0.714987	3.478437
C	-0.941401	0.662644	4.690891
C	-0.843607	0.575062	6.117445
C	0.424116	0.372837	6.722166
C	0.489732	0.265350	8.115608
C	-0.661916	0.360272	8.893304
C	-1.921247	0.565795	8.313225
C	-1.986527	0.670524	6.917213
C	1.604819	0.294559	5.918064
C	2.598036	0.243757	5.218167
C	3.753115	0.201257	4.382567
C	3.713583	0.793622	3.069905
C	4.873212	0.752780	2.209581
C	6.093485	0.134286	2.659641
C	6.088340	-0.409920	3.926868
C	4.941324	-0.381010	4.770581
C	7.256008	0.116947	1.828039
C	8.264401	0.098303	1.148704
C	9.465509	0.069721	0.365805
C	9.468397	-0.468261	-0.946468
C	10.655542	-0.492535	-1.685192

C	11.858313	0.004945	-1.165980
C	11.838059	0.531886	0.132346
C	10.666507	0.563156	0.885870
C	8.254696	-0.992266	-1.497848
C	7.205167	-1.431344	-1.921798
C	5.931384	-1.918240	-2.354308
C	4.775479	-1.410986	-1.749709
N	3.564117	-1.843283	-2.102846
C	3.407232	-2.776791	-3.051186
C	4.506996	-3.322518	-3.700981
C	5.782795	-2.892841	-3.353160
I	1.776922	-0.910790	-1.067508
C	13.125945	-0.049035	-2.022770
C	13.435963	-1.514057	-2.370241
C	-3.208041	0.671826	9.135727
C	-2.936831	0.549265	10.636688
C	-3.869331	2.032767	8.865493
C	-4.165710	-0.455137	8.716281
C	14.332553	0.546545	-1.294118
C	12.892624	0.745646	-3.317667
H	6.664850	-3.305408	-3.844329
H	4.837740	-0.640361	-0.975882
H	4.356437	-4.078688	-4.470676
H	2.385018	-3.080811	-3.285048
H	10.624477	-0.914064	-2.691482
H	10.677296	0.977597	1.894593
H	12.748048	0.930350	0.579448
N	4.669662	1.346661	1.040090
H	6.997294	-0.881505	4.301475
N	2.684050	1.424885	2.520237
H	5.010244	-0.834624	5.759654
H	1.458894	0.109784	8.591011
H	-0.561945	0.271974	9.974477
H	-2.947012	0.826045	6.422612
H	0.789564	-0.476824	1.861806
H	-2.762857	1.980170	1.842872
H	-2.641762	1.990043	-0.668509
H	-0.766356	0.765953	-1.790564
H	-4.130166	2.155129	7.804078
H	-4.796238	2.120581	9.452670
H	-3.200507	2.858260	9.152681
H	-3.712096	-1.441904	8.894659
H	-5.095892	-0.392959	9.301579
H	-4.431616	-0.386760	7.651215
H	-2.489358	-0.423140	10.891224
H	-2.266084	1.344532	10.994778
H	-3.885687	0.636589	11.185853
H	14.561311	-0.003293	-0.368922
H	15.216149	0.489356	-1.946421
H	14.171221	1.604494	-1.038346
H	12.062353	0.328634	-3.906282
H	12.662146	1.798679	-3.095734
H	13.798103	0.715807	-3.942859
H	13.602888	-2.106591	-1.457938
H	12.616807	-1.979889	-2.937441
H	14.346497	-1.567133	-2.986521
S	3.150229	1.919720	1.045801

84

5-anti

N	-4.672895	0.166495	0.673485
C	-5.342443	-0.984013	0.585606

C	-6.716643	-1.048067	0.845746
C	-7.378901	0.139623	1.195877
C	-6.657044	1.324477	1.274479
C	-5.294175	1.306000	1.007471
C	-7.400148	-2.301762	0.751929
C	-7.973918	-3.369528	0.674101
C	-8.646338	-4.630469	0.571499
C	-7.907092	-5.819350	0.341888
C	-8.604148	-7.027998	0.230970
C	-9.991023	-7.066775	0.352309
C	-10.736596	-5.903521	0.588358
C	-10.038771	-4.692932	0.691091
C	-6.481247	-5.788895	0.233762
C	-5.267944	-5.765942	0.153500
C	-3.845753	-5.743341	0.056668
C	-3.139831	-6.863870	-0.509518
C	-1.700417	-6.845326	-0.615090
C	-0.948056	-5.704780	-0.158769
C	-1.665211	-4.657495	0.381968
C	-3.084356	-4.676914	0.488666
C	0.473064	-5.690811	-0.277408
C	1.684220	-5.687863	-0.387926
C	3.109481	-5.691112	-0.513209
C	3.842222	-4.477048	-0.551592
C	5.235917	-4.511928	-0.668644
C	5.941222	-5.719822	-0.749931
C	5.200639	-6.909420	-0.716573
C	3.812718	-6.897917	-0.601747
C	3.159982	-3.219242	-0.483402
C	2.575739	-2.155422	-0.436530
C	1.874690	-0.909133	-0.386729
C	0.493236	-0.898024	-0.159572
N	-0.192402	0.245156	-0.110839
C	0.417494	1.426734	-0.278502
C	1.785590	1.497879	-0.507083
C	2.525155	0.322705	-0.562043
I	-2.433375	0.191935	0.274957
C	7.467002	-5.693408	-0.872351
C	7.856224	-4.931334	-2.149263
C	-12.260117	-5.907011	0.737927
C	-12.842704	-7.314937	0.597129
C	-12.628695	-5.358903	2.126086
C	-12.876454	-5.009294	-0.346998
C	8.056531	-7.103669	-0.945138
C	8.058983	-4.976702	0.352131
H	3.600629	0.346914	-0.741703
H	-0.059651	-1.828586	-0.015611
H	2.258868	2.470016	-0.640613
H	-0.205760	2.321969	-0.227863
H	5.771400	-3.561331	-0.697291
H	3.257613	-7.836332	-0.577265
H	5.704265	-7.873212	-0.780250
N	-1.207359	-7.950434	-1.157301
H	-1.127645	-3.781405	0.746295
N	-3.678479	-7.982157	-0.975947
H	-3.586317	-3.817533	0.934371
H	-8.044578	-7.946608	0.050610
H	-10.489170	-8.031241	0.260538
H	-10.580331	-3.762277	0.869253
H	-4.781667	-1.877413	0.302633
H	-8.449477	0.122168	1.403483
H	-7.140252	2.263449	1.542368

H	-4.684724	2.210458	1.059559
H	-12.272319	-4.327428	2.263472
H	-13.722541	-5.357558	2.249313
H	-12.193582	-5.982102	2.922046
H	-12.618578	-5.375296	-1.352370
H	-13.973091	-5.008119	-0.252166
H	-12.529866	-3.969099	-0.260149
H	-12.626048	-7.747413	-0.391107
H	-12.450843	-7.995951	1.367427
H	-13.935495	-7.269985	0.712464
H	7.684530	-7.655063	-1.821619
H	9.151000	-7.036555	-1.029641
H	7.823686	-7.688612	-0.042756
H	7.708015	-3.936827	0.425565
H	7.786093	-5.498443	1.281969
H	9.157060	-4.957148	0.278306
H	7.440669	-5.423367	-3.041709
H	7.494027	-3.893047	-2.129395
H	8.952082	-4.905033	-2.249600
S	-2.462429	-8.921193	-1.499044

11

L$_1$

C	-2.937819	-0.623347	-0.969581
C	-4.331721	-0.663878	-1.013843
C	-5.046169	-0.034438	0.003325
C	-4.337271	0.606124	1.017432
C	-2.943108	0.587499	0.967103
N	-2.251985	-0.012529	-0.002726
H	-6.137759	-0.042926	0.005660
H	-2.350926	-1.109404	-1.755219
H	-4.840278	-1.178720	-1.829666
H	-4.850266	1.113048	1.835431
H	-2.360521	1.082724	1.750219

34

L$_2$

C	0.641093	-5.137562	-0.087549
C	1.372508	-3.954820	-0.133483
C	0.720843	-2.714327	-0.074227
C	-0.693584	-2.675189	0.033420
C	-1.414642	-3.877552	0.078557
C	-0.751197	-5.099176	0.018590
H	1.160473	-6.095661	-0.134910
H	-2.501460	-3.840293	0.161500
H	-1.323657	-6.027040	0.054487
C	-1.369179	-1.413135	0.093934
C	-1.907127	-0.324847	0.142169
C	-2.290047	3.247180	0.227627
C	-3.668763	3.453995	0.308921
C	-4.504192	2.340473	0.334890
C	-3.934976	1.072206	0.279535
C	-2.538343	0.969945	0.199841
N	-1.732310	2.042036	0.174140
H	-5.587358	2.455951	0.397695
H	-1.606660	4.101326	0.205099
H	-4.069292	4.467079	0.350361
H	-4.546193	0.169885	0.296855
C	1.462681	-1.490262	-0.121947
C	2.049836	-0.427609	-0.160272
C	4.596361	2.105045	-0.357311
C	3.892214	3.309639	-0.291804

C	2.505228	3.260785	-0.175177
C	1.877014	2.019955	-0.129160
C	2.678426	0.869736	-0.203288
N	4.013480	0.911070	-0.314969
H	1.916298	4.178067	-0.120951
H	5.686625	2.112243	-0.448921
H	4.426760	4.259072	-0.331924
H	0.792140	1.925732	-0.038229
H	2.459655	-3.977456	-0.216494

82

L_3

C	1.966850	0.040383	1.077771
C	1.442021	-1.198853	0.668694
C	0.088846	-1.249020	0.299505
C	-0.666290	-0.084110	0.361737
C	-0.041882	1.090386	0.785017
N	1.244824	1.153008	1.132935
H	-0.351799	-2.191630	-0.029924
H	3.019498	0.108357	1.369103
H	-1.725710	-0.084361	0.095230
H	-0.611359	2.022905	0.844213
C	2.266493	-2.370536	0.635996
C	2.950417	-3.375025	0.604221
C	3.733232	-4.574611	0.548029
C	3.093335	-5.835437	0.425297
C	5.130158	-4.511794	0.601552
C	3.889001	-6.983877	0.348452
C	5.927252	-5.661885	0.531250
H	5.593679	-3.528309	0.698044
C	5.278208	-6.897110	0.399839
H	3.406085	-7.957009	0.251174
H	5.855501	-7.818799	0.337378
C	1.664272	-5.922755	0.391862
C	0.449142	-5.969367	0.378375
C	-0.984538	-5.984755	0.371557
C	-1.692622	-7.021067	-0.261941
C	-1.697286	-4.946437	0.998775
C	-3.080784	-7.013228	-0.278861
H	-1.143780	-7.829562	-0.746203
C	-3.084439	-4.937751	0.980626
H	-1.150272	-4.148866	1.503318
C	-3.792787	-5.968614	0.336605
H	-3.626664	-7.815999	-0.775850
H	-3.634387	-4.132586	1.469672
C	-5.226156	-5.942361	0.311142
C	-6.441300	-5.895934	0.299267
C	-7.871607	-5.823917	0.305464
C	-8.649889	-6.983871	0.221144
C	-8.531435	-4.572190	0.410010
C	-10.040784	-6.916863	0.248084
H	-8.151669	-7.950541	0.137852
C	-9.929970	-4.529112	0.439468
C	-10.709661	-5.690467	0.361680
H	-10.603484	-7.847203	0.180847
H	-10.408973	-3.551885	0.522641
C	-7.770499	-3.358576	0.471423
C	-7.116880	-2.334360	0.506947
C	-6.328657	-1.137827	0.537054
C	-4.993890	-1.159671	0.092474
C	-6.846163	0.080500	1.001738
H	-4.567239	-2.095315	-0.281463

C	-6.020529	1.198214	1.004034
H	-7.877206	0.138347	1.353789
C	-4.709970	1.064246	0.545524
H	-6.382516	2.163761	1.357615
H	-4.037248	1.927044	0.540037
N	-4.209439	-0.088934	0.098379
C	-12.236184	-5.576778	0.404689
C	-12.654980	-4.937589	1.738539
H	-12.230816	-3.929716	1.856656
H	-13.751440	-4.850249	1.784410
H	-12.322633	-5.551784	2.589197
C	-12.711567	-4.690944	-0.758024
H	-12.414488	-5.121448	-1.726396
H	-13.809138	-4.607939	-0.740556
H	-12.296328	-3.674647	-0.692123
C	-12.913075	-6.944185	0.284728
H	-12.660930	-7.440735	-0.664232
H	-12.630073	-7.612420	1.111796
H	-14.004774	-6.814091	0.316516
C	7.451057	-5.526727	0.600269
C	8.149370	-6.884141	0.490672
H	7.921027	-7.382988	-0.463089
H	9.238391	-6.738576	0.541793
H	7.861326	-7.557406	1.311911
C	7.838387	-4.883015	1.941407
H	7.397620	-3.881657	2.053613
H	7.501054	-5.503028	2.785874
H	8.932542	-4.779697	2.005443
C	7.932548	-4.632854	-0.553745
H	7.659192	-5.067569	-1.527218
H	7.499588	-3.623572	-0.494732
H	9.028179	-4.532120	-0.517298

88

L_4-*syn*

C	1.940345	0.028635	1.192673
C	1.427523	-1.190550	0.713766
C	0.087193	-1.220973	0.298831
C	-0.668191	-0.057893	0.387274
C	-0.056587	1.095549	0.881086
N	1.218377	1.139747	1.272456
H	-0.343584	-2.146687	-0.086465
H	2.983009	0.081068	1.520702
H	-1.718517	-0.044372	0.087578
H	-0.626595	2.026061	0.962521
C	2.252539	-2.361050	0.658040
C	2.941095	-3.361596	0.607185
C	3.735399	-4.552248	0.527183
C	3.110505	-5.815982	0.364040
C	5.130991	-4.476736	0.597032
C	3.919253	-6.953868	0.264541
C	5.941019	-5.616113	0.503569
H	5.583116	-3.491504	0.724937
C	5.306699	-6.854172	0.332175
H	3.447566	-7.929137	0.136852
H	5.894358	-7.767788	0.250948
C	1.683838	-5.920098	0.312940
C	0.469500	-5.988393	0.285264
C	-0.961709	-6.024093	0.266496
C	-1.661931	-7.121713	-0.342327
C	-1.675043	-4.982560	0.839661
C	-3.086970	-7.114905	-0.358240

C	-3.080295	-4.974765	0.822521
H	-1.136049	-4.161011	1.312622
C	-3.790277	-6.009934	0.234075
H	-3.621408	-4.146101	1.280617
C	-5.221341	-5.965009	0.222995
C	-6.436095	-5.899168	0.226651
C	-7.864790	-5.814122	0.250040
C	-8.652265	-6.967580	0.157005
C	-8.514290	-4.559420	0.379495
C	-10.042114	-6.891408	0.199132
H	-8.161574	-7.936457	0.055033
C	-9.912141	-4.507391	0.424468
C	-10.700840	-5.661984	0.337726
H	-10.612231	-7.816620	0.124043
H	-10.383470	-3.528222	0.526770
C	-7.745867	-3.351070	0.449938
C	-7.091095	-2.327837	0.492475
C	-6.307054	-1.128754	0.530963
C	-4.980707	-1.134515	0.061547
C	-6.822452	0.077706	1.028005
H	-4.556050	-2.060141	-0.338585
C	-6.003045	1.199910	1.036473
H	-7.847237	0.122965	1.399606
C	-4.700396	1.082031	0.551760
H	-6.363767	2.156559	1.414645
H	-4.032492	1.948590	0.550482
N	-4.202046	-0.059553	0.073570
C	-12.225930	-5.537987	0.397430
C	-12.627005	-4.912390	1.743095
H	-12.194456	-3.909102	1.869454
H	-13.722300	-4.817870	1.800779
H	-12.290800	-5.539436	2.582791
C	-12.705736	-4.634350	-0.749675
H	-12.422155	-5.055677	-1.726095
H	-13.802324	-4.542358	-0.719973
H	-12.281001	-3.622538	-0.675912
C	-12.913881	-6.898878	0.267321
H	-12.675068	-7.385212	-0.690324
H	-12.627213	-7.579444	1.082989
H	-14.004222	-6.761406	0.311986
C	7.462479	-5.466853	0.591546
C	8.176565	-6.813197	0.451570
H	7.962791	-7.287925	-0.517773
H	9.263384	-6.657484	0.517468
H	7.887998	-7.511908	1.251071
C	7.829456	-4.856303	1.953755
H	7.376830	-3.863063	2.088635
H	7.490215	-5.502722	2.777400
H	8.921742	-4.743420	2.031892
C	7.945269	-4.536307	-0.532559
H	7.687950	-4.947939	-1.520338
H	7.498812	-3.534506	-0.451376
H	9.039074	-4.423146	-0.481171
C	-3.773422	-8.201897	-0.963257
C	-3.078967	-9.246912	-1.524239
H	-4.864474	-8.193893	-0.975409
C	-0.972082	-8.214892	-0.932059
C	-1.663387	-9.253553	-1.508456
H	-3.619226	-10.075241	-1.985131
H	0.119012	-8.216726	-0.919637
H	-1.120708	-10.086939	-1.957227

88

L_4-*anti*

C	0.161423	0.219760	-0.036631
C	0.054460	0.086872	1.360096
C	0.936925	-0.792318	2.006212
C	1.877118	-1.476035	1.243367
C	1.894195	-1.266346	-0.136008
N	1.053912	-0.440402	-0.763756
H	0.881538	-0.925639	3.087866
H	-0.515553	0.898573	-0.564477
H	2.601087	-2.147133	1.709522
H	2.620906	-1.793012	-0.762073
C	-0.914948	0.845096	2.094769
C	-1.720706	1.496539	2.730900
C	-2.663552	2.249672	3.505432
C	-2.399220	2.528518	4.871680
C	-3.853319	2.704175	2.926078
C	-3.351480	3.246175	5.604109
C	-4.804965	3.427348	3.657083
H	-4.028815	2.475566	1.873328
C	-4.530431	3.686160	5.007026
H	-3.157599	3.463523	6.655222
H	-5.240803	4.242842	5.617151
C	-1.178100	2.086456	5.474306
C	-0.128090	1.699774	5.951742
C	1.109367	1.214975	6.484496
C	2.249546	1.059704	5.620821
C	1.205325	0.871320	7.823582
C	3.455791	0.520611	6.154321
C	2.397373	0.342568	8.350422
H	0.338328	0.996888	8.472805
C	3.507281	0.150000	7.543492
H	2.444940	0.064490	9.403714
C	4.699069	-0.434680	8.080373
C	5.715520	-0.948784	8.508081
C	6.925145	-1.557660	8.973128
C	7.249628	-1.578907	10.334278
C	7.828766	-2.148883	8.051972
C	8.434770	-2.161017	10.777888
H	6.561171	-1.128142	11.050187
C	9.014251	-2.724792	8.519410
C	9.341514	-2.743595	9.881993
H	8.644845	-2.152783	11.846868
H	9.689212	-3.171935	7.787451
C	7.512670	-2.159968	6.652112
C	7.206576	-2.157266	5.475768
C	6.785289	-2.120264	4.105042
C	5.488454	-2.543495	3.759221
C	7.610636	-1.628202	3.084174
H	4.823067	-2.925844	4.539265
C	7.109034	-1.577460	1.788438
H	8.621566	-1.287513	3.313154
C	5.806345	-2.019843	1.556645
H	7.715014	-1.198590	0.965230
H	5.386910	-1.986726	0.546645
N	5.015408	-2.496529	2.519972
C	10.652616	-3.394844	10.330758
C	10.644631	-4.875916	9.919205
H	10.557409	-4.994019	8.829185
H	11.581506	-5.358467	10.237559
H	9.805225	-5.409295	10.390614
C	11.830972	-2.681276	9.648928

H	11.855384	-1.615357	9.921983
H	12.780260	-3.139663	9.966064
H	11.771641	-2.753676	8.553127
C	10.841482	-3.310020	11.846906
H	10.874046	-2.266585	12.194545
H	10.036355	-3.830464	12.386795
H	11.793601	-3.786614	12.122699
C	-6.087860	3.895414	2.964061
C	-6.999791	4.671962	3.916563
H	-6.505690	5.574489	4.306434
H	-7.904030	4.989753	3.377022
H	-7.317179	4.054315	4.770053
C	-6.854208	2.669726	2.441151
H	-6.260980	2.100589	1.710491
H	-7.122802	1.993394	3.266848
H	-7.782252	2.992025	1.944362
C	-5.719260	4.808797	1.783999
H	-5.164771	5.693880	2.131153
H	-5.099133	4.284441	1.042277
H	-6.633723	5.152700	1.276658
C	4.575487	0.359441	5.296245
C	4.503788	0.713984	3.969159
H	5.497043	-0.054533	5.709079
C	2.208147	1.426117	4.248917
C	3.308614	1.256369	3.440931
H	5.372354	0.573670	3.321175
H	1.282900	1.838718	3.841361
H	3.258481	1.537107	2.387303

83

L_5-*syn*

C	-0.180883	-0.062207	0.072933
C	-0.022739	-0.036161	1.470065
C	0.234668	-1.249552	2.124286
C	0.309695	-2.413388	1.367673
C	0.127435	-2.326153	-0.013368
N	-0.109205	-1.176477	-0.647312
H	0.379795	-1.267820	3.205474
H	-0.368624	0.872764	-0.463665
H	0.521951	-3.374734	1.836365
H	0.180218	-3.225728	-0.633682
C	-0.082271	1.208480	2.183674
C	-0.113587	2.267106	2.779183
C	-0.102404	3.519875	3.483230
C	1.127098	4.187514	3.725441
C	-1.295105	4.090018	3.937619
C	1.106068	5.402973	4.418620
C	-1.318432	5.308343	4.631141
H	-2.225676	3.555854	3.737469
C	-0.094929	5.951145	4.862167
H	2.046685	5.921373	4.608896
H	-0.062904	6.899610	5.397026
C	2.353324	3.615147	3.262156
C	3.367408	3.086652	2.847912
C	4.482143	2.369869	2.322543
C	4.226782	1.190370	1.536722
C	5.802448	2.715497	2.522820
C	5.313748	0.409081	1.004131
C	6.878664	1.946655	1.993291
H	6.035477	3.603149	3.111654
C	6.678242	0.802751	1.247080
H	7.899639	2.273405	2.192972

C	7.735667	0.007365	0.716827
C	8.592494	-0.710549	0.237578
C	9.565582	-1.576363	-0.352525
C	10.909098	-1.196410	-0.451997
C	9.176180	-2.840674	-0.866477
C	11.846570	-2.035327	-1.049120
H	11.216156	-0.226744	-0.058131
C	10.135072	-3.664510	-1.465288
C	11.479467	-3.283625	-1.570350
H	12.880452	-1.696837	-1.105371
H	9.808257	-4.631048	-1.852675
C	7.812681	-3.274644	-0.764275
C	6.657872	-3.639720	-0.662787
C	5.297955	-4.074264	-0.531301
C	4.581047	-3.815662	0.652179
C	4.639969	-4.762945	-1.560475
H	5.073362	-3.280064	1.469349
C	3.321172	-5.155186	-1.363608
H	5.161623	-4.980859	-2.493695
C	2.705373	-4.845723	-0.150919
H	2.773311	-5.693602	-2.137203
H	1.668781	-5.146368	0.030221
N	3.319858	-4.188682	0.835023
C	12.478185	-4.233610	-2.236734
C	12.511783	-5.556790	-1.455134
H	11.526552	-6.045436	-1.441555
H	13.226683	-6.250675	-1.923463
H	12.826313	-5.389420	-0.413797
C	12.029055	-4.505113	-3.681502
H	11.988742	-3.571037	-4.262179
H	12.741122	-5.185724	-4.173008
H	11.035101	-4.974536	-3.716722
C	13.890949	-3.646461	-2.266198
H	13.927736	-2.704692	-2.834104
H	14.272997	-3.455333	-1.252235
H	14.572030	-4.359793	-2.753031
C	-2.659100	5.879211	5.100923
C	-2.485547	7.206555	5.842381
H	-2.029152	7.974653	5.200183
H	-3.470801	7.577105	6.161305
H	-1.862301	7.090990	6.741791
C	-3.332016	4.872566	6.047822
H	-3.526489	3.912368	5.548175
H	-2.701019	4.679928	6.928768
H	-4.296476	5.273102	6.395993
C	-3.561862	6.112833	3.878897
H	-3.100348	6.827793	3.180940
H	-3.756099	5.177599	3.333724
H	-4.530880	6.523304	4.202031
N	4.900959	-0.644025	0.310375
N	3.035700	0.700254	1.224207
S	3.276038	-0.638585	0.339555

83

L_5-*anti*

C	1.942821	-0.001076	1.186496
C	1.448642	-1.230885	0.715700
C	0.110003	-1.283553	0.297139
C	-0.662771	-0.131296	0.375699
C	-0.068918	1.034444	0.862536
N	1.204385	1.099978	1.256034
H	-0.305183	-2.218576	-0.083025

H	2.983694	0.069000	1.516887
H	-1.712704	-0.134479	0.074148
H	-0.652515	1.957153	0.935977
C	2.286477	-2.392890	0.672999
C	2.977710	-3.392066	0.633648
C	3.763157	-4.589473	0.567757
C	3.123900	-5.852242	0.458902
C	5.160329	-4.525307	0.596598
C	3.918051	-7.000888	0.368923
C	5.956918	-5.675300	0.513768
H	5.624740	-3.541393	0.683045
C	5.307547	-6.911842	0.395306
H	3.433356	-7.973957	0.280977
H	5.885026	-7.832637	0.323287
C	1.696145	-5.941100	0.452084
C	0.480848	-5.985618	0.462030
C	-0.945134	-5.997570	0.472357
C	-1.675331	-7.111614	-0.074869
C	-1.686945	-4.953919	0.986640
C	-3.119357	-7.104555	-0.090483
C	-3.110072	-4.946063	0.969697
H	-1.165884	-4.100506	1.421859
C	-3.850374	-5.982839	0.439953
H	-3.632043	-4.086047	1.390663
C	-5.275758	-5.960616	0.400390
C	-6.490567	-5.915836	0.366604
C	-7.918925	-5.839408	0.346156
C	-8.698322	-6.998768	0.263153
C	-8.574601	-4.583062	0.420960
C	-10.088932	-6.926010	0.263653
H	-8.200870	-7.967388	0.201510
C	-9.972895	-4.535115	0.424397
C	-10.754824	-5.695481	0.348226
H	-10.654031	-7.854944	0.198651
H	-10.449983	-3.555394	0.484878
C	-7.808195	-3.372576	0.477012
C	-7.145901	-2.353780	0.507358
C	-6.345045	-1.165355	0.532121
C	-5.017145	-1.199277	0.068116
C	-6.842099	0.055778	1.011408
H	-4.607412	-2.137104	-0.319008
C	-6.003557	1.163855	1.008895
H	-7.867370	0.123217	1.378309
C	-4.700936	1.017811	0.531749
H	-6.349545	2.131259	1.373246
H	-4.018507	1.872926	0.522812
N	-4.219807	-0.138017	0.070086
C	-12.281385	-5.576527	0.362068
C	-12.721752	-4.923492	1.682256
H	-12.296466	-3.915856	1.798483
H	-13.818559	-4.832261	1.707405
H	-12.406939	-5.530705	2.544502
C	-12.732697	-4.699880	-0.817175
H	-12.418885	-5.139642	-1.776042
H	-13.830185	-4.614451	-0.820520
H	-12.316657	-3.683834	-0.752553
C	-12.960696	-6.942791	0.242347
H	-12.692376	-7.449251	-0.696914
H	-12.696212	-7.604026	1.081067
H	-14.052287	-6.808476	0.252082
C	7.481434	-5.538698	0.556135
C	8.179198	-6.895094	0.431188

H	7.934119	-7.392363	-0.519235
H	9.268756	-6.748090	0.462633
H	7.907326	-7.570303	1.256324
C	7.891090	-4.897529	1.891916
H	7.451665	-3.896691	2.013737
H	7.568918	-5.519631	2.740741
H	8.986108	-4.793553	1.937152
C	7.941776	-4.641598	-0.604051
H	7.651280	-5.073885	-1.573619
H	7.510028	-3.632466	-0.534718
H	9.037848	-4.540871	-0.586654
N	-3.636123	-8.200334	-0.629510
N	-1.157773	-8.212303	-0.602970
S	-2.396527	-9.150275	-1.073484

16

pent

C	1.841077	-0.923398	-0.429087
H	1.643580	-0.527814	-1.430597
H	1.557120	-1.960642	-0.234647
C	2.408359	-0.166822	0.511077
H	2.585854	-0.598234	1.504632
C	2.813871	1.266315	0.333564
H	2.632377	1.585151	-0.703468
H	3.898214	1.365099	0.519643
C	2.073428	2.207872	1.286731
H	2.258036	1.910651	2.331707
H	0.987717	2.122931	1.114262
C	2.483880	3.657030	1.113773
H	3.566154	3.758928	1.322195
H	1.948628	4.281703	1.852311
O	2.181707	4.056082	-0.204689
H	2.492412	4.962056	-0.325088

16

prod

C	-1.203870	0.438258	0.545731
O	-1.285765	-0.960609	0.333728
C	-2.574624	-1.324993	-0.139093
C	-3.158924	-0.054557	-0.739132
C	-2.601250	0.999354	0.217166
H	-0.930771	0.632589	1.595649
H	-2.464016	-2.145597	-0.861909
H	-3.198743	-1.684776	0.699374
H	-2.772867	0.100026	-1.757893
H	-4.255615	-0.066259	-0.781337
H	-2.561055	2.011168	-0.208159
H	-3.204473	1.036963	1.135831
C	-0.155732	1.053593	-0.362180
H	-0.031070	2.123968	-0.165647
H	-0.384015	0.883632	-1.421282
I	1.804722	0.187886	-0.055904

23

1-H

C	1.989293	-1.156553	0.191336
C	3.374807	-1.191009	0.197167
C	4.074252	-0.000038	0.000089
C	3.376404	1.191845	-0.197192
C	1.990830	1.159268	-0.191804
N	1.346761	0.001775	-0.000330
H	5.165431	-0.000746	0.000231

H 1.366563 -2.040236 0.337413
H 3.892672 -2.136396 0.353791
H 3.895577 2.136537 -0.353676
H 1.369368 2.043798 -0.338093
C -1.995889 0.161631 1.142467
C -3.386609 0.167830 1.189905
C -4.093208 -0.001209 0.000471
C -3.387117 -0.169891 -1.189318
C -1.996374 -0.162831 -1.142612
N -1.323304 -0.000400 -0.000245
H -5.184602 -0.001451 0.000739
H -1.398749 0.290758 2.048923
H -3.899961 0.303039 2.141872
H -3.900883 -0.305303 -2.141032
H -1.399631 -0.291447 -2.049400
H 0.240347 0.001844 -0.000544

12
1-dis
C -2.927137 -0.643633 -1.003291
C -4.310343 -0.667146 -1.018715
C -5.015935 -0.034390 0.003326
C -4.316060 0.609702 1.022257
C -2.932703 0.608620 1.000797
N -2.278651 -0.012660 -0.002474
H -6.106696 -0.042962 0.005688
H -2.324588 -1.121588 -1.776500
H -4.816724 -1.182283 -1.834212
H -4.827126 1.117101 1.839680
H -2.334328 1.095166 1.771896
I -0.197593 -0.001845 -0.004019

28
1-int
C -3.443946 -0.798053 0.913895
C -4.819975 -0.625464 0.976226
C -5.410721 0.355378 0.181630
C -4.609509 1.133835 -0.651729
C -3.239859 0.907982 -0.664870
N -2.691114 -0.039311 0.106672
H -6.489813 0.512850 0.211805
H -2.929616 -1.551303 1.514551
H -5.410536 -1.255425 1.640829
H -5.031789 1.910597 -1.288610
I -0.438332 -0.352357 0.054794
C 1.947065 -1.061434 -0.425684
H 2.051625 -0.785224 -1.479247
H 1.861719 -2.122777 -0.180928
C 2.157197 -0.130316 0.559256
H 2.155666 -0.471208 1.602284
C 2.555018 1.285727 0.310689
H 2.323431 1.578804 -0.723672
H 3.658557 1.291011 0.399887
C 1.985975 2.297694 1.303145
H 2.224302 1.990694 2.333435
H 0.887980 2.329571 1.215898
C 2.531824 3.691508 1.062761
H 3.629546 3.685073 1.199939
H 2.110665 4.382940 1.814435
O 2.184657 4.082733 -0.246175
H 2.587081 4.942423 -0.423048
H -2.565296 1.488053 -1.298371

28

1-int’

C	-3.161192	-0.740745	0.767346
C	-4.529922	-0.543712	0.896428
C	-5.123230	0.513496	0.208904
C	-4.331845	1.340288	-0.586565
C	-2.969450	1.083404	-0.669405
N	-2.417112	0.064147	-0.000650
H	-6.196268	0.692166	0.292461
H	-2.645447	-1.553581	1.283454
H	-5.112665	-1.213484	1.528291
H	-4.756576	2.176982	-1.140561
I	-0.149627	-0.314724	-0.174199
C	2.169454	-0.999382	-0.781359
H	2.275422	-0.587461	-1.789401
H	2.060910	-2.081841	-0.679971
C	2.465057	-0.224962	0.315348
H	2.477765	-0.704134	1.300872
C	2.867560	1.212013	0.253834
H	2.807113	1.569864	-0.785593
H	2.161428	1.802032	0.858649
C	4.276861	1.444793	0.820638
H	4.494337	2.520942	0.766000
H	4.301049	1.160832	1.884897
C	5.355687	0.673273	0.090867
H	6.347771	0.993391	0.455505
H	5.311731	0.900991	-0.990330
O	5.142122	-0.700397	0.334528
H	5.722617	-1.214251	-0.242094
H	-2.303369	1.697875	-1.279274

28

1-ts

C	-3.267522	-0.406594	-0.654898
C	-4.630468	-0.190823	-0.832939
C	-5.316000	0.562498	0.118279
C	-4.618407	1.071845	1.211959
C	-3.256268	0.807032	1.314904
N	-2.609229	0.085524	0.396901
H	-6.385153	0.751122	0.008164
H	-2.685995	-0.989257	-1.374446
H	-5.137430	-0.608401	-1.702805
H	-5.115688	1.665986	1.978608
I	-0.175815	-0.357937	0.639789
C	2.022047	-1.027099	0.882854
H	2.167333	-1.783181	0.105654
H	2.050149	-1.387374	1.914332
C	2.561426	0.255988	0.638728
H	2.579877	0.967671	1.470046
C	2.848134	0.792705	-0.716871
H	2.757668	-0.007041	-1.466762
H	2.089256	1.562180	-0.935062
C	4.234814	1.445855	-0.758090
H	4.472386	1.766778	-1.780499
H	4.245338	2.336517	-0.111559
C	5.244269	0.439689	-0.248107
H	6.237640	0.895189	-0.115483
H	5.334808	-0.407693	-0.945917
O	4.730721	0.004149	1.004483
H	5.004202	-0.908088	1.186400
H	-2.666262	1.183032	2.154998

28
1-int$_2$

C	-3.425495	-0.251712	1.140919
C	-4.789279	-0.041888	1.340250
C	-5.499990	0.666952	0.373643
C	-4.821001	1.135852	-0.749254
C	-3.455848	0.874350	-0.856900
N	-2.774409	0.196486	0.067208
H	-6.569120	0.851261	0.493683
H	-2.835012	-0.804551	1.878138
H	-5.278062	-0.428743	2.234920
H	-5.335078	1.694550	-1.531935
I	0.031529	-0.367805	-0.322509
C	2.118490	-0.821600	-0.661726
H	2.382130	-0.420788	-1.649054
H	2.202107	-1.915307	-0.657754
C	2.961033	-0.200361	0.417819
H	2.759495	-0.635780	1.403652
C	3.058647	1.315844	0.453508
H	2.872853	1.720661	-0.552989
H	2.317152	1.733410	1.144768
C	4.495095	1.600449	0.893168
H	4.813066	2.623162	0.659240
H	4.616725	1.429807	1.971798
C	5.302964	0.592724	0.111164
H	6.251719	0.286107	0.561728
H	5.425691	0.860601	-0.945066
O	4.413079	-0.595960	0.162665
H	4.540108	-1.228799	-0.578195
H	-2.889501	1.225914	-1.724953

39
1-int$_3$

C	-3.595073	-0.374607	0.931167
C	-4.935520	-0.148057	1.242329
C	-5.627443	0.826100	0.525313
C	-4.952950	1.530918	-0.469686
C	-3.611657	1.229160	-0.703965
N	-2.946952	0.297933	-0.019929
H	-6.677727	1.032956	0.738876
H	-3.021002	-1.132141	1.474034
H	-5.420815	-0.725150	2.030135
H	-5.452185	2.302285	-1.057013
I	-0.035985	-0.240116	-0.568992
C	2.052230	-0.633486	-0.953746
H	2.324785	-0.072870	-1.857614
H	2.125709	-1.710747	-1.148348
C	2.923998	-0.223067	0.216673
H	2.629782	-0.785648	1.118323
C	2.981165	1.275450	0.509272
H	2.846269	1.839310	-0.428106
H	2.205407	1.589407	1.218227
C	4.401683	1.454387	1.040148
H	4.764257	2.487808	0.975715
H	4.463592	1.125056	2.087717
C	5.184906	0.507905	0.145467
H	6.096841	0.108188	0.608577
H	5.448659	0.984067	-0.813049
O	4.285384	-0.583480	-0.099079
H	4.688245	-1.753272	-1.122961
H	-3.050989	1.763127	-1.477645

C	5.004832	-2.294739	-3.095889
C	5.379614	-3.300438	-3.970852
C	5.724253	-4.548630	-3.450557
C	5.687123	-4.763955	-2.072488
C	5.304656	-3.720820	-1.245918
N	4.979845	-2.534598	-1.777731
H	6.022375	-5.355952	-4.121071
H	4.721261	-1.291010	-3.414243
H	5.399744	-3.103785	-5.041870
H	5.950241	-5.727570	-1.638420
H	5.246112	-3.799529	-0.160128

39

1-int$_1$-coop

C	0.038861	0.223277	-0.046389
C	0.023290	0.244070	1.342404
C	1.180168	-0.115958	2.031203
C	2.315053	-0.486388	1.312093
C	2.256911	-0.484563	-0.075803
N	1.137842	-0.134967	-0.720419
H	1.196866	-0.108193	3.122123
H	-0.839675	0.496412	-0.635518
H	-0.885323	0.539052	1.866796
H	3.239122	-0.774898	1.812505
I	1.094314	-0.139629	-3.037935
C	1.150916	0.311732	-5.469868
H	2.171895	0.676557	-5.617623
H	0.339165	1.041560	-5.516350
C	0.874366	-1.035550	-5.569006
H	-0.173809	-1.351687	-5.542968
C	1.900557	-2.107056	-5.736727
H	2.897843	-1.652720	-5.847099
H	1.911457	-2.718681	-4.819934
C	1.584938	-3.035453	-6.916053
H	2.354167	-3.820132	-6.965155
H	0.618659	-3.535125	-6.738951
C	1.511234	-2.284689	-8.233616
H	1.341507	-3.003579	-9.055194
H	2.481998	-1.793799	-8.433322
O	0.472823	-1.339926	-8.183348
H	0.862624	-0.457639	-8.384597
H	3.116812	-0.766943	-0.687508
C	0.994957	2.182615	-8.270771
C	1.550589	3.444828	-8.072292
C	2.938371	3.570508	-8.095265
C	3.712140	2.433650	-8.320801
C	3.063838	1.214503	-8.510263
N	1.734668	1.091367	-8.482626
H	3.410920	4.542348	-7.941936
H	-0.089906	2.042611	-8.255016
H	0.904715	4.306826	-7.903741
H	4.800771	2.484200	-8.351942
H	3.638046	0.301462	-8.690489

39

1-ts-coop

C	2.223995	-0.606638	-0.107127
C	2.271576	-0.620925	1.282302
C	1.111932	-0.318127	1.993575
C	-0.054738	-0.011721	1.295497
C	-0.021292	-0.018942	-0.094417
N	1.097974	-0.311488	-0.762871

H	1.117336	-0.320964	3.084740
H	3.104900	-0.837268	-0.711638
H	3.204439	-0.865904	1.789838
H	-0.982546	0.230398	1.813564
I	1.084252	-0.312407	-3.161604
C	0.989409	0.035183	-5.520391
H	1.723286	0.827577	-5.690687
H	-0.064270	0.296392	-5.643970
C	1.377747	-1.292647	-5.689300
H	0.603253	-2.064625	-5.657853
C	2.789886	-1.750468	-5.805027
H	3.462379	-0.880497	-5.860792
H	3.038866	-2.315062	-4.891400
C	2.980330	-2.679940	-7.010180
H	4.036690	-2.973958	-7.084664
H	2.389356	-3.597177	-6.859059
C	2.507349	-1.992602	-8.278112
H	2.593303	-2.682056	-9.136204
H	3.147814	-1.118469	-8.495946
O	1.171757	-1.600246	-8.081175
H	1.071240	-0.651934	-8.362075
H	-0.908173	0.214080	-0.689167
C	-0.120425	1.467051	-9.351535
C	-0.310780	2.804297	-9.693209
C	0.700294	3.716172	-9.397730
C	1.859213	3.257264	-8.774111
C	1.957532	1.901552	-8.470267
N	0.988440	1.027173	-8.752486
H	0.587227	4.771761	-9.651041
H	-0.888776	0.719854	-9.568294
H	-1.233727	3.117398	-10.181815
H	2.677280	3.933873	-8.526325
H	2.851794	1.504038	-7.981652

39

1-int$_2$-coop

C	0.206459	-0.706105	0.015708
C	0.178483	-0.799531	1.406905
C	1.213541	-0.212454	2.132006
C	2.230254	0.441306	1.438663
C	2.166236	0.478992	0.046027
N	1.177388	-0.081296	-0.650407
H	1.227733	-0.263734	3.222267
H	-0.589783	-1.155971	-0.585801
H	-0.638736	-1.322007	1.905203
H	3.060833	0.915713	1.962319
I	1.148409	-0.030022	-3.658521
C	1.127800	-0.005186	-5.817934
H	2.147669	-0.237152	-6.152046
H	0.865947	1.020193	-6.107667
C	0.137925	-1.006069	-6.380132
H	-0.879924	-0.762373	-6.032629
C	0.459885	-2.476818	-6.120781
H	1.552233	-2.609820	-6.058672
H	0.012310	-2.840498	-5.187898
C	-0.090957	-3.160803	-7.369968
H	0.342239	-4.152351	-7.551013
H	-1.183989	-3.262293	-7.301076
C	0.275890	-2.160317	-8.453390
H	-0.391801	-2.180992	-9.324938
H	1.315053	-2.297883	-8.794671
O	0.151142	-0.879516	-7.817667

H	0.583085	0.453831	-8.603071
H	2.947934	0.983413	-0.530669
C	-0.129660	2.133592	-9.572242
C	0.183597	3.268405	-10.301473
C	1.523291	3.533196	-10.587926
C	2.517131	2.661350	-10.141133
C	2.144471	1.542273	-9.415672
N	0.848956	1.317701	-9.157360
H	1.793728	4.421482	-11.160876
H	-1.147512	1.848833	-9.304706
H	-0.613117	3.930455	-10.637836
H	3.570487	2.842762	-10.350375
H	2.853612	0.809691	-9.029132

35
2-H

C	0.674848	-5.831526	-0.026935
C	1.382554	-4.633198	-0.045812
C	0.691688	-3.414964	-0.023033
C	-0.728472	-3.405109	0.018247
C	-1.422513	-4.620831	0.038145
C	-0.721484	-5.824127	0.016015
H	1.216003	-6.778202	-0.044992
H	-2.512378	-4.612490	0.070114
H	-1.270747	-6.766387	0.031618
C	-1.395340	-2.139184	0.031789
C	-1.829700	-1.005372	0.029780
C	-1.865484	2.591713	-0.102261
C	-3.219095	2.926510	-0.078067
C	-4.155544	1.900120	-0.004176
C	-3.705387	0.584953	0.036294
C	-2.325791	0.343702	0.005535
N	-1.418622	1.337016	-0.058152
H	-5.224191	2.118043	0.019350
H	-1.114381	3.382006	-0.162642
H	-3.520597	3.973020	-0.116415
H	-4.397408	-0.255202	0.089223
C	1.368622	-2.157070	-0.033808
C	1.823224	-1.031387	-0.031447
C	1.839855	2.604755	0.065868
C	3.184502	2.934201	0.074523
C	4.124055	1.904027	0.037764
C	3.700780	0.579567	-0.000934
C	2.333270	0.297943	-0.005489
N	1.449981	1.324275	0.024549
H	5.190719	2.131955	0.040729
H	1.048414	3.352690	0.091328
H	3.481578	3.981058	0.108176
H	4.408386	-0.247788	-0.026322
H	2.472517	-4.629180	-0.077598
H	0.404781	1.138905	0.001662

35
2-dis$_a$

I	2.301064	-0.355534	3.103957
N	-0.539405	0.117869	1.238407
C	-1.558939	-0.661760	1.583359
C	-0.724113	1.448405	1.271852
C	-2.805967	-0.169863	1.977546
H	-1.381724	-1.741671	1.546267
C	-1.935526	2.040092	1.653102
C	0.410448	2.250604	0.888252

C	-2.992997	1.209059	2.012083
H	-3.606024	-0.858773	2.249003
H	-2.032401	3.125681	1.662443
C	1.411746	2.847129	0.546731
H	-3.950695	1.635578	2.314184
C	2.667857	3.369333	0.097792
C	2.926531	4.741885	0.004840
C	3.681463	2.446906	-0.281032
C	4.162759	5.191091	-0.454007
H	2.149548	5.450307	0.293302
C	4.917129	2.912307	-0.744738
C	3.383595	1.052224	-0.160680
C	5.154138	4.281851	-0.828536
H	4.353869	6.262956	-0.521748
H	5.684963	2.194196	-1.033739
C	2.988773	-0.081089	0.025435
H	6.118608	4.641856	-1.188487
C	2.409163	-1.358900	0.263319
C	2.204388	-2.281824	-0.764216
N	2.004392	-1.681349	1.525437
C	1.580048	-3.493775	-0.496034
H	2.534195	-2.015749	-1.767474
C	1.393022	-2.849593	1.801054
C	1.164719	-3.779664	0.803074
H	1.415350	-4.211128	-1.300672
H	1.096359	-3.023938	2.835688
H	0.667748	-4.714671	1.058777

35

2-dis$_b$

I	0.245844	-0.774079	1.764522
N	-2.305068	1.899376	0.329134
C	-3.438159	1.345278	0.752017
C	-1.309710	2.047232	1.209901
C	-3.637977	0.892836	2.059182
H	-4.235941	1.245010	0.010558
C	-1.419922	1.658984	2.560476
C	-0.051947	2.556156	0.714618
C	-2.610169	1.060487	2.981883
H	-4.584050	0.430593	2.340665
H	-0.603942	1.859664	3.257515
C	1.045229	2.882333	0.309931
H	-2.728189	0.742810	4.019207
C	2.338935	3.263591	-0.179688
C	2.609114	4.603010	-0.488451
C	3.356478	2.293333	-0.364167
C	3.861923	4.977506	-0.966834
H	1.824092	5.346629	-0.348519
C	4.613314	2.686558	-0.850211
C	3.123783	0.913643	-0.065700
C	4.863322	4.021712	-1.147593
H	4.057011	6.024884	-1.201017
H	5.388238	1.931910	-0.988770
C	2.947896	-0.263461	0.183523
H	5.843540	4.317993	-1.522734
C	2.812419	-1.657064	0.444940
C	3.784950	-2.569065	0.021311
N	1.724082	-2.124410	1.120069
C	3.630682	-3.922304	0.290722
H	4.650011	-2.186083	-0.518582
C	1.561738	-3.434434	1.389687
C	2.503870	-4.361960	0.985482

H	4.387968	-4.633231	-0.041362
H	0.661517	-3.721568	1.934462
H	2.343196	-5.413820	1.218366

35

2-dis$_c$

I	-0.019745	-0.952313	1.006347
N	-1.624370	2.382697	2.441455
C	-2.724136	1.866935	2.983731
C	-1.324482	2.043468	1.182897
C	-3.571888	0.977391	2.317748
H	-2.946625	2.169025	4.011035
C	-2.129857	1.181947	0.410944
C	-0.079987	2.534598	0.638168
C	-3.270717	0.634577	1.003488
H	-4.450966	0.576069	2.821837
H	-1.879757	0.986524	-0.633725
C	1.002214	2.849965	0.187158
H	-3.915031	-0.039200	0.436240
C	2.285690	3.225395	-0.332875
C	2.523597	4.545292	-0.737520
C	3.330606	2.272392	-0.435984
C	3.769136	4.915965	-1.237321
H	1.719371	5.276890	-0.654729
C	4.581483	2.662497	-0.939756
C	3.135626	0.916005	-0.025701
C	4.797151	3.976828	-1.339093
H	3.938182	5.947622	-1.548724
H	5.378476	1.921671	-1.012799
C	2.994067	-0.241267	0.319693
H	5.772021	4.270408	-1.729924
C	2.912659	-1.604343	0.723764
C	4.052266	-2.415002	0.759956
N	1.713551	-2.140878	1.088613
C	3.949849	-3.737465	1.169981
H	5.004986	-1.977176	0.465322
C	1.599718	-3.421961	1.490118
C	2.706772	-4.248427	1.543160
H	4.838898	-4.368423	1.199500
H	0.603817	-3.769018	1.767749
H	2.581502	-5.278642	1.873788

35

2-dis$_d$

I	2.301064	-0.355534	3.103957
N	-0.539405	0.117869	1.238407
C	-1.558939	-0.661760	1.583359
C	-0.724113	1.448405	1.271852
C	-2.805967	-0.169863	1.977546
H	-1.381724	-1.741671	1.546267
C	-1.935526	2.040092	1.653102
C	0.410448	2.250604	0.888252
C	-2.992997	1.209059	2.012083
H	-3.606024	-0.858773	2.249003
H	-2.032401	3.125681	1.662443
C	1.411746	2.847129	0.546731
H	-3.950695	1.635578	2.314184
C	2.667857	3.369333	0.097792
C	2.926531	4.741885	0.004840
C	3.681463	2.446906	-0.281032
C	4.162759	5.191091	-0.454007
H	2.149548	5.450307	0.293302

C	4.917129	2.912307	-0.744738
C	3.383595	1.052224	-0.160680
C	5.154138	4.281851	-0.828536
H	4.353869	6.262956	-0.521748
H	5.684963	2.194196	-1.033739
C	2.988773	-0.081089	0.025435
H	6.118608	4.641856	-1.188487
C	2.409163	-1.358900	0.263319
C	2.204388	-2.281824	-0.764216
N	2.004392	-1.681349	1.525437
C	1.580048	-3.493775	-0.496034
H	2.534195	-2.015749	-1.767474
C	1.393022	-2.849593	1.801054
C	1.164719	-3.779664	0.803074
H	1.415350	-4.211128	-1.300672
H	1.096359	-3.023938	2.835688
H	0.667748	-4.714671	1.058777

51

2-int$_{1a}$

H	9.524745	-1.321936	-3.376485
O	9.093071	-1.366981	-2.513071
C	9.696147	-0.429034	-1.647164
H	9.762830	0.566825	-2.121871
H	10.721219	-0.736896	-1.375534
C	8.841558	-0.342561	-0.400383
H	9.314379	0.336579	0.323653
H	8.790223	-1.339003	0.070278
C	7.420118	0.156509	-0.683556
H	7.430384	1.174384	-1.103445
H	6.883703	0.201347	0.278242
C	6.670340	-0.758519	-1.598509
H	6.731502	-1.832085	-1.387909
C	5.983304	-0.353994	-2.727965
H	6.025104	0.690744	-3.049053
H	5.624930	-1.095703	-3.445725
I	4.154428	-0.417192	-1.097010
N	2.157162	-0.345057	0.173201
C	0.993752	-0.519678	-0.455393
C	-0.220365	-0.476930	0.221531
C	-0.209004	-0.243710	1.594374
C	1.007071	-0.067187	2.246569
C	2.191292	-0.127168	1.504355
H	1.037737	-0.698141	-1.532646
H	-1.150182	-0.624321	-0.326847
H	-1.141959	-0.202545	2.157913
H	1.061499	0.111372	3.319906
C	3.473013	0.026418	2.133630
C	4.568771	0.120046	2.648291
C	5.900819	0.160035	3.178211
C	6.813756	-0.868129	2.822908
C	8.125951	-0.820977	3.312628
C	8.527615	0.221883	4.143532
C	7.626563	1.227854	4.498212
C	6.319298	1.198824	4.019788
H	8.823604	-1.613399	3.039113
H	9.550954	0.247680	4.520301
H	7.944214	2.041534	5.151416
H	5.611613	1.983407	4.289215
C	6.367734	-1.928054	1.968548
C	5.892463	-2.779630	1.244220
C	5.270528	-3.739219	0.365548

C	6.051077	-4.586058	-0.434016
C	5.399797	-5.466280	-1.294116
C	4.008170	-5.470176	-1.322221
C	3.322645	-4.588554	-0.481425
N	3.928826	-3.740690	0.342336
H	5.973967	-6.139225	-1.932883
H	3.455523	-6.141801	-1.979632
H	2.228506	-4.571421	-0.480765
H	7.139104	-4.542907	-0.374850

51

2-ts$_a$

H	-0.190368	-0.101584	0.077423
O	-0.077112	-0.065717	1.039496
C	-0.657026	-1.208793	1.655819
H	-0.586571	-2.084021	0.991360
H	-1.718104	-1.022905	1.882803
C	0.151820	-1.432872	2.917027
H	-0.121201	-2.378725	3.403045
H	-0.039745	-0.616385	3.631702
C	1.634034	-1.439010	2.534123
H	1.890294	-2.305980	1.906805
H	2.238912	-1.489893	3.454633
C	1.990835	-0.177019	1.836034
H	1.742573	0.761368	2.342577
C	2.833201	-0.104718	0.696173
H	2.947784	-1.015537	0.101379
H	2.833651	0.836556	0.140740
I	4.683538	0.049041	2.024856
N	6.888172	0.225329	3.341567
C	7.918361	0.857787	2.785302
C	9.150649	0.986449	3.423394
C	9.302834	0.427160	4.689195
C	8.226438	-0.233219	5.273609
C	7.020697	-0.313332	4.566249
H	7.755447	1.277728	1.788697
H	9.966999	1.513144	2.929747
H	10.251981	0.504852	5.221259
H	8.299414	-0.681296	6.264130
C	5.873660	-0.970250	5.138306
C	4.892495	-1.496454	5.622965
C	3.679660	-2.047042	6.157071
C	2.497403	-1.259802	6.148115
C	1.305445	-1.800370	6.649550
C	1.281609	-3.097062	7.156440
C	2.445565	-3.868163	7.171286
C	3.638344	-3.347861	6.675869
H	0.401176	-1.190479	6.642442
H	0.349073	-3.507287	7.546190
H	2.424573	-4.882793	7.571102
H	4.549783	-3.946547	6.682195
C	2.558906	0.067623	5.612775
C	2.734239	1.167127	5.126807
C	3.042707	2.435850	4.514646
C	2.030195	3.241619	3.975782
C	2.395889	4.433312	3.354940
C	3.744208	4.773977	3.298275
C	4.675495	3.904055	3.873191
N	4.343066	2.763061	4.466211
H	1.635831	5.085958	2.922527
H	4.077330	5.696114	2.821460
H	5.742072	4.147152	3.847246

H 0.987629 2.929854 4.047509

51

2-int$_{2a}$

H	0.286071	0.098003	0.244928
O	0.238436	-0.101092	1.205777
C	-0.569311	-1.306916	1.536295
H	-0.472535	-2.000141	0.692331
H	-1.600491	-0.961353	1.655405
C	0.113480	-1.798485	2.792165
H	-0.093992	-2.862290	2.957694
H	-0.236864	-1.229800	3.665305
C	1.590609	-1.519079	2.520133
H	2.001418	-2.245763	1.802210
H	2.201202	-1.538555	3.431710
C	1.596253	-0.131597	1.906743
H	1.495481	0.657308	2.662765
C	2.658694	0.193027	0.892888
H	2.716168	-0.574820	0.110468
H	2.504296	1.184616	0.449564
I	4.566198	0.242725	1.902874
N	7.104637	0.198144	3.393903
C	8.208048	0.712238	2.859982
C	9.464145	0.614911	3.460859
C	9.565520	-0.056408	4.676356
C	8.416586	-0.597957	5.244702
C	7.198591	-0.444206	4.567600
H	8.090786	1.228729	1.902612
H	10.337025	1.056675	2.979927
H	10.528141	-0.157801	5.179858
H	8.445297	-1.129336	6.195864
C	5.983443	-0.981850	5.131986
C	4.965998	-1.428088	5.622995
C	3.743466	-1.932202	6.180190
C	2.573127	-1.129659	6.171676
C	1.381238	-1.643011	6.704029
C	1.343787	-2.927119	7.239473
C	2.496673	-3.714970	7.252944
C	3.687932	-3.221685	6.728664
H	0.486506	-1.019079	6.697940
H	0.411146	-3.313935	7.652278
H	2.468177	-4.720637	7.674517
H	4.590752	-3.833416	6.734638
C	2.624140	0.188590	5.611772
C	2.737991	1.293011	5.118279
C	2.920790	2.579301	4.490663
C	1.830499	3.257108	3.925736
C	2.066059	4.470766	3.285437
C	3.367553	4.961742	3.237613
C	4.382124	4.215368	3.843839
N	4.173612	3.054675	4.454820
H	1.242520	5.024756	2.832075
H	3.600856	5.906549	2.746065
H	5.413551	4.579916	3.829426
H	0.828031	2.833092	3.994072

51

2-int$_{1b}$

I	0.375849	-0.667921	-1.341177
C	-1.433447	0.127817	-2.695439
C	-2.097956	-0.126870	-1.502542
H	-1.097132	1.141051	-2.926959

H	-1.501472	-0.590066	-3.518254
C	-2.884024	-1.364586	-1.224924
H	-2.163208	0.681238	-0.762759
C	-4.381359	-1.047920	-1.070588
H	-2.531516	-1.806849	-0.280019
H	-2.723612	-2.100168	-2.027995
C	-5.013389	-0.487316	-2.326870
H	-4.897160	-1.974919	-0.780682
H	-4.518164	-0.325374	-0.250315
O	-4.416843	0.765557	-2.584677
H	-4.858785	-1.186511	-3.169555
H	-6.102351	-0.387154	-2.174571
H	-4.749748	1.106256	-3.425473
N	-3.039258	-0.309672	2.106492
C	-3.686960	-1.456311	2.298197
C	-1.699567	-0.342396	2.077717
C	-3.045603	-2.687013	2.456722
H	-4.779358	-1.400522	2.322413
C	-0.961081	-1.528724	2.218006
C	-1.003441	0.907078	1.891000
C	-1.653311	-2.720089	2.410192
H	-3.630460	-3.593714	2.612976
H	0.128767	-1.498842	2.181328
C	-0.369303	1.932482	1.741359
H	-1.112226	-3.660394	2.526877
C	0.410562	3.124778	1.586777
C	-0.153457	4.384917	1.832226
C	1.772935	3.038387	1.201830
C	0.616271	5.537449	1.704584
H	-1.201015	4.447159	2.128692
C	2.536029	4.208286	1.082873
C	2.352415	1.758423	0.918674
C	1.959474	5.449960	1.332897
H	0.164967	6.511301	1.899324
H	3.583084	4.131279	0.787892
C	2.796847	0.660323	0.652618
H	2.561204	6.354527	1.236210
C	3.291204	-0.646740	0.320845
C	4.539294	-1.087588	0.774998
N	2.511484	-1.439476	-0.441620
C	4.968371	-2.365314	0.429637
H	5.147997	-0.425451	1.389874
C	2.921268	-2.664194	-0.772388
C	4.148267	-3.171559	-0.355584
H	5.937805	-2.727976	0.773851
H	2.246411	-3.257757	-1.394418
H	4.445377	-4.178326	-0.647723

51

2-ts$_b$

I	-0.336873	-1.403953	-0.586197
C	-2.089089	-0.687866	-1.853841
C	-2.979377	-0.807218	-0.757467
H	-1.792341	0.323518	-2.143180
H	-2.196584	-1.410847	-2.667950
C	-3.741293	-2.037117	-0.429151
H	-3.007140	0.009354	-0.027611
C	-5.216876	-1.696787	-0.175159
H	-3.315954	-2.459938	0.497184
H	-3.628564	-2.782364	-1.230355
C	-5.765266	-0.975243	-1.386604
H	-5.781397	-2.614360	0.037683

H	-5.289466	-1.038453	0.704034
O	-4.880307	0.114765	-1.597707
H	-5.780779	-1.641678	-2.264047
H	-6.788916	-0.611959	-1.204285
H	-4.886474	0.383026	-2.528841
N	-3.775378	-1.084658	2.931222
C	-4.397328	-2.243092	3.138794
C	-2.437433	-1.095703	2.849172
C	-3.731692	-3.464932	3.258922
H	-5.488619	-2.204543	3.207285
C	-1.675936	-2.271666	2.951053
C	-1.765931	0.163648	2.642951
C	-2.341712	-3.475234	3.157673
H	-4.295856	-4.382201	3.428997
H	-0.589246	-2.224407	2.870355
C	-1.143799	1.193847	2.476119
H	-1.781815	-4.407876	3.243046
C	-0.375767	2.390613	2.299307
C	-0.967455	3.650785	2.469033
C	1.001371	2.309019	1.968703
C	-0.211469	4.809618	2.319828
H	-2.026270	3.708159	2.723799
C	1.749653	3.486187	1.827573
C	1.612193	1.028078	1.763951
C	1.145838	4.727901	2.001857
H	-0.684138	5.783332	2.455524
H	2.808189	3.414254	1.575277
C	2.087150	-0.071197	1.563183
H	1.737439	5.637183	1.887793
C	2.607559	-1.387061	1.301287
C	3.847341	-1.791454	1.810809
N	1.852065	-2.210305	0.552095
C	4.296521	-3.078981	1.532373
H	4.433113	-1.098136	2.413708
C	2.284391	-3.440860	0.287663
C	3.503839	-3.923478	0.759459
H	5.258806	-3.418574	1.917986
H	1.633051	-4.066635	-0.328956
H	3.817220	-4.939112	0.518853

51

2-int$_{2b}$

I	0.333155	-0.698261	-1.511233
C	-1.375655	0.063871	-2.584015
C	-2.563347	0.080455	-1.660570
H	-1.105484	1.076494	-2.908384
H	-1.550784	-0.584549	-3.452080
C	-3.184096	-1.246922	-1.270246
H	-2.409698	0.739886	-0.795837
C	-4.662517	-0.913719	-1.075802
H	-2.712742	-1.644684	-0.362673
H	-3.048676	-1.974352	-2.086015
C	-4.976362	-0.034458	-2.264416
H	-5.303828	-1.803111	-1.065829
H	-4.808432	-0.354704	-0.140716
O	-3.719130	0.751089	-2.397949
H	-5.104769	-0.595806	-3.197173
H	-5.778888	0.694757	-2.120935
H	-3.510109	1.009921	-3.322305
N	-3.106176	-0.389372	2.059082
C	-3.732967	-1.544623	2.270774
C	-1.770198	-0.411895	1.945456

C	-3.075328	-2.773011	2.364806
H	-4.821964	-1.497776	2.366439
C	-1.016945	-1.595303	2.015620
C	-1.091990	0.843846	1.741470
C	-1.688479	-2.794568	2.228337
H	-3.643305	-3.686876	2.540528
H	0.066794	-1.556709	1.899142
C	-0.465268	1.871907	1.578794
H	-1.134965	-3.732927	2.289144
C	0.301728	3.069097	1.403479
C	-0.309342	4.327330	1.511789
C	1.692866	2.991580	1.136903
C	0.439623	5.490369	1.363216
H	-1.378717	4.379224	1.719585
C	2.432886	4.174621	0.994710
C	2.332992	1.715273	1.003992
C	1.810272	5.414106	1.106579
H	-0.048003	6.462097	1.451916
H	3.502370	4.108062	0.791756
C	2.851907	0.625559	0.868262
H	2.397895	6.326074	0.992777
C	3.449290	-0.675564	0.692162
C	4.741021	-0.935865	1.169916
N	2.714005	-1.603386	0.059545
C	5.273548	-2.207931	0.983068
H	5.300972	-0.149800	1.676341
C	3.233182	-2.815133	-0.113733
C	4.507772	-3.170706	0.330912
H	6.275949	-2.442847	1.344276
H	2.605577	-3.543735	-0.635427
H	4.884296	-4.180038	0.164109

51

2-int$_{1c}$

C	0.394171	-0.698591	-1.263908
C	-0.650687	-0.261148	-2.073748
C	-1.936722	-0.190760	-1.548018
C	-2.145966	-0.563963	-0.215663
N	-1.123661	-0.992064	0.551284
C	0.109900	-1.057565	0.049912
C	-3.452337	-0.498454	0.376500
C	-4.546848	-0.436390	0.897904
C	-5.808036	-0.365995	1.574637
C	-5.870224	0.174106	2.885001
C	-7.104323	0.218851	3.549226
C	-8.255712	-0.256644	2.928426
C	-8.193722	-0.780538	1.635466
C	-6.977643	-0.835067	0.960918
C	-4.682737	0.684948	3.503394
C	-3.657169	1.131157	3.977870
C	-2.434049	1.669303	4.521174
C	-2.368424	2.057028	5.867777
C	-1.171860	2.582774	6.348131
C	-0.092693	2.699388	5.476380
C	-0.258890	2.280242	4.152568
N	-1.394077	1.775820	3.679515
I	-1.447885	-1.757873	2.829376
C	-1.690161	-3.047563	4.844308
C	-1.678305	-1.755993	5.351473
C	-0.472841	-1.092572	5.933041
C	-0.636938	-0.841868	7.440652
C	-0.823818	-2.108937	8.247979

O	-2.062500	-2.674335	7.875305
H	-2.639162	-3.556787	4.662064
H	-0.782493	-3.656458	4.889963
H	-2.636577	-1.232037	5.454560
H	-0.329394	-0.118495	5.440385
H	0.420335	-1.707323	5.741015
H	0.255090	-0.305158	7.795126
H	-1.503329	-0.183701	7.614417
H	0.011453	-2.806236	8.051185
H	-0.804619	-1.864945	9.324711
H	-2.149762	-3.542008	8.291764
H	-1.086339	2.896550	7.389844
H	0.863122	3.107222	5.806079
H	0.571428	2.359403	3.444430
H	-7.149299	0.635226	4.556094
H	-9.209264	-0.214961	3.456516
H	-6.919985	-1.245077	-0.047910
H	-9.097887	-1.149510	1.149622
H	-2.781953	0.150650	-2.144682
H	-0.467362	0.026006	-3.109983
H	0.895702	-1.411841	0.721796
H	1.416394	-0.766851	-1.634686
H	-3.243330	1.948973	6.509407

51

2-ts$_c$

C	-0.052244	-0.046960	0.037663
C	-0.044477	-0.051107	1.429997
C	1.140196	-0.337608	2.101347
C	2.289164	-0.613316	1.349977
N	2.268515	-0.612496	0.005746
C	1.134427	-0.337017	-0.633253
C	3.543643	-0.904002	1.992497
C	4.618476	-1.139687	2.505180
C	5.918385	-1.401578	3.050068
C	7.049791	-0.726619	2.523379
C	8.320027	-1.003260	3.049728
C	8.470207	-1.927492	4.079280
C	7.355082	-2.586972	4.600355
C	6.086675	-2.326018	4.090275
C	6.882839	0.236968	1.475891
C	6.690630	1.057677	0.600901
C	6.447641	2.033289	-0.433225
C	7.523651	2.652448	-1.086737
C	7.245751	3.590378	-2.077712
C	5.916587	3.871998	-2.381081
C	4.916014	3.200664	-1.670695
N	5.165326	2.304159	-0.721820
I	4.356095	-1.251844	-1.397336
C	6.038810	-2.078550	-2.685051
C	6.723232	-0.835374	-2.675145
C	6.483937	0.254062	-3.656685
C	7.812171	0.758867	-4.237005
C	8.576997	-0.425643	-4.785449
O	8.641314	-1.346441	-3.704934
H	6.463858	-2.880529	-2.075846
H	5.581022	-2.385173	-3.630124
H	7.337868	-0.597306	-1.800425
H	5.995872	1.085236	-3.120155
H	5.807028	-0.093076	-4.451559
H	7.621257	1.512513	-5.012303
H	8.405022	1.234114	-3.440758

H	8.049655	-0.869197	-5.644881
H	9.590406	-0.137011	-5.104968
H	8.714916	-2.255382	-4.034164
H	8.058510	4.092651	-2.605047
H	5.650028	4.599068	-3.148479
H	3.862265	3.404117	-1.883759
H	9.186865	-0.480884	2.643470
H	9.463931	-2.132373	4.479926
H	5.211470	-2.839099	4.490318
H	7.474025	-3.309660	5.408728
H	1.193576	-0.348313	3.189633
H	-0.953407	0.168711	1.991887
H	1.171640	-0.350119	-1.726221
H	-0.957502	0.174155	-0.527419
H	8.547704	2.399031	-0.810651

51

2-int$_{2c}$

C	0.337932	-0.888769	-1.465725
C	-0.675960	-0.193339	-2.119210
C	-1.900933	-0.028081	-1.479767
C	-2.063411	-0.570815	-0.197804
N	-1.086472	-1.243167	0.428460
C	0.079510	-1.395299	-0.190806
C	-3.313643	-0.426540	0.507705
C	-4.358897	-0.321576	1.116553
C	-5.584017	-0.225276	1.855954
C	-5.570803	0.165415	3.219161
C	-6.781338	0.234514	3.925860
C	-7.984310	-0.073688	3.298726
C	-7.997198	-0.453119	1.954791
C	-6.806602	-0.527611	1.238527
C	-4.336595	0.505007	3.863493
C	-3.283425	0.809600	4.387230
C	-2.038814	1.191065	5.008384
C	-1.996580	1.473244	6.382826
C	-0.781701	1.858743	6.943078
C	0.339841	1.941745	6.121999
C	0.193531	1.636514	4.765434
N	-0.959734	1.269254	4.215799
I	-1.585603	-2.495255	3.057305
C	-1.956714	-3.426538	4.968363
C	-1.837986	-2.391480	6.054047
C	-0.455360	-1.825182	6.335510
C	-0.404391	-1.650904	7.853804
C	-1.069235	-2.905160	8.367529
O	-2.168924	-3.065233	7.384141
H	-2.970192	-3.843688	4.919394
H	-1.217716	-4.227858	5.100243
H	-2.608734	-1.614066	5.971313
H	-0.309266	-0.881419	5.795743
H	0.311047	-2.541169	6.001508
H	0.620140	-1.563329	8.234382
H	-0.976040	-0.765563	8.165745
H	-0.433334	-3.795712	8.294911
H	-1.544041	-2.828522	9.350266
H	-2.516940	-3.980526	7.303886
H	-0.714217	2.092111	8.007117
H	1.311636	2.241464	6.514918
H	1.055760	1.694444	4.094460
H	-6.765382	0.538965	4.972988
H	-8.917551	-0.014407	3.860418

H	-6.808998	-0.825414	0.189466
H	-8.940229	-0.692255	1.461328
H	-2.723809	0.509590	-1.950506
H	-0.517144	0.217867	-3.117299
H	0.850791	-1.949769	0.352249
H	1.313003	-1.041458	-1.928724
H	-2.904485	1.399269	6.982267

51

2-int$_{1d}$

C	0.034764	-0.102592	0.018956
C	0.032627	-0.090420	1.412365
C	1.237066	0.019986	2.098822
C	2.426665	0.116954	1.364487
N	2.408073	0.105171	0.016324
C	1.252184	-0.001375	-0.643666
C	3.679185	0.226038	2.051350
C	4.684455	0.308675	2.727743
C	5.834750	0.400568	3.573087
C	5.664246	0.390419	4.981770
C	6.793250	0.477138	5.808454
C	8.065863	0.572966	5.253334
C	8.231729	0.583946	3.866360
C	7.123508	0.498123	3.029698
C	4.349161	0.292701	5.541052
C	3.218588	0.209442	5.977650
C	1.874514	0.112742	6.490113
N	0.884032	0.034725	5.588423
C	-0.364601	-0.053011	6.035254
C	-0.696610	-0.068091	7.391733
C	0.332403	0.012128	8.325998
C	1.644908	0.104221	7.873352
I	4.365922	0.278580	-1.290483
C	6.167026	0.930961	-2.819306
C	6.583894	-0.351055	-2.521596
C	7.603109	-0.686760	-1.482770
C	8.828238	-1.390050	-2.088690
C	9.532455	-0.564707	-3.144237
O	8.647314	-0.422059	-4.234618
H	-1.150574	-0.115273	5.276701
H	-1.739848	-0.141452	7.699496
H	2.484839	0.169103	8.565022
H	0.118037	0.003288	9.395766
H	6.658992	0.468310	6.890534
H	8.935744	0.639256	5.908209
H	7.242641	0.505751	1.945728
H	9.230910	0.659928	3.435649
H	1.277331	0.032645	3.191368
H	-0.904578	-0.167163	1.965586
H	1.307611	-0.006426	-1.734932
H	-0.886749	-0.188240	-0.555856
H	5.583188	1.124393	-3.722369
H	6.630020	1.785283	-2.316228
H	6.212724	-1.174468	-3.141541
H	7.150313	-1.372627	-0.750056
H	7.905560	0.229701	-0.952720
H	9.528190	-1.622126	-1.273641
H	8.520452	-2.347758	-2.537766
H	10.463489	-1.073630	-3.450088
H	9.814517	0.418788	-2.725301
H	9.009270	0.227352	-4.851961

51
2-ts$_d$

C	0.003756	-0.015958	-0.006713
C	0.001046	-0.010978	1.386361
C	1.206637	0.107867	2.070558
C	2.389910	0.219474	1.326754
N	2.380485	0.211839	-0.017524
C	1.224207	0.098117	-0.668105
C	3.648548	0.345175	2.007163
C	4.670150	0.449141	2.655342
C	5.846176	0.568539	3.461831
C	5.730834	0.534248	4.875557
C	6.886967	0.649660	5.660626
C	8.133606	0.797484	5.060039
C	8.245521	0.833156	3.668050
C	7.109582	0.719358	2.872888
C	4.440704	0.385325	5.480183
C	3.326930	0.260285	5.948874
C	2.002070	0.113958	6.498323
N	0.985650	0.044575	5.625266
C	-0.245802	-0.089387	6.106967
C	-0.534328	-0.161164	7.471578
C	0.521591	-0.089295	8.376048
C	1.816543	0.050868	7.886812
I	4.480733	0.384569	-1.435703
C	6.239375	0.774475	-2.857843
C	6.805228	-0.500608	-2.623835
C	7.758171	-0.802653	-1.525873
C	8.951612	-1.611118	-2.051943
C	9.575933	-0.852748	-3.203075
O	8.505322	-0.589762	-4.098803
H	-1.053700	-0.143244	5.371105
H	-1.565317	-0.270791	7.808537
H	2.676277	0.111015	8.554139
H	0.341149	-0.141457	9.450848
H	6.794490	0.622401	6.746792
H	9.024975	0.886092	5.682544
H	7.185354	0.747281	1.785196
H	9.224130	0.951129	3.200915
H	1.251846	0.115065	3.162682
H	-0.935050	-0.099169	1.939988
H	1.274224	0.097349	-1.760549
H	-0.918490	-0.106610	-0.580092
H	5.744979	0.928895	-3.820391
H	6.765561	1.638451	-2.441342
H	6.392389	-1.350702	-3.175748
H	7.223908	-1.413452	-0.779390
H	8.087104	0.129738	-1.043279
H	9.676389	-1.777885	-1.244699
H	8.605403	-2.594452	-2.405075
H	10.355888	-1.450277	-3.700321
H	10.026962	0.087953	-2.848712
H	8.684629	0.208234	-4.618875

51
2-int$_{2d}$

C	-0.134077	-0.026049	0.055757
C	-0.139217	-0.017193	1.448623
C	1.070340	0.085311	2.129185
C	2.249760	0.175511	1.374437
N	2.254593	0.167353	0.033440
C	1.092181	0.069469	-0.604216

C	3.516681	0.281225	2.051341
C	4.553158	0.366788	2.678608
C	5.747508	0.464638	3.461651
C	5.661346	0.459514	4.877892
C	6.835950	0.554028	5.638555
C	8.074435	0.653100	5.011918
C	8.158609	0.659432	3.617461
C	7.003920	0.565569	2.847027
C	4.379837	0.360101	5.510722
C	3.272522	0.276512	6.003458
C	1.954572	0.179450	6.579799
N	0.921224	0.112229	5.726788
C	-0.304419	0.023615	6.233054
C	-0.570444	-0.003061	7.603830
C	0.503046	0.066327	8.487658
C	1.792172	0.159417	7.972628
I	4.680482	0.431475	-1.603502
C	6.384487	0.657915	-2.911496
C	7.218926	-0.594430	-2.887757
C	7.990602	-0.908987	-1.617545
C	9.283445	-1.561796	-2.107283
C	9.675635	-0.709839	-3.291027
O	8.349945	-0.422318	-3.898530
H	-1.126205	-0.029492	5.512676
H	-1.597698	-0.076717	7.961310
H	2.664983	0.215727	8.623114
H	0.340454	0.048458	9.566414
H	6.763929	0.549371	6.726708
H	8.980350	0.726079	5.615175
H	7.058474	0.570129	1.757624
H	9.130671	0.738923	3.128901
H	1.119253	0.097478	3.220947
H	-1.076545	-0.089064	2.002893
H	1.134796	0.066903	-1.697692
H	-1.058607	-0.104289	-0.516542
H	5.979159	0.852004	-3.912311
H	6.956959	1.524239	-2.555059
H	6.674547	-1.462530	-3.278553
H	7.407607	-1.568629	-0.963882
H	8.208002	0.025502	-1.077793
H	10.072444	-1.559195	-1.346035
H	9.103367	-2.597654	-2.426071
H	10.265470	-1.209013	-4.065494
H	10.116675	0.253145	-3.006995
H	8.312957	0.417329	-4.407739

83

3-dis

C	3.936269	4.503562	5.174821
C	3.765685	4.649122	3.774176
C	4.429489	5.697012	3.126253
C	5.242068	6.576994	3.836993
C	5.427695	6.444165	5.219746
C	4.761708	5.395265	5.867863
C	2.948531	3.729272	3.043383
C	2.270028	2.920794	2.439603
C	1.493595	1.931437	1.753646
C	1.416066	1.921988	0.349667
C	0.699412	0.930766	-0.306564
C	0.040246	-0.072516	0.425691
C	0.095776	-0.045699	1.830968
C	0.817085	0.940157	2.487422

C	-0.650585	-1.132636	-0.249640
C	-1.198530	-2.056625	-0.819870
C	-1.813948	-3.165015	-1.487014
C	-1.814443	-4.455676	-0.897383
C	-2.401482	-5.531492	-1.571641
C	-2.999206	-5.375755	-2.829204
C	-2.991488	-4.094566	-3.397376
C	-2.410664	-3.010501	-2.742659
C	-1.205297	-4.653864	0.384101
C	-0.680251	-4.795670	1.469739
C	-0.065508	-4.906432	2.755901
C	0.539558	-6.087159	3.216854
C	1.121004	-6.110837	4.479236
C	1.095637	-4.972547	5.268958
N	0.507399	-3.851938	4.807565
C	-0.062152	-3.790345	3.595180
I	0.431159	-2.139995	6.045801
C	-3.623522	-6.590146	-3.521770
C	-4.746842	-7.153033	-2.636411
C	6.315698	7.388337	6.034775
C	7.431859	6.573597	6.707907
C	3.259719	3.453545	5.881173
C	2.672924	2.574699	6.480973
C	1.969231	1.559877	7.208714
C	-4.212752	-6.227345	-4.886676
C	-2.543614	-7.665318	-3.723642
C	6.958206	8.465434	5.157850
C	5.462861	8.076805	7.112719
H	1.534281	-4.941434	6.266931
H	1.600565	-7.009153	4.866177
H	0.549545	-6.973804	2.581914
H	-0.525045	-2.850943	3.294013
H	-5.531393	-6.399025	-2.471393
H	-4.368666	-7.476367	-1.655577
H	-5.206070	-8.025900	-3.125230
H	-2.118406	-8.000426	-2.766208
H	-1.722815	-7.284411	-4.350115
H	-2.979838	-8.543220	-4.224281
H	-3.444872	-5.834899	-5.570102
H	-5.013845	-5.478287	-4.797719
H	-4.644558	-7.127881	-5.347246
H	-2.382604	-6.510398	-1.089452
H	-3.443326	-3.922970	-4.373703
H	-2.415999	-2.024714	-3.209094
H	0.654445	0.918128	-1.396156
H	1.933725	2.692886	-0.222280
H	0.871027	0.948173	3.577239
H	-0.432481	-0.808460	2.403669
H	4.304696	5.817957	2.049472
H	5.737345	7.377877	3.289335
H	4.875384	5.255997	6.944467
H	7.600749	8.026388	4.379983
H	6.201292	9.095901	4.667688
H	7.585332	9.117498	5.783299
H	4.651792	8.663485	6.655269
H	6.089695	8.760086	7.705999
H	5.012597	7.347943	7.802534
H	8.078871	7.240903	7.297666
H	7.024916	5.810983	7.387897
H	8.055141	6.066300	5.955903
C	2.627326	0.805155	8.202727
N	2.028074	-0.127290	8.930042

C	0.730262	-0.360133	8.720424
C	-0.020724	0.323184	7.754578
C	0.612986	1.299837	6.981229
H	3.688180	0.988748	8.397401
H	0.255559	-1.119198	9.350186
H	-1.091215	0.134241	7.651134
H	0.062068	1.866306	6.228509

99

3-ts

C	0.362890	-0.134523	0.267460
C	0.363458	-0.082771	1.673872
C	1.002042	0.954978	2.337252
C	1.654549	1.968812	1.611595
C	1.655364	1.917218	0.205920
C	1.017108	0.877759	-0.457919
C	2.339975	3.009320	2.318228
C	2.973970	3.822781	2.963416
C	3.748149	4.722305	3.761797
C	3.944295	4.454805	5.141448
C	4.737486	5.315825	5.905779
C	5.348943	6.448547	5.349542
C	5.139785	6.698901	3.986997
C	4.355328	5.852367	3.205833
C	3.328023	3.305550	5.740081
C	2.773025	2.338148	6.225355
C	2.104373	1.188219	6.762411
C	2.749452	0.346410	7.686975
N	2.192249	-0.750382	8.185625
C	0.957557	-1.067413	7.791594
C	0.222120	-0.299632	6.887039
C	0.801544	0.850198	6.364866
C	6.206444	7.352501	6.239393
C	6.787001	8.534618	5.460178
C	-0.307899	-1.209797	-0.403478
C	-0.893107	-2.132916	-0.937356
C	-1.595747	-3.230795	-1.531485
C	-1.871721	-4.401257	-0.776352
C	-2.573204	-5.458011	-1.365156
C	-3.014583	-5.401813	-2.694165
C	-2.731332	-4.240609	-3.425842
C	-2.036091	-3.175173	-2.858155
C	-1.410523	-4.489208	0.577592
C	-0.979694	-4.512177	1.713279
C	-0.397785	-4.450573	3.020211
C	-0.839149	-5.222718	4.104806
C	-0.209052	-5.078412	5.336660
C	0.839365	-4.169616	5.458555
N	1.255266	-3.437836	4.421512
C	0.669954	-3.565832	3.236872
C	-3.770410	-6.593713	-3.287702
C	-4.180171	-6.342698	-4.740616
C	5.341692	7.898312	7.386928
C	7.367638	6.530316	6.821063
C	-2.865787	-7.835709	-3.240706
C	-5.037492	-6.852712	-2.457177
I	3.110148	-1.716438	4.510586
C	4.874298	-0.257829	4.672184
C	4.419382	0.462858	3.544425
C	4.792095	0.125144	2.144623
C	5.344247	1.349438	1.395270
C	6.414739	2.012797	2.233796

O	5.802890	2.254188	3.490702
H	4.731449	0.204457	5.652577
H	5.772215	-0.871147	4.550160
H	3.641528	1.218261	3.700249
H	3.876671	-0.210753	1.627577
H	5.518438	-0.701377	2.141252
H	5.742284	1.032027	0.422506
H	4.532995	2.068362	1.212154
H	6.747470	2.957998	1.775576
H	7.289254	1.350673	2.346803
H	6.470007	2.383405	4.181761
H	3.762870	0.592028	8.021142
H	-0.787440	-0.599385	6.604302
H	0.262449	1.486518	5.660759
H	-0.523961	-5.663201	6.200687
H	-1.665049	-5.923445	3.975784
H	1.049925	-2.951145	2.414771
H	1.361432	-4.026116	6.408173
H	0.522691	-1.974993	8.220987
H	4.869684	5.086712	6.964908
H	4.210357	6.065961	2.146040
H	5.592533	7.567859	3.510804
H	-2.769073	-6.343828	-0.758138
H	-1.830708	-2.280910	-3.447855
H	-3.055754	-4.150711	-4.461816
H	1.002160	0.990734	3.428091
H	2.160940	2.700240	-0.360822
H	-0.148855	-0.862471	2.239825
H	1.018722	0.842032	-1.547952
H	4.497285	8.486146	6.996294
H	4.936795	7.088394	8.011224
H	5.947059	8.552338	8.033089
H	8.002165	6.125203	6.017954
H	7.993278	7.168070	7.464166
H	7.004893	5.689763	7.430678
H	7.393607	9.154319	6.136631
H	7.436760	8.200087	4.637559
H	5.995094	9.172177	5.039421
H	-1.945179	-7.672657	-3.821379
H	-2.580837	-8.092070	-2.209832
H	-3.394779	-8.700508	-3.669745
H	-5.701614	-5.975170	-2.469829
H	-5.589814	-7.708534	-2.874529
H	-4.795684	-7.085832	-1.409859
H	-4.722911	-7.219883	-5.122158
H	-4.844340	-5.470107	-4.830861
H	-3.305202	-6.181276	-5.387967

105

4-ts-*syn*

C	0.231420	1.576484	7.716039
C	1.502422	1.257654	7.215159
C	1.953483	-0.065429	7.361232
N	1.227786	-1.026652	7.921101
C	0.017888	-0.713989	8.389815
C	-0.517007	0.573265	8.321064
C	2.310539	2.239215	6.546786
C	2.954510	3.092329	5.966614
C	3.697486	4.109472	5.274507
C	3.408524	4.405987	3.918164
C	4.150937	5.404480	3.277825
C	5.172702	6.073692	3.945745

C	5.493843	5.770803	5.275291
C	4.730512	4.791056	5.923814
C	2.452589	3.626189	3.192216
C	1.734920	2.868357	2.565102
C	0.952665	1.930600	1.818108
C	0.162319	0.941973	2.500070
C	-0.553113	-0.022813	1.734226
C	-0.429173	-0.013950	0.300206
C	0.316216	0.974227	-0.322566
C	0.996848	1.946254	0.432116
C	-1.013723	-1.074554	-0.465805
C	-1.449028	-2.039610	-1.065298
C	-1.916785	-3.250414	-1.672121
C	-1.797690	-4.475378	-0.961082
C	-2.260680	-5.660341	-1.538606
C	-2.840098	-5.681353	-2.815280
C	-2.941958	-4.466171	-3.505382
C	-2.490281	-3.271712	-2.946762
C	-1.191613	-4.461355	0.339280
C	-0.665948	-4.336926	1.427764
C	-0.067441	-4.041853	2.695996
C	1.007927	-3.138771	2.751068
N	1.528722	-2.734133	3.903023
C	1.034675	-3.184828	5.060674
C	-0.010705	-4.102859	5.101462
C	-0.570460	-4.540278	3.905057
C	-3.330119	-7.010959	-3.395305
C	-2.150515	-7.993458	-3.472315
C	-1.359319	-0.976808	2.412379
C	0.087018	0.891156	3.918235
C	6.651763	6.438266	6.020939
C	6.132422	7.084182	7.314668
C	7.693758	5.361566	6.366725
C	7.328981	7.518149	5.174189
C	-4.417922	-7.589720	-2.476149
C	-3.916190	-6.839594	-4.798504
I	3.449518	-1.092544	3.993435
C	5.299093	0.210079	4.369877
C	4.906949	1.182190	3.418725
C	5.273692	1.149447	1.979883
C	5.666003	2.552055	1.499747
C	6.757114	3.081417	2.408189
O	6.269045	2.888458	3.727542
H	5.179928	0.479888	5.423231
H	6.169073	-0.407407	4.128253
H	4.122993	1.884012	3.711802
H	4.381958	0.823002	1.418523
H	6.077172	0.417737	1.808413
H	6.002571	2.515602	0.455633
H	4.787530	3.214083	1.551523
H	6.949858	4.151004	2.225941
H	7.693936	2.521943	2.256145
H	6.996542	2.802567	4.362314
H	2.949892	-0.336527	7.000921
H	-1.508260	0.778421	8.725755
H	-0.154097	2.593086	7.623848
H	-0.384454	-4.457826	6.061689
H	-1.403661	-5.244550	3.899198
H	1.436167	-2.732834	1.829855
H	1.481118	-2.794628	5.980854
H	-0.557419	-1.524961	8.845683
H	4.940113	4.531063	6.962975

H	3.929404	5.645349	2.236891
H	5.728579	6.838848	3.404690
H	-2.155830	-6.584723	-0.967983
H	-2.585677	-2.338113	-3.502495
H	-3.382744	-4.434911	-4.501086
H	1.590871	2.705881	-0.077403
H	0.398592	0.980233	-1.409784
H	5.374119	7.850316	7.092994
H	5.684154	6.342397	7.991290
H	6.963455	7.568004	7.850169
H	8.080905	4.886182	5.451802
H	8.542139	5.815013	6.902108
H	7.266789	4.578221	7.010717
H	8.145998	7.975071	5.751423
H	7.762025	7.101191	4.252561
H	6.624652	8.316426	4.895985
H	-1.352976	-7.599313	-4.120345
H	-1.720439	-8.191182	-2.479564
H	-2.490179	-8.953691	-3.889772
H	-5.273653	-6.901804	-2.400032
H	-4.780564	-8.546706	-2.881683
H	-4.035833	-7.776226	-1.461714
H	-4.251807	-7.817102	-5.174674
H	-4.784560	-6.163853	-4.796962
H	-3.170711	-6.444983	-5.505129
C	-1.428967	-0.990180	3.785372
C	-0.684190	-0.058171	4.546892
H	-1.920873	-1.704782	1.824211
H	-2.054173	-1.727729	4.291911
H	-0.728076	-0.089555	5.637503
H	0.651979	1.620998	4.501600

105

4-ts-*anti*

C	0.782204	0.857537	6.333102
C	2.080483	1.188345	6.751086
C	2.706831	0.343421	7.685458
N	2.135521	-0.749750	8.176184
C	0.905095	-1.059619	7.763582
C	0.188211	-0.288632	6.846790
C	2.762652	2.334466	6.223588
C	3.326852	3.300021	5.745447
C	3.952854	4.446879	5.152945
C	3.762279	4.719593	3.773543
C	4.378259	5.847508	3.222340
C	5.166032	6.686541	4.007979
C	5.369796	6.430796	5.370500
C	4.749395	5.300615	5.921901
C	2.986934	3.827366	2.969176
C	2.354558	3.019286	2.314839
C	1.672766	1.985640	1.598103
C	1.034005	0.979694	2.307886
C	0.386481	-0.075080	1.641478
C	0.370385	-0.139265	0.256010
C	1.022785	0.884607	-0.514943
C	1.677347	1.955380	0.160938
C	-0.303493	-1.224052	-0.392332
C	-0.889744	-2.153757	-0.914589
C	-1.590491	-3.252963	-1.507301
C	-1.874730	-4.420748	-0.751203
C	-2.570988	-5.478803	-1.343703
C	-2.998396	-5.426718	-2.677487

C	-2.707639	-4.267759	-3.409986
C	-2.017900	-3.200927	-2.838608
C	-1.424019	-4.503966	0.606355
C	-0.998384	-4.519863	1.744062
C	-0.415968	-4.446351	3.050035
C	0.652461	-3.559684	3.254909
N	1.241276	-3.420483	4.436390
C	0.828350	-4.142109	5.481756
C	-0.221063	-5.051297	5.371876
C	-0.854840	-5.207457	4.143268
C	-3.747178	-6.620807	-3.275448
C	-2.842806	-7.862371	-3.213619
C	2.322069	2.962870	-0.606080
C	1.034471	0.858870	-1.935413
C	6.230901	7.326460	6.265189
C	5.366703	7.875015	7.411851
C	7.384888	6.494800	6.847806
C	6.821619	8.506743	5.490853
C	-5.023709	-6.877098	-2.458678
C	-4.140080	-6.375157	-4.733934
I	3.101688	-1.706958	4.509060
C	4.873546	-0.257840	4.668382
C	4.428122	0.466022	3.538635
C	4.800047	0.123782	2.139885
C	5.347188	1.347745	1.387102
C	6.422427	2.009905	2.220549
O	5.818290	2.246823	3.482341
H	4.731548	0.206594	5.647988
H	5.767691	-0.877269	4.549673
H	3.654171	1.225838	3.691650
H	3.884791	-0.216189	1.625116
H	5.528062	-0.701292	2.138330
H	5.738475	1.032002	0.411021
H	4.534242	2.066258	1.209574
H	6.751139	2.957118	1.763634
H	7.298316	1.348472	2.326238
H	6.489617	2.369412	4.170546
H	3.716638	0.583060	8.034514
H	-0.818479	-0.583114	6.548623
H	0.258280	1.496582	5.620008
H	-0.533610	-5.627552	6.242469
H	-1.681278	-5.909187	4.023462
H	1.030176	-2.953390	2.425667
H	1.354183	-3.990282	6.428072
H	0.457960	-1.963783	8.187549
H	4.877064	5.067642	6.980734
H	4.237543	6.065164	2.162683
H	5.625777	7.553848	3.535519
H	-2.773094	-6.362814	-0.736073
H	-1.806280	-2.308352	-3.428909
H	-3.021352	-4.181115	-4.449524
H	1.036054	1.008220	3.398754
H	-0.116985	-0.850775	2.220289
H	4.527208	8.469392	7.020526
H	4.954872	7.066241	8.033075
H	5.974697	8.523440	8.061175
H	8.018977	6.087699	6.045362
H	8.012902	7.126626	7.494436
H	7.014784	5.655002	7.454007
H	7.430446	9.120456	6.170753
H	7.471471	8.170066	4.669185
H	6.035250	9.150826	5.069648

H	-1.915733	-7.701242	-3.784468
H	-2.569405	-8.114835	-2.178690
H	-3.366751	-8.728864	-3.645411
H	-5.687839	-5.999798	-2.482299
H	-5.570885	-7.734611	-2.879320
H	-4.794064	-7.106251	-1.407757
H	-4.677745	-7.254066	-5.118674
H	-4.803701	-5.503352	-4.835040
H	-3.257675	-6.215472	-5.371552
C	1.666468	1.847712	-2.651087
C	2.317406	2.910474	-1.979725
H	0.531590	0.039361	-2.451143
H	1.666809	1.815547	-3.741674
H	2.819550	3.783165	-0.085766
H	2.814556	3.691792	-2.556563

100

5-ts-*syn*

C	-1.189568	0.881085	11.917619
C	-0.202477	-0.106557	11.791428
C	1.114186	0.306960	11.527995
N	1.432930	1.587683	11.376895
C	0.493613	2.532453	11.488629
C	-0.832910	2.217557	11.768821
C	-0.503299	-1.505957	11.848977
C	-0.721511	-2.700829	11.829300
C	-0.914886	-4.118728	11.729194
C	-0.234841	-4.839189	10.711004
C	-0.412375	-6.224088	10.636928
C	-1.246526	-6.884077	11.536991
C	-1.933960	-6.188737	12.540979
C	-1.749756	-4.800885	12.618201
C	0.597199	-4.131366	9.786605
C	1.276334	-3.452639	9.040243
C	2.037432	-2.556969	8.232242
C	1.592819	-1.191740	8.102374
C	2.380752	-0.231549	7.369787
C	3.621778	-0.625266	6.753599
C	3.989413	-1.950097	6.868325
C	3.211473	-2.898206	7.594031
N	1.843881	0.980224	7.356189
N	0.485673	-0.668735	8.610970
C	4.423160	0.370658	6.124151
C	5.095077	1.280870	5.675963
C	5.914150	2.388627	5.295526
C	5.513997	3.710241	5.621193
C	6.382548	4.773366	5.360034
C	7.643162	4.575414	4.780948
C	8.009051	3.265841	4.442286
C	7.162041	2.190386	4.695246
C	4.247971	3.948137	6.256589
C	3.175556	4.132964	6.800079
C	1.893847	4.351835	7.406508
C	0.710351	3.923516	6.786861
C	-0.499283	4.142703	7.436422
C	-0.486529	4.769918	8.683090
N	0.632865	5.186206	9.278653
C	1.788822	4.989545	8.654670
C	8.568618	5.776335	4.571414
C	8.882394	6.398271	5.942072
C	-2.866602	-6.877788	13.540647
C	-4.282908	-6.301080	13.383780

C	9.886598	5.373360	3.906718
C	7.869405	6.817419	3.683478
C	-2.927225	-8.390162	13.315937
C	-2.361770	-6.613049	14.968170
I	3.697744	2.356699	10.493361
C	5.620139	3.394132	9.817492
C	5.970351	2.345477	8.934684
C	6.788582	1.164426	9.302417
C	7.850515	0.890898	8.227028
C	8.671682	2.146656	8.021180
O	7.720703	3.175524	7.807926
H	5.240529	4.311312	9.357213
H	6.230208	3.520851	10.716501
H	5.463566	2.316042	7.965556
H	6.110020	0.295750	9.348033
H	7.240622	1.306468	10.295256
H	8.483396	0.047695	8.532566
H	7.353216	0.619264	7.282769
H	9.338053	2.049486	7.148497
H	9.285459	2.360205	8.911416
H	8.099596	4.051674	7.974464
H	2.694795	5.345343	9.155410
H	-1.442338	3.825676	6.990211
H	0.751987	3.422063	5.818651
H	-1.574305	3.011986	11.854561
H	-2.223338	0.594765	12.115569
H	1.913076	-0.432467	11.418213
H	0.808118	3.568473	11.331944
H	-1.423702	4.943819	9.220267
H	6.057598	5.778711	5.634100
H	7.472706	1.176444	4.438549
H	8.974376	3.065053	3.978579
H	-2.261527	-4.221561	13.388638
H	0.106350	-6.785694	9.859110
H	-1.356868	-7.963560	11.440360
H	4.921030	-2.282512	6.409193
H	3.574705	-3.923659	7.667502
H	7.623679	6.390721	2.699325
H	6.939399	7.186404	4.139648
H	8.533360	7.681402	3.527679
H	9.390105	5.669415	6.593208
H	9.547921	7.266540	5.819206
H	7.969048	6.741957	6.450093
H	10.515026	6.265956	3.772767
H	10.450165	4.655696	4.521650
H	9.721472	4.925721	2.915313
H	-1.345561	-7.012803	15.105418
H	-2.342461	-5.538173	15.200164
H	-3.025803	-7.103408	15.696346
H	-4.666163	-6.473430	12.366591
H	-4.966331	-6.787795	14.096363
H	-4.304550	-5.219057	13.580090
H	-3.603993	-8.841219	14.056274
H	-3.311444	-8.637494	12.315062
H	-1.938593	-8.858600	13.433696
S	0.454258	0.902423	8.196838

100

5-ts-*anti*

C	0.641114	-3.543667	3.206691
C	-0.415665	-4.445720	3.007703
C	-0.834080	-5.216753	4.102047

C	-0.192068	-5.056029	5.325732
C	0.845582	-4.132872	5.429713
N	1.238545	-3.401080	4.383617
C	-1.007295	-4.526480	1.706156
C	-1.441890	-4.518094	0.571730
C	-1.899136	-4.437741	-0.783954
C	-1.608923	-3.274713	-1.545748
C	-2.037943	-3.222049	-2.876102
C	-2.739794	-4.284493	-3.440894
C	-3.039605	-5.437640	-2.702877
C	-2.606694	-5.491042	-1.370528
C	-0.904356	-2.180142	-0.951113
C	-0.320129	-1.261582	-0.408418
C	0.344073	-0.195494	0.267565
C	0.336249	-0.078728	1.643364
C	0.988102	0.987907	2.323896
C	1.673125	1.983641	1.656576
C	1.717778	1.897649	0.220477
C	1.057105	0.815401	-0.470179
C	2.349686	3.028030	2.350673
C	2.983127	3.847266	2.989166
C	3.755596	4.745560	3.787873
C	3.952841	4.470777	5.166179
C	4.746321	5.328878	5.932772
C	5.355932	6.464503	5.379744
C	5.145336	6.721069	4.018362
C	4.361011	5.877618	3.234107
C	3.338746	3.316846	5.757933
C	2.784684	2.344228	6.233682
C	2.116575	1.189261	6.760500
C	2.764696	0.335202	7.671641
N	2.207083	-0.765571	8.160957
C	0.969059	-1.074482	7.770994
C	0.230850	-0.294934	6.878826
C	0.810813	0.858879	6.366146
C	6.214610	7.364595	6.272124
C	7.378757	6.540312	6.845016
C	-3.804824	-6.624786	-3.293517
C	-5.077374	-6.867022	-2.466249
C	6.790980	8.552229	5.498271
C	5.352536	7.902133	7.425632
C	-4.206990	-6.376865	-4.749087
C	-2.912420	-7.875237	-3.236961
I	3.096049	-1.689140	4.466447
C	4.875506	-0.246335	4.645737
C	4.428294	0.490416	3.526039
C	4.803538	0.167543	2.123000
C	5.350714	1.399133	1.382084
C	6.417879	2.060371	2.226764
O	5.807062	2.282638	3.487967
H	4.732877	0.203809	5.631767
H	5.767591	-0.867081	4.518910
H	3.651771	1.245018	3.689822
H	3.890203	-0.170389	1.604263
H	5.532930	-0.656280	2.113164
H	5.752880	1.089169	0.408398
H	4.539081	2.117374	1.197099
H	6.742598	3.013264	1.779114
H	7.297059	1.403026	2.330794
H	6.474950	2.409255	4.178793
H	3.780647	0.574232	8.002812
H	-0.781419	-0.588380	6.599172

H	0.269149	1.505147	5.673157
H	-0.488593	-5.639964	6.196802
H	-1.651071	-5.930197	3.986550
H	1.002610	-2.928501	2.376586
H	1.378523	-3.977789	6.371569
H	0.533659	-1.984887	8.193738
H	4.880898	5.095308	6.990566
H	4.214638	6.093774	2.175093
H	5.597289	7.592318	3.545739
H	-2.814408	-6.371667	-0.759976
H	-1.819085	-2.333238	-3.469094
H	-3.056342	-4.198713	-4.479578
H	0.940629	1.020318	3.413452
N	2.342520	2.731658	-0.600271
H	-0.197017	-0.826136	2.232596
N	1.202555	0.878533	-1.786811
H	4.505576	8.490361	7.041204
H	4.951728	7.088160	8.047259
H	5.959016	8.553944	8.072942
H	8.011264	6.141080	6.037403
H	8.005430	7.175235	7.489879
H	7.019147	5.695593	7.450722
H	7.398071	9.169017	6.176911
H	7.439403	8.223730	4.672209
H	5.996824	9.190718	5.083207
H	-1.987952	-7.723915	-3.814630
H	-2.634259	-8.129625	-2.203758
H	-3.448249	-8.736667	-3.664232
H	-5.732282	-5.982696	-2.484888
H	-5.637012	-7.718697	-2.882260
H	-4.841796	-7.098571	-1.417190
H	-4.758135	-7.250078	-5.127616
H	-4.861206	-5.497503	-4.846067
H	-3.328252	-6.228743	-5.394462
S	2.100684	2.190448	-2.111872

6. REFERENCES

1. Carlsson, A.-C. C.; Grafenstein, J.; Laurila, J. L.; Bergquist, J.; Erdelyi, M., Symmetry of $[N\text{-}X\text{-}N]^+$ halogen bonds in solution. *Chem. Commun.* **2012**, *48* (10), 1458-1460.
2. Carlsson, A.-C. C.; Gräfenstein, J.; Budnjo, A.; Laurila, J. L.; Bergquist, J.; Karim, A.; Kleinmaier, R.; Brath, U.; Erdelyi, M., Symmetric halogen bonding is preferred in solution. *J. Am. Chem. Soc.* **2012**, *134* (12), 5706-5715.
3. Carlsson, A.-C. C.; Mehmeti, K.; Uhrbom, M.; Karim, A.; Bedin, M.; Puttreddy, R.; Kleinmaier, R.; Neverov, A. A.; Nekoueishahraki, B.; Gräfenstein, J.; Rissanen, K.; Erdelyi, M., Substituent Effects on the $[N–I–N]^+$ Halogen Bond. *J. Am. Chem. Soc.* **2016**, *138* (31), 9853-9863.
4. Thorson, R. A.; Woller, G. R.; Driscoll, Z. L.; Geiger, B. E.; Moss, C. A.; Schlapper, A. L.; Speetzen, E. D.; Bosch, E.; Erdelyi, M.; Bowling, N. P., Intramolecular Halogen Bonding in Solution: N-15, C-13, and F-19 NMR Studies of Temperature and Solvent Effects. *Eur. J. Org. Chem.* **2015**, *2015* (8), 1685-1695.
5. Wegner, H. A.; Reisch, H.; Rauch, K.; Demeter, A.; Zachariasse, K. A.; Meijere, A.; Scott, L. T., Oligoindenopyrenes: a new class of polycyclic aromatics. *J. Org. Chem.* **2006**, *71* (24), 9080-7.
6. Vang, H. G.; Driscoll, Z. L.; Robinson, E. R.; Green, C. E.; Bosch, E.; Bowling, N. P., Conjugated, trans-Spanning Ligands as Models for Multivalent p-Phenyleneethynylenes. *Eur. J. Org. Chem.* **2016**, *2016* (5), 891-895.
7. da Cunha, T. T.; Oliveira, W. X. C.; Pinheiro, C. B.; Pedroso, E. F.; Nunes, W. C.; Pereira, C. L. M., Alkaline Ion-Modulated Solid-State Supramolecular Organization in Mixed Organic/Metallorganic Compounds Based on 1,1 '-Ethylenebis(4-aminopyridinium) Cations and Bis(oxamate)cuprate(II) Anions. *Cryst. Growth Des.* **2016**, *16* (2), 900-907.
8. Kathalikkattil, A. C.; Tharun, J.; Roshan, R.; Soek, H. G.; Park, D. W., Efficient route for oxazolidinone synthesis using heterogeneous biopolymer catalysts from unactivated alkyl aziridine and CO_2 under mild conditions. *Appl. Catal.* **2012**, *447*, 107-114.
9. Zhao, Y.; Truhlar, D. G., The M06 suite of density functionals for main group thermochemistry, thermochemical kinetics, noncovalent interactions, excited states, and transition elements: two new functionals and systematic testing of four M06-class functionals and 12 other functionals. *Theor. Chem. Account.* **2008**, *120* (1-3), 215-241.
10. Weigend, F.; Ahlrichs, R., Balanced basis sets of split valence, triple zeta valence and quadruple zeta valence quality for H to Rn: Design and assessment of accuracy. *Phys. Chem. Chem. Phys.* **2005**, *7* (18), 3297-3305.
11. Goerigk, L.; Grimme, S., A thorough benchmark of density functional methods for general main group thermochemistry, kinetics, and noncovalent interactions. *Phys. Chem. Chem. Phys.* **2011**, *13* (14), 6670-6688.
12. Kozuch, S.; Martin, J. M. L., Halogen Bonds: Benchmarks and Theoretical Analysis. *J. Chem. Theory Comput.* **2013**, *9* (4), 1918-1931.
13. Tomasi, J.; Mennucci, B.; Cances, E., The IEF version of the PCM solvation method: an overview of a new method addressed to study molecular solutes at the QM ab initio level. *J. Mol. Struct.* **1999**, *464* (1-3), 211-226.
14. Marenich, A. V.; Cramer, C. J.; Truhlar, D. G., Universal Solvation Model Based on Solute Electron Density and on a Continuum Model of the Solvent Defined by the Bulk Dielectric Constant and Atomic Surface Tensions. *J. Phys. Chem. B* **2009**, *113* (18), 6378-6396.
15. Engelage, E.; Schulz, N.; Heinen, F.; Huber, S. M.; Truhlar, D. G.; Cramer, C. J., Refined SMD Parameters for Bromine and Iodine Accurately Model Halogen-Bonding Interactions in Solution. *Chem. Eur. J.* **2018**, *24* (60), 15983-15987.
16. Grimme, S., Supramolecular binding thermodynamics by dispersion-corrected density functional theory. *Chem. Eur. J.* **2012**, *18* (32), 9955-9964.
17. M. J. Frisch, G. W. T., H. B. Schlegel, G. E. Scuseria, M. A. Robb, J. R. Cheeseman, G. Scalmani, V. Barone, G. A. Petersson, H. Nakatsuji, X. Li, M. Caricato, A. V. Marenich, J. Bloino, B. G. Janesko, R. Gomperts, B. Mennucci, H. P. Hratchian, J. V. Ortiz, A. F. Izmaylov, J. L. Sonnenberg, D. Williams-Young, F. Ding, F. Lipparini, F. Egidi, J. Goings, B. Peng, A. Petrone, T. Henderson, D. Ranasinghe, V. G. Zakrzewski, J. Gao, N. Rega, G. Zheng, W. Liang, M. Hada, M. Ehara, K. Toyota, R. Fukuda, J. Hasegawa, M. Ishida, T. Nakajima, Y. Honda, O. Kitao, H. Nakai, T. Vreven, K. Throssell, J. A. Montgomery, Jr., J. E. Peralta, F. Ogliaro, M. J. Bearpark, J. J. Heyd, E. N. Brothers, K. N. Kudin, V. N. Staroverov, T. A. Keith, R. Kobayashi, J. Normand, K. Raghavachari, A. P. Rendell, J. C. Burant, S. S. Iyengar, J. Tomasi, M. Cossi, J. M. Millam, M. Klene, C. Adamo, R. Cammi, J. W. Ochterski, R. L. Martin, K. Morokuma, O. Farkas, J. B. Foresman, D. J. Fox *Gaussian 16, Revision A.03*, Gaussian, Inc.: Wallingford CT, 2016.
18. Legault, C. Y. *CYLview, 1.0b*, Universite de Sherbrooke, 2009.
19. Contreras-Garcia, J.; Johnson, E. R.; Keinan, S.; Chaudret, R.; Piquemal, J. P.; Beratan, D. N.; Yang, W., NCIPLOT: a program for plotting non-covalent interaction regions. *J. Chem. Theory Comput.* **2011**, *7* (3), 625-632.
20. Johnson, E. R.; Keinan, S.; Mori-Sanchez, P.; Contreras-Garcia, J.; Cohen, A. J.; Yang, W., Revealing noncovalent interactions. *J. Am. Chem. Soc.* **2010**, *132* (18), 6498-6506.
21. Humphrey, W.; Dalke, A.; Schulten, K., VMD: Visual molecular dynamics. *J. Mol. Struct.* **1996**, *14* (1), 33-38.
22. Chai, J. D.; Head-Gordon, M., Long-range corrected hybrid density functionals with damped atom-atom dispersion corrections. *Phys. Chem. Chem. Phys.* **2008**, *10* (44), 6615-6620.
23. Chai, J. D.; Head-Gordon, M., Systematic optimization of long-range corrected hybrid density functionals. *J. Chem. Phys.* **2008**, *128* (8), 084106.
24. Grimme, S., Semiempirical GGA-type density functional constructed with a long-range dispersion correction. *J. Comput. Chem.* **2006**, *27* (15), 1787-1799.
